# Gastric Neoplasm Risk with DPP-4 Inhibitors, GLP-1 Receptor Agonists, and SGLT2 Inhibitors: Network Meta-Analysis of Randomized Trials

**DOI:** 10.3390/ijms27062619

**Published:** 2026-03-13

**Authors:** Chao-Ming Hung, Chih-Wei Hsu, Bing-Syuan Zeng, Mein-Woei Suen, Jiann-Jy Chen, Bing-Yan Zeng, Andre F. Carvalho, Brendon Stubbs, Yen-Wen Chen, Tien-Yu Chen, Shih-Pin Hsu, Hung-Yu Wang, Chih-Sung Liang, Yu-Kang Tu, Ping-Tao Tseng

**Affiliations:** 1 Division of General Surgery, Department of Surgery, E-Da Cancer Hospital, I-Shou University, Kaohsiung 824, Taiwan; ed100647@edah.org.tw; 2 School of Medicine, College of Medicine, I-Shou University, Kaohsiung 824, Taiwan; a.pin.hsu@gmail.com; 3 Department of Psychiatry, Kaohsiung Chang Gung Memorial Hospital and Chang Gung University College of Medicine, Kaohsiung 833, Taiwan; harwicacademia@gmail.com; 4 Department of Internal Medicine, E-Da Cancer Hospital, I-Shou University, Kaohsiung 824, Taiwan; b95401072@ntu.edu.tw; 5 Department of Psychology, College of Medical and Health Science, Asia University, Taichung 413, Taiwan; blake@asia.edu.tw; 6 Gender Equality Education and Research Center, Asia University, Taichung 413, Taiwan; 7 Department of Medical Research, Asia University Hospital, Asia University, Taichung 413, Taiwan; 8 Department of Medical Research, China Medical University Hospital, China Medical University, Taichung 406, Taiwan; 9 Prospect Clinic for Otorhinolaryngology & Neurology, Kaohsiung 811, Taiwan; jiannjy@yahoo.com.tw (J.-J.C.); kevinachen0527@gmail.com (Y.-W.C.); 10 Department of Otorhinolaryngology, E-Da Cancer Hospital, I-Shou University, Kaohsiung 824, Taiwan; 11 Institute of Biomedical Sciences, National Sun Yat-sen University, Kaohsiung 804, Taiwan; holdinggreat@yahoo.com.tw; 12 Department of Internal Medicine, E-Da Dachang Hospital, I-Shou University, Kaohsiung 807, Taiwan; 13 Innovation in Mental and Physical Health and Clinical Treatment (IMPACT) Strategic Research Centre, School of Medicine, Barwon Health, Deakin University, Geelong, VIC 3216, Australia; canaldasaudemental@gmail.com; 14 Department of Psychological Medicine, Institute of Psychiatry, Psychology and Neuroscience, King’s College London, London WC2R 2LS, UK; brendon.stubbs@kcl.ac.uk; 15 Comprehensive Center for Clinical Neurosciences and Mental Health, Medical University of Vienna, 1090 Vienna, Austria; 16 Clinical Division of Social Psychiatry, Department of Psychiatry and Psychotherapy, Medical University of Vienna, 1090 Vienna, Austria; 17 Department of Psychiatry, Tri-Service General Hospital, Taipei 114, Taiwan; verducciwol@gmail.com; 18 Department of Psychiatry, College of Medicine, National Defense Medical University, Taipei 114, Taiwan; 19 Department of Neurology, E-Da Hospital, I-Shou University, Kaohsiung 824, Taiwan; 20 Kaohsiung Municipal Kai-Syuan Psychiatric Hospital, Kaohsiung 802, Taiwan; hywang1975@gmail.com; 21 Department of Psychiatry, Beitou Branch, Tri-Service General Hospital, School of Medicine, National Defense Medical University, Taipei 114, Taiwan; lcsyfw@gmail.com; 22 Department of Psychiatry, National Defense Medical University, Taipei 114, Taiwan; 23 Institute of Health Data Analytics & Statistics, College of Public Health, National Taiwan University, No. 17, Xuzhou Road, Taipei 100, Taiwan; 24 Department of Dentistry, National Taiwan University Hospital, Taipei 100, Taiwan; 25 Institute of Precision Medicine, National Sun Yat-sen University, No. 70 Lienhai Rd., Kaohsiung 804, Taiwan; 26 School of Medicine, College of Medicine, National Sun Yat-sen University, Kaohsiung 804, Taiwan

**Keywords:** DPP-4 inhibitor, GLP-1 receptor agonist, SGLT2 inhibitor, gastric neoplasms, semaglutide, diabetes mellitus

## Abstract

Whether the risk of gastric neoplasm is modified by newer glucose-lowering therapies—dipeptidyl peptidase-4 inhibitors (DPP4is), glucagon-like peptide-1 receptor agonists (GLP1RAs), and sodium–glucose cotransporter 2 inhibitors (SGLT2is)—remains uncertain. Given their global uptake and long-term use in populations already predisposed to malignancy, decision-grade comparative safety evidence is needed. We conducted a systematic review and network meta-analysis (NMA) of randomized controlled trials (RCTs) in adults without baseline gastric neoplasms. PubMed, Embase, Cochrane CENTRAL, Web of Science, ClinicalTrials.gov, ClinicalKey, ProQuest, and ScienceDirect were searched from inception to 10 January 2026, without language restrictions. The primary outcome was incident gastric neoplasms (benign or malignant). Random-effects frequentist NMA estimated risk ratios (RRs) with 95% confidence intervals (CIs); Bayesian NMA served as sensitivity analysis. Certainty of evidence was assessed using GRADE adapted for NMA (PROSPERO CRD420261282728). Fifty-two RCTs (171,165 participants; mean age 63.6 years; 36.9% women; mean follow-up 141.8 weeks) were included. At the class level, GLP1RAs were associated with lower gastric neoplasm risk versus controls (RR = 0.51, 95% CI = 0.28–0.92), whereas DPP4is were associated with higher risk (RR = 1.77, 95% CI = 1.09–2.85). These signals persisted in prespecified subgroup analyses among participants with diabetes mellitus, in trials with duration ≥52 weeks (GLP1RA: RR = 0.52, 95% CI = 0.28–0.95; DPP4i: RR = 2.05, 95% CI = 1.19–3.55), and in older populations (age ≥60 years; DPP4i: RR = 2.08, 95% CI = 1.15–3.77). No class showed a significant association in younger participants (<60 years) or shorter trials (<52 weeks). Across available RCT evidence, GLP1RA prescription generally had a relatively lower gastric neoplasm risk than controls. In contrast, among patients with diabetes mellitus receiving longer-term therapy, GLP1RAs may be the preferable option from the perspective of gastric neoplasm risk, while DPP4is warrant heightened vigilance and mechanistic clarification. These findings support improved neoplasms ascertainment in future trials rather than immediate prescribing changes.

## 1. Introduction

Newer glucose-lowering therapies—dipeptidyl peptidase-4 inhibitors (DPP4is), glucagon-like peptide-1 receptor agonists (GLP1RAs), and sodium–glucose cotransporter 2 inhibitors (SGLT2is)—are now prescribed worldwide and operate through pharmacodynamic pathways that differ meaningfully from earlier antidiabetic agents, while providing substantial metabolic and cardiometabolic benefit for many patients [1]. As clinical use has broadened in both diabetes and related metabolic conditions, post-marketing experience and secondary analyses of large trials have highlighted several unanticipated safety signals, including the possibility of altered malignancy risk profiles [2,3,4,5]. However, most randomized controlled trials (RCTs) evaluating these therapies were designed primarily to quantify glycemic efficacy or major cardiovascular and renal outcomes rather than to systematically detect tumor outcomes. Consequently, gastric tumor events are typically rare, often captured only as coded adverse events, and individual trials are rarely powered to provide precise estimates of neoplasms incidence. In addition, direct head-to-head comparisons across multiple contemporary regimens are uncommon, leaving clinicians and guideline panels with fragmented evidence when attempting to balance long-term benefit against uncertain oncologic safety.

Gastric tumor remains among the leading causes of neoplasm-related mortality globally [6]. Importantly, patients with diabetes mellitus—who constitute the principal target population for these medications—have a higher incidence of gastric tumors than the general population [7]. This baseline susceptibility complicates causal inference and heightens clinical concern: even a modest drug-associated relative effect could translate into meaningful absolute harm in high-risk groups, whereas a protective effect could offer an unanticipated public-health advantage. Several conventional pairwise meta-analyses have assessed digestive neoplasms as a composite outcome [8,9] or examined gastric neoplasm outcomes in subgroup analyses [10,11], but their conclusions have remained inconsistent. Pairwise meta-analysis may improve precision compared with single trials, yet it is intrinsically constrained when evidence is distributed across numerous competing interventions and when direct comparisons are sparse or absent. Network meta-analysis (NMA) provides a structured framework to integrate direct and indirect evidence across a treatment network, enabling coherent comparative estimates among multiple regimens and offering a more informative basis for comparative safety assessment when head-to-head data are limited.

To our knowledge, no prior NMA has comprehensively quantified gastric tumor risk associated with newer glucose-lowering therapies while explicitly considering three clinically consequential dimensions: diabetes mellitus status, study duration, and patient age. Building on our team’s experience in NMAs evaluating adverse and clinically important outcomes related to these therapies—including neurodegenerative disorders [12,13,14], altered oncologic outcomes [2,3,4,5], intestinal obstruction [15], and antiseptic property [16]—we undertook a large, protocol-driven NMA to clarify whether DPP4is, GLP1RAs, and SGLT2is are associated with increased or decreased risks of incident gastric tumor events among adults without pre-existing gastric tumors.

## 2. Results

### 2.1. Study Selection and Characteristics

**Figure 1:**
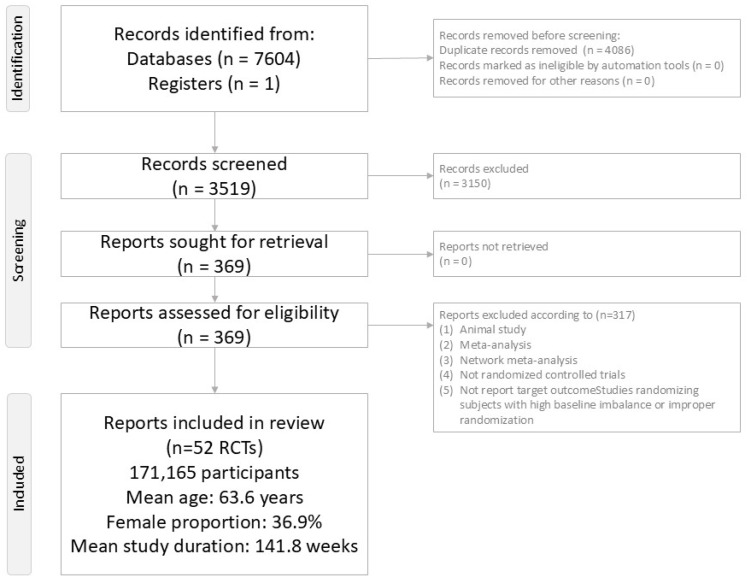
PRISMA2020 flowchart of current network meta-analysis.

The PRISMA flow diagram is presented in Figure 1. After duplicate removal and exclusion of 317 records during screening (Table S4), 52 RCTs met eligibility criteria and were included in the final evidence network (Table S5) [17,18,19,20,21,22,23,24,25,26,27,28,29,30,31,32,33,34,35,36,37,38,39,40,41,42,43,44,45,46,47,48,49,50,51,52,53,54,55,56,57,58,59,60,61,62,63,64,65,66,67,68]. Across these trials, 171,165 participants were enrolled (mean age 63.6 years; 36.9% women), with a mean follow-up of 141.8 weeks, providing substantial person-time for the identification of incident gastric neoplasm events.

The treatment network included:

- DPP4i: alogliptin, linagliptin, saxagliptin, sitagliptin, and vildagliptin;
- GLP1RA: dulaglutide, exenatide, liraglutide, lixisenatide, and semaglutide (injectable and oral formulations);
- SGLT2i: bexagliflozin, canagliflozin, dapagliflozin, empagliflozin, ertugliflozin, and sotagliflozin;
- Dual agonist: tirzepatide (GIP/GLP-1 receptor agonist).

Although certain DPP4is (omarigliptin and teneligliptin), GLP1RAs (albiglutide, efpeglenatide, and orforglipron), triple GIP/GLP-1/glucagon receptor agonists (e.g., retatrutide), mitochondrial bioenergetics modulators (e.g., imeglimin), glucokinase activators (e.g., dorzagliatin), amylin analogues (e.g., petrelintide), and anti-CD3 monoclonal antibodies (e.g., teplizumab) were prespecified in the search scope, eligible RCTs did not report gastric tumor outcomes in a manner permitting quantitative synthesis.

### 2.2. Primary Outcome: Gastric Tumor Risk (Benign or Malignant)—Class Level

**Figure 2:**
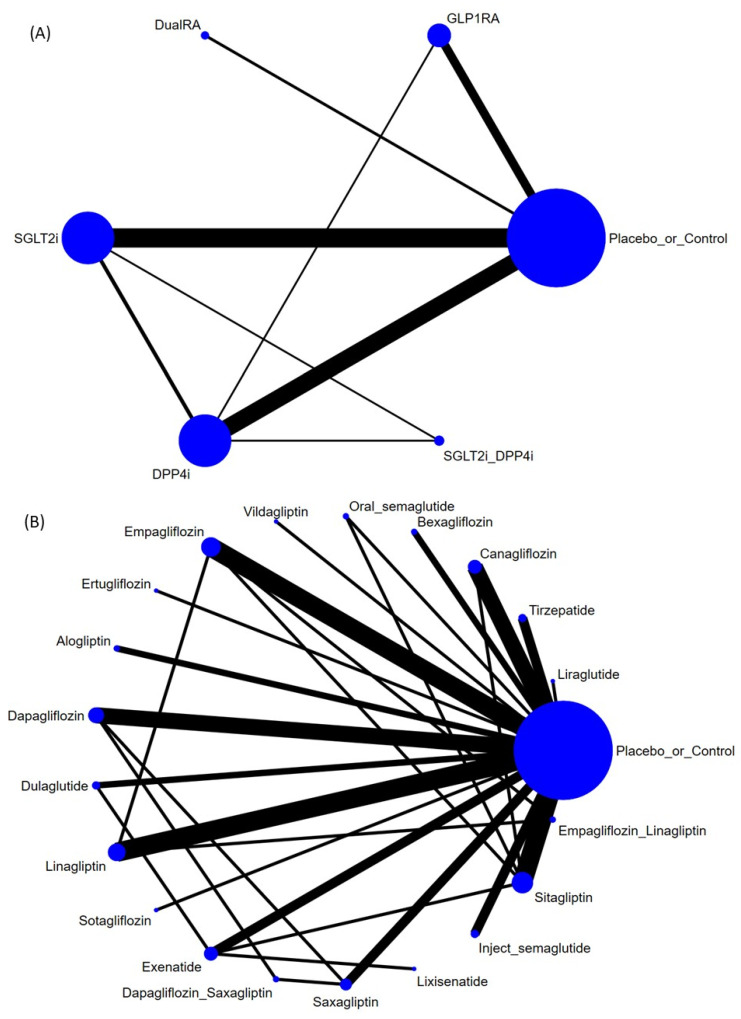

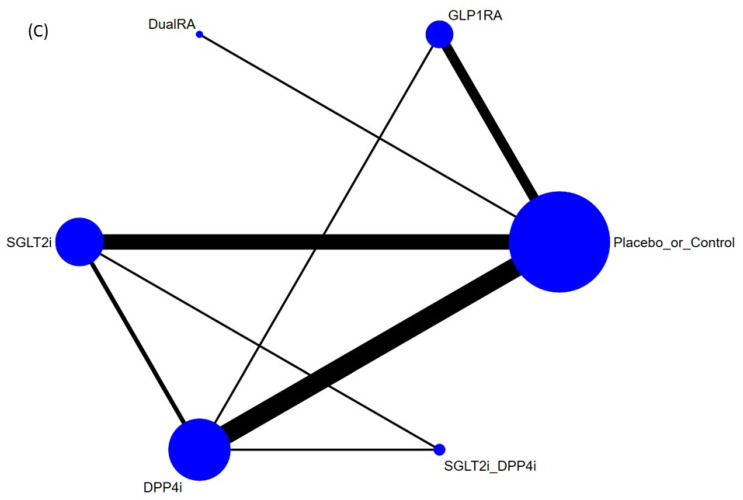
Network structure of the primary outcome: gastric tumor risk (benign or malignant) in (**A**) class-level, (**B**) regimen-level, and (**C**) subgroup focusing on participants with diabetes mellitus. Overall structure of the network meta-analysis. The lines between nodes represent direct comparisons in various trials, and the size of each circle is proportional to the number of participants in each specific treatment. The thickness of the lines is proportional to the number of trials connected to the network.

**Figure 3:**
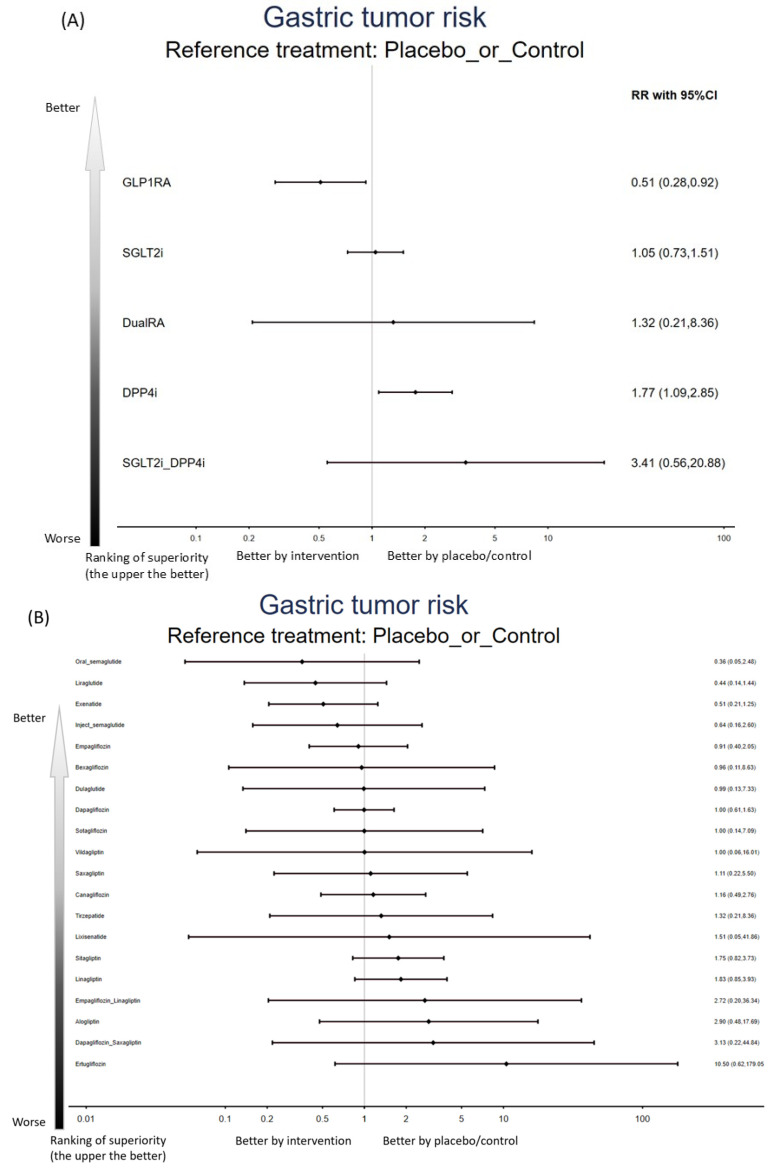

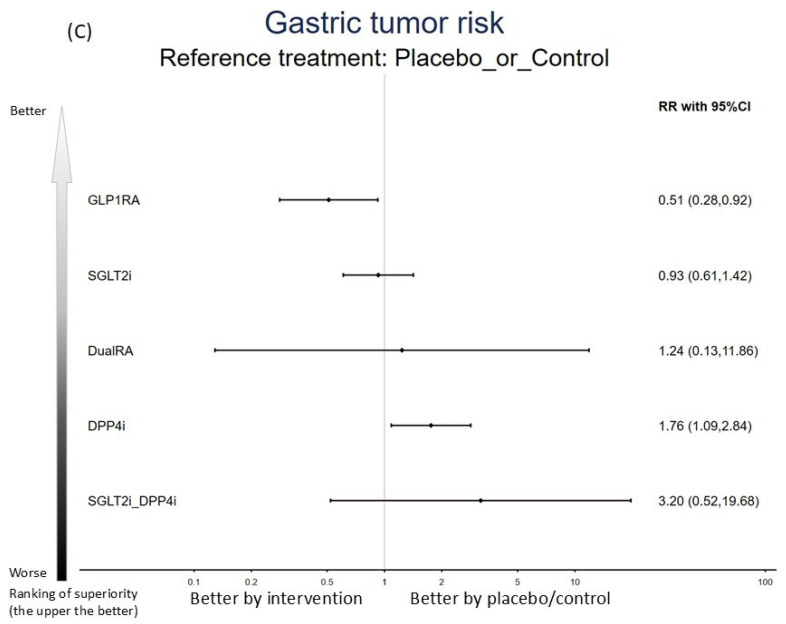
Forest plot of primary outcome: gastric tumor risk (benign or malignant) in (**A**) class-level, (**B**) regimen-level, and (**C**) subgroup focusing on participants with diabetes mellitus. When the effect size (expressed as risk ratio) is less than 1, the specified treatment is associated with a lower risk than the placebo/controls.

In the primary class-level NMA, GLP1RAs (event/subjects = 0.09%) were associated with a significantly lower risk of gastric neoplasm compared with controls (event/subjects = 0.13%) (RR = 0.51, 95%CIs = 0.28 to 0.92). In contrast, DPP4is (event/subjects = 0.21%) were associated with a significantly higher risk of gastric tumor (benign or malignant) than controls (RR = 1.77, 95%CIs = 1.09 to 2.85). DualRA (event/subjects = 0.16%), SGLT2is (event/subjects = 0.14%), and SGLT2i+DPP4is (event/subjects = 0.28%) did not have a statistically significant difference in comparison with controls (Figure 2A, Figure 3A and Figure S3). Additionally, GLP1RAs were associated with lower gastric neoplasm risk than SGLT2is (RR = 0.49, 95%CIs = 0.24 to 0.98) and DPP4is (RR = 0.29, 95%CIs = 0.14 to 0.61) (Table 1).

### 2.3. Primary Outcome: Gastric Tumor Risk (Benign or Malignant)—Regimen Level

When the network was examined at the regimen level, no individual regimen showed a statistically significant difference in gastric neoplasms risk compared with control (Figure 2B, Figure 3B, and Table 2).

.

### 2.4. Primary Outcome: Gastric Tumor Risk (Benign or Malignant)—Subgroup Focusing on Participants with Various Baseline Diseases

When analyses were restricted to RCTs enrolling participants with type 2 diabetes mellitus, findings were consistent with the overall network: GLP1RAs remained associated with a significantly lower risk of gastric tumor versus controls (RR = 0.51, 95%CIs = 0.28 to 0.92), whereas DPP4is remained associated with a significantly higher risk versus controls (RR = 1.76, 95%CIs = 1.09 to 2.84) (Figure 2C and Figure 3C, and Table 3).

On the other hand, the subgroup analysis of obesity or chronic renal insufficiency could not be conducted due to the lack of a sufficient dataset.

### 2.5. Primary Outcome: Gastric Tumor Risk (Benign or Malignant)—Subgroup of Study Duration Stratification

Among trials with study duration **≥ 52 weeks**, GLP1RAs were again associated with lower gastric tumor risk compared with controls (RR = 0.52, 95%CIs = 0.28 to 0.95), while DPP4is were associated with higher risk compared with controls (RR = 2.05, 95%CIs = 1.19 to 3.55) (Figures S1A and S2A, and Table S6A).

In contrast, among trials with study duration **< 52 weeks**, no regimen demonstrated a statistically significant difference versus controls (Figures S1B and S2B and Table S6B).

### 2.6. Primary Outcome: Gastric Tumor Risk (Benign or Malignant)—Subgroup of Age-Stratification

In trials enrolling older populations (mean age **≥ 60 years**), DPP4is were associated with a significantly higher gastric tumor risk than controls (RR = 2.08, 95%CIs = 1.15 to 3.77), whereas GLP1RAs were associated with a significantly lower gastric tumor risk than DPP4is (RR = 0.24, 95%CIs = 0.10 to 0.61) (Figures S1C and S2C, and Table S6C).

In trials enrolling younger populations (mean age **< 60 years**), no regimen demonstrated a statistically significant difference versus controls (Figures S1D and S2D, and Table S6D).

### 2.7. Secondary Outcome: Helicobacter Pylori Risk

In the secondary outcome, no regimen demonstrated a significantly different risk of *Helicobacter pylori* infection versus controls (Figures S1E and S2E, and Table S6E).

### 2.8. Treatment Acceptability: Drop-Out Rate

For acceptability, lower drop-out rates were observed for SGLT2is plus DPP4is, DPP4is, and SGLT2is compared to controls (Figures S1F and S2F, and Table S6F).

### 2.9. Primary Outcome: Gastric Tumor Risk (Benign or Malignant)—Dose Level

When the network was examined at the dose level, no individual regimen at any dosage showed a statistically significant difference in gastric neoplasms risk compared with controls, which might have resulted from the widening confidence intervals related to reduced sample sizes (Figures S1G and S2G).

### 2.10. Publication Bias, Ranking, Heterogeneity, Inconsistency, and Sensitivity Analyses

Comparison-adjusted funnel plots did not suggest marked asymmetry (Figure S4), and Egger’s tests were not statistically significant (Figure S5), indicating limited evidence of small-study effects or publication bias. SUCRA rankings are provided in Table S7. Between-study heterogeneity (τ^2^) was generally modest (Table S8). Inconsistency assessments using node-splitting, loop-specific, and design-by-treatment interaction approaches did not reveal substantial disagreement between direct and indirect evidence (Table S9A,B), supporting network coherence. Bayesian sensitivity analyses were aligned with frequentist findings: GLP1RAs were associated with lower gastric tumor risk versus controls, whereas DPP4is were associated with higher risk versus controls. By better accommodating zero-event trials, Bayesian models reinforced robustness under sparse-event conditions (Figure S6).

### 2.11. Risk of Bias and Certainty of Evidence

Among the 52 included RCTs, 32/52 (61.5%) were judged low risk of bias, 16/52 (30.8%) raised some concerns, and 4/52 (7.7%) were judged high risk using RoB 2.0 (Figure S7). Using GRADE adapted for NMA, certainty was rated as moderate to high for most comparisons involving gastric tumor outcomes (Table S10).

## 3. Discussion

To our knowledge, this is the first NMA designed specifically to evaluate gastric neoplasm risk associated with newer antidiabetic therapies while incorporating prespecified stratification by diabetes mellitus status, trial duration, and age. Using moderate-to-high certainty evidence under GRADE, we observed a consistent signal: DPP4is were associated with a significantly higher risk of gastric tumor than controls in the overall network and in clinically key subgroups—participants with diabetes mellitus, trials with longer follow-up (≥52 weeks), and older populations (≥60 years). By contrast, GLP1RAs were associated with a significantly lower risk of gastric tumor than controls in the overall network and in subgroups defined by diabetes mellitus and longer trial duration (≥52 weeks). In younger populations (<60 years) and shorter-duration trials (<52 weeks), we did not detect statistically significant differences in gastric tumor risk for any regimen class versus control, a finding that may offer partial reassurance in time-limited prescribing contexts.

The main findings of the current NMA were the divergent risks of gastric tumors related to GLP1RAs and DPP4is, which were different from the results of previous studies. Specifically, several prior meta-analyses pooled heterogeneous digestive malignancies into a single composite, which may dilute tumor site-specific signals and obscure mechanistic differences that matter for gastric carcinogenesis [8,9]. By focusing directly on gastric tumors, we addressed an outcome with distinct epidemiology and risk-factor architecture compared with other digestive neoplasms [69]. Additionally, some prior analyses grouped different drug classes together in the same exposure category. For example, Hajishah and colleagues examined gastric tumor outcomes associated with SGLT2i/DPP4i exposure but did not find conclusive results, likely reflecting limited trial counts and heterogeneity introduced by combining pharmacologically distinct regimens [10]. Similarly, Figlioli and colleagues reported no statistically significant association between GLP1RAs and gastric tumors in a subgroup analysis based on only 24 RCTs [11]. Beyond sample size, methodological choices—such as broad grouping of regimens or limited exploration of exposure duration and age—may reduce sensitivity for identifying class-level signals when event rates are low.

The present findings suggest a comparatively favorable profile for GLP1RAs and an unfavorable profile for DPP4is with respect to gastric tumor risk. While our study was not designed to establish causality, available biological literature offers plausible pathways that merit focused investigation. In a report by Yang and colleagues, DPP4 inhibition was linked to enhanced signaling through the CXCL12/CXCR4/mTOR axis [70], a pathway implicated in gastric tumor biology [71]. In contrast, GLP-1 receptors have been identified in human gastric mucosa [72], supporting the possibility that GLP1RAs may exert local effects beyond glucose regulation. Experimental work suggests that GLP-1 pathway stimulation can confer gastroprotective effects in animal models [73], including enhanced gastric mucus production [74] and reduced gastric acid secretion [75]. Nevertheless, direct human mechanistic evidence connecting these pathways to incident gastric tumor outcomes remains limited, and observational confounding cannot be excluded [76]. Therefore, carefully designed mechanistic studies—paired with improved tumor adjudication in future RCTs—are needed to test whether the observed associations represent causal effects, context-specific interactions, or chance findings in a sparse-event setting.

### Strengths and Limitations

This NMA has several strengths. First, by synthesizing evidence from 52 RCTs and 171,165 participants, we addressed a clinically consequential safety question that individual trials are seldom powered to resolve. Second, restricting inclusion to adults without baseline gastric tumors and focusing on incident events improves interpretability for primary prevention and reduces bias introduced by pre-existing disease. Third, prespecified subgroup analyses (diabetes mellitus status, study duration, and age) enhance clinical utility because real-world treatment decisions are often shaped by patient risk profiles and anticipated exposure duration. Fourth, methodological rigor was reinforced through protocol registration, PRISMA-NMA reporting, RoB 2.0 assessment, GRADE certainty ratings, and complementary frequentist and Bayesian modeling—an approach that strengthens transparency, reproducibility, and robustness, especially under rare-event conditions. Finally, the class-level estimates (GLP1RA: RR = 0.51, 95%CIs = 0.28 to 0.92; DPP4i: RR = 1.77, 95%CIs = 1.09 to 2.85) provide clinically interpretable signals that justify both clinical awareness and further confirmatory research.

Several limitations deserve attention. Evidence for newer or emerging agents (e.g., omarigliptin, teneligliptin, orforglipron, retatrutide, imeglimin, dorzagliatin, and teplizumab) was insufficient for inclusion, limiting generalizability to those regimens. Many included trials were optimized for metabolic and cardiovascular endpoints; systematic gastric tumor screening, centralized adjudication, intense surveillance, and standardized ascertainment were uncommon. This issue would be important because several important risk factors, such as smoking prevalence, obesity severity, gastroesophageal reflux, and metabolic comorbidity burden, would be tightly linked to gastric cancer risk. Although the statistical inconsistency test and heterogeneity test revealed insignificance, those clinical variables might still pose potential confounding effects on the main result of our NMA. Further, the sparse events of gastric tumors across the recruited RCTs would pose a potential risk of possible influence of a few large, long trials on DPP4i estimates. Accordingly, outcome misclassification and between-trial variability in event capture are possible, particularly where gastric tumors were recorded as adverse-event codes rather than prespecified trial endpoints. Besides, the mean follow-up of 141.8 weeks (around 2.7 years) might be insufficiently long for carcinogenesis inference. Although we arranged subgroup analysis with a cut-off point of 52 weeks of exposure time, the “52 weeks” were still insufficiently long regarding tumor formation. In addition, although we explored heterogeneity, inconsistency, and small-study effects, residual uncertainty is unavoidable when event rates are low, and statistically significant signals should be interpreted with appropriate clinical restraint. Finally, the indistinguishability of benign/malignant tumors might limit the clinical interpretation of the result of the current NMA. Despite our efforts to classify the benign versus malignant outcomes, the number of RCTs clearly reporting gastric benign/malignant tumor outcomes was too sparse to conduct network analysis.

## 4. Materials and Methods

### 4.1. Study Design and Protocol Registration

We conducted a prespecified, hypothesis-driven network meta-analysis focused on incident gastric tumor outcomes potentially associated with newer glucose-lowering therapies, following Cochrane guidance for evaluating harms in randomized trials [77]. Reporting adhered to the PRISMA extension for network meta-analyses (PRISMA-NMA; Table S1) [78]. The protocol was registered prospectively in PROSPERO (CRD420261282728) and approved by the Institutional Review Board of Tri-Service General Hospital, National Defense Medical Center, Taipei, Taiwan (TSGHIRB E202516007).

### 4.2. Literature Search and Study Identification

We implemented a comprehensive search across eight sources—PubMed, Embase, ClinicalKey, Cochrane CENTRAL, ProQuest, ScienceDirect, Web of Science, and ClinicalTrials.gov—from inception to 10 January 2026 (Table S2). Search strategies combined controlled vocabulary and free-text terms for each drug class and individual agents, with terms capturing gastric tumor outcomes and relevant adverse-event categories. Two reviewers (PT Tseng and YW Chen) independently screened titles and abstracts and then assessed full texts for eligibility. Disagreements were resolved through discussion, with third-reviewer arbitration when needed. We also hand-searched reference lists of relevant systematic reviews and meta-analyses to identify additional eligible trials. No restrictions were applied by language, publication status, or geographic setting.

### 4.3. Eligibility Criteria

Eligibility criteria were prespecified using a PICOS framework.

(a) Population: Adults without active or prior gastric tumors at baseline.
(b) Intervention: Any targeted glucose-lowering agents listed below.
(c) Comparison: Placebo, standard-of-care background therapy, or another active antidiabetic agent.
(d) Outcomes: Incident gastric tumor events defined in trial reports or coded adverse-event datasets.
(e) Study design**:** RCTs (parallel-group or factorial).

Regimens of interest included DPP4is, GLP1RAs, SGLT2is, dual GIP/GLP-1 receptor agonists (e.g., tirzepatide), triple GIP/GLP-1/glucagon receptor agonists (e.g., retatrutide), mitochondrial bioenergetics modulators (e.g., imeglimin), glucokinase activators (e.g., dorzagliatin), amylin analogues (e.g., petrelintide), and anti-CD3 monoclonal antibodies (e.g., teplizumab). Trials were eligible if they (a) enrolled adults without documented baseline gastric tumors, (b) evaluated at least one prespecified agent, (c) systematically collected and reported adverse events including gastric tumors, and (d) maintained structured safety surveillance throughout follow-up [79]. We excluded (a) non-randomized studies; (b) RCTs restricted to participants with pre-existing gastric tumors; (c) trials lacking an appropriate comparator group; (d) RCTs that did not report any gastric tumor outcomes; (e) pediatric-only trials; (f) animal or preclinical investigations; and (g) trials with clearly compromised randomization procedures or major baseline imbalances in core characteristics (e.g., age, sex, comorbidities, or concomitant therapies).

### 4.4. Risk of Bias and Quality Assessment

Two reviewers independently assessed each included RCT using the Cochrane Risk of Bias 2.0 tool [80]. We evaluated bias arising from the randomization process, deviations from intended interventions, missing outcome data, outcome measurement, and selective reporting. Each domain—and the overall trial—was classified as low risk, some concerns, or high risk. Discrepancies were resolved by consensus, with third-reviewer input when necessary.

### 4.5. Outcome Definitions and Subgroup Analyses

The primary endpoint was incident gastric tumors, defined as the first occurrence of any gastric tumor event reported during trial follow-up. We acknowledge that histopathologic terminology, diagnostic thresholds for early lesions (e.g., high-grade dysplasia vs intramucosal carcinoma) [81], and coding/reporting practices for tumors with malignant potential (e.g., GIST [82,83] and neuroendocrine tumors [84,85]) have evolved across countries and decades. Because many eligible trials reported gastric events only as ‘gastric tumor/neoplasm’ without consistent adjudication or benign/malignant labeling, we did not impose restrictions on diagnostic criteria and analyzed overall gastric neoplasms as a composite endpoint to reduce differential misclassification across the network. When statistically meaningful associations were observed at the class or regimen levels, we performed dose-stratified analyses based on protocol-defined dosage categories (Table S3).

As a secondary outcome, we investigated the risk of *Helicobacter pylori* infection, which was one of the most important risk factors for gastric tumors. Further, for a patient-relevant measure of treatment acceptability, we assessed all-cause discontinuation (drop-out rate). This choice was prespecified and aligned with our prior large-scale NMA work in related therapeutic domains [4,12,13]. When statistically meaningful signals emerged for the primary or secondary outcomes, we conducted prespecified subgroup analyses to test consistency in groups most directly exposed in routine care: (a) RCTs enrolling participants with type 2 diabetes mellitus (the principal indication for these agents), obesity, or renal insufficiency, (b) trials with study duration stratified as ≥52 weeks versus <52 weeks, and (c) trials stratified by mean participant age as ≥60 years versus <60 years. We chose 60 years as the cut-off point of age based on the previous study [86], in which the authors reported a significantly worse survival rate of gastric tumor in subjects as ≥60 years.

### 4.6. Data Extraction and Management

Study selection proceeded in two stages: title/abstract screening followed by full-text confirmation. Two authors (PT Tseng and YW Chen) independently extracted data using a standardized form capturing trial design, follow-up duration, sample size, setting, participant characteristics, eligibility criteria, treatment regimens, comparator types, target outcomes, and drop-out rates. When outcome reporting was incomplete or unclear, we contacted corresponding authors up to two times. Data handling followed the Cochrane Handbook and related methodological standards to maximize transparency, auditability, and reproducibility [87].

### 4.7. Statistical Analysis

For dichotomous outcomes, we calculated risk ratios (RRs) with 95% confidence intervals (95%CIs). Primary analyses used a random-effects NMA framework implemented with the “network” suite in STATA 16.0 (StataCorp, College Station, TX, USA) [88]. These contrast-based models integrate direct and indirect comparisons across the evidence network to estimate relative effects among all included regimens [89]. To summarize comparative rankings, we calculated the surface under the cumulative ranking curve (SUCRA), where higher values reflect a greater probability of being among the most favorable options [90]. Between-study heterogeneity was summarized using τ^2^. We assessed network consistency using complementary approaches, including loop-specific methods, node-splitting, and design-by-treatment interaction models [91]. Certainty of evidence was evaluated using GRADE adapted for NMA, incorporating considerations of study limitations, inconsistency, indirectness, imprecision, and publication bias [92]. Small-study effects and publication bias were explored using comparison-adjusted funnel plots and Egger’s regression tests.

Because gastric tumor events are uncommon in RCTs, sparse-event bias and zero-event cells are plausible. Therefore, we also conducted Bayesian NMA using the “multinma” package in R as a sensitivity analysis [93]. This approach is well-suited to rare outcomes, can reduce reliance on arbitrary continuity corrections in zero-event settings, and offers an additional robustness check under alternative modeling assumptions.

## 5. Conclusions

In this large-scale NMA of 52 RCTs including 171,165 participants, DPP4is were consistently associated with a higher risk of gastric tumor compared with controls in the overall network and in key prespecified subgroups—participants with diabetes mellitus, longer study duration (≥52 weeks), and older age (≥60 years). Conversely, GLP1RAs demonstrated a potentially protective association against gastric tumors, particularly among participants with diabetes mellitus and in longer-duration trials (≥52 weeks). In younger populations (<60 years) and shorter trials (<52 weeks), we did not identify statistically significant differences in gastric tumor risk across investigated regimens, which may partially reassure short-term prescribing in selected patients. In brief, this signal provided us insight into special attention when prescribing long-term (≥52 weeks) DPP4is in elderly subjects (≥60 years) with diabetes mellitus. The regular gastric tumor screen would be considered. These results support heightened clinical vigilance for long-term DPP4i use in higher-risk populations and underscore the need for future RCTs with improved tumor ascertainment and mechanistic studies to clarify causality.

## Figures and Tables

**Table 1:** League table of the primary outcome: gastric tumor risk (benign or malignant)—class level.

**GLP1RA**	* 1.96 (1.08, 3.55)	* 2.05 (1.02, 4.12)	**2.59 (0.37, 17.99)**	* 3.46 (1.64, 7.33)	* 6.69 (1.00, 44.90)
* 0.51 (0.28, 0.92)	Placebo_or_Control	1.05 (0.73, 1.51)	1.32 (0.21, 8.36)	* 1.77 (1.09, 2.85)	3.41 (0.56, 20.88)
* 0.49 (0.24, 0.98)	0.96 (0.66, 1.38)	SGLT2i	1.26 (0.19, 8.28)	1.69 (0.93, 3.05)	3.26 (0.53, 19.92)
0.39 (0.06, 2.68)	0.76 (0.12, 4.79)	0.79 (0.12, 5.19)	DualRA	1.34 (0.20, 8.99)	2.58 (0.19, 34.26)
* 0.29 (0.14, 0.61)	* 0.57 (0.35, 0.92)	0.59 (0.33, 1.07)	0.75 (0.11, 5.03)	DPP4i	1.93 (0.32, 11.80)
0.15 (0.02, 1.00)	0.29 (0.05, 1.80)	0.31 (0.05, 1.88)	0.39 (0.03, 5.14)	0.52 (0.08, 3.17)	SGLT2i_DPP4i

Data presents RR [95%CIs]. Network meta-analysis results are presented as estimated effect sizes for the outcome of gastric tumor risk. Interventions are reported in order of mean ranking of beneficial prophylactic effect on gastric tumor risk, and outcomes are expressed as risk ratio (RR) (95% confidence intervals) (95%CIs). For the upper-right portion, RR of less than 1 indicates that the treatment specified in the row got more beneficial effect than that specified in the column. For the lower-left portion, RR of less than 1 indicates that the treatment specified in the column has a more beneficial effect than that specified in the row. Bold results marked with * indicate statistical significance.

**Table 2:** League table of the primary outcome: gastric tumor risk (benign or malignant)—regimen level.

Oral_semaglutide	1.25 (0.13, 12.04)	1.42 (0.17, 12.03)	1.79 (0.16, 19.60)	2.54 (0.31, 20.72)	2.80 (0.40, 19.48)	2.68 (0.14, 50.30)	2.79 (0.38, 20.64)	2.78 (0.17, 45.05)	2.80 (0.18, 44.09)	2.81 (0.10, 82.68)	3.11 (0.25, 38.41)	3.25 (0.39, 27.12)	4.23 (0.09, 198.00)	3.70 (0.25, 53.80)	4.91 (0.68, 35.57)	5.14 (0.64, 41.24)	8.77 (0.33, 236.22)	7.63 (0.30, 193.89)	8.14 (0.57, 115.21)	29.42 (0.95, 913.73)
0.80 (0.08, 7.75)	Liraglutide	1.14 (0.26, 5.02)	1.44 (0.23, 8.96)	2.04 (0.49, 8.52)	2.25 (0.69, 7.29)	2.15 (0.18, 26.06)	2.24 (0.62, 8.03)	2.23 (0.22, 22.72)	2.25 (0.23, 22.09)	2.25 (0.11, 45.75)	2.49 (0.34, 18.18)	2.61 (0.60, 11.24)	3.40 (0.10, 115.22)	2.97 (0.33, 26.49)	3.94 (0.97, 15.95)	* 4.12 (1.01, 16.75)	7.03 (0.38, 129.26)	6.12 (0.36, 105.40)	6.52 (0.75, 56.41)	* 23.60 (1.09, 508.91)
0.70 (0.08, 5.96)	0.88 (0.20, 3.87)	Exenatide	1.26 (0.24, 6.68)	1.79 (0.53, 6.03)	1.97 (0.80, 4.87)	1.89 (0.18, 20.35)	1.96 (0.70, 5.50)	1.96 (0.24, 15.63)	1.97 (0.23, 17.06)	1.98 (0.11, 36.48)	2.19 (0.35, 13.75)	2.29 (0.65, 8.00)	2.98 (0.12, 72.90)	2.61 (0.33, 20.33)	* 3.46 (1.08, 11.11)	* 3.62 (1.11, 11.79)	6.17 (0.37, 102.71)	5.37 (0.35, 83.55)	5.73 (0.76, 43.20)	* 20.71 (1.06, 406.56)
0.56 (0.05, 6.10)	0.70 (0.11, 4.33)	0.79 (0.15, 4.19)	Inject_semaglutide	1.42 (0.28, 7.16)	1.56 (0.39, 6.35)	1.49 (0.11, 20.29)	1.56 (0.35, 6.88)	1.55 (0.13, 17.83)	1.56 (0.14, 17.37)	1.57 (0.07, 34.96)	1.73 (0.21, 14.55)	1.81 (0.35, 9.41)	2.36 (0.06, 86.88)	2.07 (0.20, 20.94)	2.74 (0.56, 13.45)	2.86 (0.58, 14.12)	4.89 (0.24, 99.08)	4.26 (0.22, 80.97)	4.54 (0.46, 44.66)	16.41 (0.69, 388.20)
0.39 (0.05, 3.21)	0.49 (0.12, 2.05)	0.56 (0.17, 1.89)	0.71 (0.14, 3.57)	Empagliflozin	1.10 (0.49, 2.49)	1.06 (0.10, 11.02)	1.10 (0.42, 2.85)	1.09 (0.13, 9.49)	1.10 (0.13, 9.20)	1.11 (0.06, 19.87)	1.22 (0.20, 7.37)	1.28 (0.39, 4.20)	1.67 (0.05, 50.97)	1.46 (0.19, 10.95)	1.94 (0.65, 5.76)	2.02 (0.68, 6.03)	3.45 (0.21, 55.90)	3.01 (0.23, 39.91)	3.20 (0.44, 23.26)	11.58 (0.61, 221.59)
0.36 (0.05, 2.48)	0.44 (0.14, 1.44)	0.51 (0.21, 1.25)	0.64 (0.16, 2.60)	0.91 (0.40, 2.05)	Placebo_or_Control	0.96 (0.11, 8.63)	1.00 (0.61, 1.63)	0.99 (0.13, 7.33)	1.00 (0.14, 7.09)	1.00 (0.06, 16.01)	1.11 (0.22, 5.50)	1.16 (0.49, 2.76)	1.51 (0.05, 41.86)	1.32 (0.21, 8.36)	1.75 (0.82, 3.73)	1.83 (0.85, 3.93)	3.13 (0.22, 44.84)	2.72 (0.20, 36.34)	2.90 (0.48, 17.69)	10.50 (0.62, 179.05)
0.37 (0.02, 7.00)	0.47 (0.04, 5.64)	0.53 (0.05, 5.71)	0.67 (0.05, 9.08)	0.95 (0.09, 9.89)	1.05 (0.12, 9.43)	Bexagliflozin	1.04 (0.11, 9.93)	1.04 (0.05, 20.28)	1.04 (0.05, 19.88)	1.05 (0.03, 36.06)	1.16 (0.08, 17.61)	1.21 (0.11, 12.90)	1.58 (0.03, 84.88)	1.38 (0.08, 24.38)	1.83 (0.18, 18.76)	1.92 (0.19, 19.66)	3.27 (0.10, 103.42)	2.85 (0.10, 85.22)	3.03 (0.18, 52.31)	10.98 (0.30, 397.52)
0.36 (0.05, 2.65)	0.45 (0.12, 1.60)	0.51 (0.18, 1.43)	0.64 (0.15, 2.84)	0.91 (0.35, 2.36)	1.00 (0.61, 1.65)	0.96 (0.10, 9.16)	Dapagliflozin	1.00 (0.13, 7.83)	1.00 (0.13, 7.57)	1.01 (0.06, 16.81)	1.11 (0.21, 5.81)	1.16 (0.43, 3.16)	1.52 (0.05, 43.62)	1.33 (0.20, 8.96)	1.76 (0.71, 4.34)	1.84 (0.74, 4.57)	3.14 (0.22, 44.69)	2.73 (0.20, 38.25)	2.91 (0.45, 19.00)	10.54 (0.59, 187.73)
0.36 (0.02, 5.84)	0.45 (0.04, 4.58)	0.51 (0.06, 4.09)	0.65 (0.06, 7.43)	0.91 (0.11, 7.93)	1.01 (0.14, 7.47)	0.96 (0.05, 18.88)	1.00 (0.13, 7.90)	Dulaglutide	1.01 (0.06, 16.59)	1.01 (0.03, 30.87)	1.12 (0.09, 14.52)	1.17 (0.13, 10.36)	1.52 (0.03, 69.03)	1.33 (0.09, 20.28)	1.77 (0.21, 14.99)	1.85 (0.22, 15.74)	3.16 (0.11, 88.27)	2.75 (0.10, 72.59)	2.93 (0.20, 43.44)	10.59 (0.33, 340.92)
0.36 (0.02, 5.62)	0.45 (0.05, 4.38)	0.51 (0.06, 4.39)	0.64 (0.06, 7.12)	0.91 (0.11, 7.57)	1.00 (0.14, 7.10)	0.96 (0.05, 18.21)	1.00 (0.13, 7.52)	0.99 (0.06, 16.33)	Sotagliflozin	1.00 (0.03, 29.89)	1.11 (0.09, 13.94)	1.16 (0.14, 9.89)	1.51 (0.03, 71.54)	1.32 (0.09, 19.51)	1.76 (0.21, 14.33)	1.83 (0.22, 15.02)	3.13 (0.11, 85.41)	2.73 (0.11, 70.20)	2.91 (0.20, 41.78)	10.51 (0.33, 330.19)
0.36 (0.01, 10.46)	0.44 (0.02, 9.00)	0.51 (0.03, 9.31)	0.64 (0.03, 14.22)	0.90 (0.05, 16.22)	1.00 (0.06, 15.92)	0.95 (0.03, 32.78)	0.99 (0.06, 16.56)	0.99 (0.03, 30.14)	1.00 (0.03, 29.65)	Vildagliptin	1.11 (0.05, 27.12)	1.16 (0.06, 21.07)	1.51 (0.02, 113.87)	1.32 (0.05, 36.74)	1.75 (0.10, 30.87)	1.83 (0.10, 32.33)	3.12 (0.07, 145.46)	2.71 (0.06, 120.55)	2.89 (0.11, 79.08)	10.46 (0.20, 551.75)
0.32 (0.03, 3.97)	0.40 (0.05, 2.92)	0.46 (0.07, 2.87)	0.58 (0.07, 4.83)	0.82 (0.14, 4.92)	0.90 (0.18, 4.46)	0.86 (0.06, 13.08)	0.90 (0.17, 4.67)	0.89 (0.07, 11.58)	0.90 (0.07, 11.30)	0.90 (0.04, 22.16)	Saxagliptin	1.04 (0.17, 6.45)	1.36 (0.03, 54.35)	1.19 (0.10, 13.68)	1.58 (0.27, 9.26)	1.65 (0.28, 9.72)	2.82 (0.20, 40.10)	2.45 (0.12, 51.57)	2.62 (0.23, 29.23)	9.46 (0.36, 245.59)
0.31 (0.04, 2.57)	0.38 (0.09, 1.66)	0.44 (0.13, 1.53)	0.55 (0.11, 2.87)	0.78 (0.24, 2.57)	0.86 (0.36, 2.05)	0.82 (0.08, 8.77)	0.86 (0.32, 2.33)	0.85 (0.10, 7.57)	0.86 (0.10, 7.34)	0.87 (0.05, 15.77)	0.96 (0.16, 5.91)	Canagliflozin	1.30 (0.04, 40.35)	1.14 (0.15, 8.75)	1.51 (0.48, 4.73)	1.58 (0.50, 5.02)	2.70 (0.16, 44.38)	2.35 (0.15, 36.09)	2.50 (0.34, 18.58)	9.05 (0.47, 175.80)
0.24 (0.01, 11.04)	0.29 (0.01, 9.99)	0.34 (0.01, 8.20)	0.42 (0.01, 15.58)	0.60 (0.02, 18.34)	0.66 (0.02, 18.35)	0.63 (0.01, 34.02)	0.66 (0.02, 18.95)	0.66 (0.01, 29.72)	0.66 (0.01, 31.29)	0.66 (0.01, 50.20)	0.73 (0.02, 29.33)	0.77 (0.02, 23.77)	Lixisenatide	0.87 (0.02, 39.09)	1.16 (0.04, 34.88)	1.21 (0.04, 36.65)	2.07 (0.03, 146.23)	1.80 (0.03, 121.77)	1.92 (0.04, 84.34)	6.95 (0.09, 548.27)
0.27 (0.02, 3.92)	0.34 (0.04, 3.00)	0.38 (0.05, 2.99)	0.48 (0.05, 4.91)	0.69 (0.09, 5.15)	0.76 (0.12, 4.79)	0.72 (0.04, 12.78)	0.75 (0.11, 5.09)	0.75 (0.05, 11.41)	0.76 (0.05, 11.15)	0.76 (0.03, 21.17)	0.84 (0.07, 9.65)	0.88 (0.11, 6.74)	1.14 (0.03, 51.09)	Tirzepatide	1.33 (0.18, 9.74)	1.39 (0.19, 10.21)	2.37 (0.09, 60.40)	2.06 (0.09, 49.59)	2.20 (0.17, 29.06)	7.94 (0.27, 234.18)
0.20 (0.03, 1.47)	0.25 (0.06, 1.03)	* 0.29 (0.09, 0.93)	0.36 (0.07, 1.79)	0.52 (0.17, 1.54)	0.57 (0.27, 1.21)	0.55 (0.05, 5.58)	0.57 (0.23, 1.40)	0.57 (0.07, 4.79)	0.57 (0.07, 4.65)	0.57 (0.03, 10.10)	0.63 (0.11, 3.71)	0.66 (0.21, 2.07)	0.86 (0.03, 25.89)	0.75 (0.10, 5.53)	Sitagliptin	1.04 (0.36, 3.05)	1.78 (0.11, 28.40)	1.55 (0.10, 22.98)	1.66 (0.23, 11.73)	5.99 (0.32, 112.69)
0.19 (0.02, 1.56)	* 0.24 (0.06, 0.99)	* 0.28 (0.08, 0.90)	0.35 (0.07, 1.72)	0.49 (0.17, 1.48)	0.55 (0.25, 1.17)	0.52 (0.05, 5.36)	0.54 (0.22, 1.35)	0.54 (0.06, 4.61)	0.55 (0.07, 4.47)	0.55 (0.03, 9.69)	0.61 (0.10, 3.56)	0.63 (0.20, 2.01)	0.82 (0.03, 24.91)	0.72 (0.10, 5.31)	0.96 (0.33, 2.80)	Linagliptin	1.71 (0.11, 27.24)	1.49 (0.11, 19.73)	1.58 (0.22, 11.27)	5.73 (0.30, 108.09)
0.11 (0.00, 3.07)	0.14 (0.01, 2.62)	0.16 (0.01, 2.70)	0.20 (0.01, 4.15)	0.29 (0.02, 4.69)	0.32 (0.02, 4.59)	0.31 (0.01, 9.67)	0.32 (0.02, 4.53)	0.32 (0.01, 8.87)	0.32 (0.01, 8.72)	0.32 (0.01, 14.97)	0.35 (0.02, 5.05)	0.37 (0.02, 6.10)	0.48 (0.01, 34.12)	0.42 (0.02, 10.78)	0.56 (0.04, 8.93)	0.59 (0.04, 9.35)	Dapagliflozin_ Saxagliptin	0.87 (0.02, 35.78)	0.93 (0.04, 23.20)	3.36 (0.07, 164.32)
0.13 (0.01, 3.33)	0.16 (0.01, 2.81)	0.19 (0.01, 2.89)	0.23 (0.01, 4.47)	0.33 (0.03, 4.42)	0.37 (0.03, 4.90)	0.35 (0.01, 10.51)	0.37 (0.03, 5.11)	0.36 (0.01, 9.62)	0.37 (0.01, 9.45)	0.37 (0.01, 16.36)	0.41 (0.02, 8.56)	0.43 (0.03, 6.54)	0.55 (0.01, 37.47)	0.49 (0.02, 11.68)	0.64 (0.04, 9.53)	0.67 (0.05, 8.94)	1.15 (0.03, 47.18)	Empagliflozin_ Linagliptin	1.07 (0.05, 25.10)	3.85 (0.08, 179.69)
0.12 (0.01, 1.74)	0.15 (0.02, 1.33)	0.17 (0.02, 1.32)	0.22 (0.02, 2.17)	0.31 (0.04, 2.27)	0.34 (0.06, 2.10)	0.33 (0.02, 5.68)	0.34 (0.05, 2.24)	0.34 (0.02, 5.07)	0.34 (0.02, 4.95)	0.35 (0.01, 9.44)	0.38 (0.03, 4.27)	0.40 (0.05, 2.97)	0.52 (0.01, 22.85)	0.46 (0.03, 6.02)	0.60 (0.09, 4.28)	0.63 (0.09, 4.49)	1.08 (0.04, 26.93)	0.94 (0.04, 22.10)	Alogliptin	3.62 (0.13, 104.50)
0.03 (0.00, 1.06)	* 0.04 (0.00, 0.91)	* 0.05 (0.00, 0.95)	0.06 (0.00, 1.44)	0.09 (0.00, 1.65)	0.10 (0.01, 1.63)	0.09 (0.00, 3.30)	0.09 (0.01, 1.69)	0.09 (0.00, 3.04)	0.10 (0.00, 2.99)	0.10 (0.00, 5.04)	0.11 (0.00, 2.75)	0.11 (0.01, 2.14)	0.14 (0.00, 11.36)	0.13 (0.00, 3.71)	0.17 (0.01, 3.14)	0.17 (0.01, 3.29)	0.30 (0.01, 14.58)	0.26 (0.01, 12.09)	0.28 (0.01, 7.99)	Ertugliflozin

Data presents RR [95%CIs]. Network meta-analysis results are presented as estimated effect sizes for the outcome of gastric tumor risk. Interventions are reported in order of mean ranking of beneficial prophylactic effect on gastric tumor risk, and outcomes are expressed as risk ratio (RR) (95% confidence intervals) (95%CIs). For the upper-right portion, RR of less than 1 indicates that the treatment specified in the row got more beneficial effect than that specified in the column. For the lower-left portion, RR of less than 1 indicates that the treatment specified in the column has a more beneficial effect than that specified in the row. Bold results marked with * indicate statistical significance.

**Table 3:** League table of the primary outcome: gastric tumor (benign or malignant) risk – focusing on participants with diabetes mellitus.

GLP1RA	1.82 (0.88, 3.77)	* 1.96 (1.08, 3.55)	2.42 (0.23, 25.11)	* 3.44 (1.63, 7.29)	6.28 (0.93, 42.32)
0.55 (0.27, 1.14)	SGLT2i	1.08 (0.71, 1.65)	1.33 (0.13, 13.29)	* 1.89 (1.01, 3.54)	3.45 (0.56, 21.15)
* 0.51 (0.28, 0.92)	0.93 (0.61, 1.42)	Placebo_or_Control	1.24 (0.13, 11.86)	* 1.76 (1.09, 2.84)	3.20 (0.52, 19.68)
0.41 (0.04, 4.27)	0.75 (0.08, 7.50)	0.81 (0.08, 7.77)	DualRA	1.42 (0.14, 14.34)	2.59 (0.14, 47.10)
* 0.29 (0.14, 0.61)	* 0.53 (0.28, 0.99)	* 0.57 (0.35, 0.92)	0.70 (0.07, 7.10)	DPP4i	1.82 (0.30, 11.18)
0.16 (0.02, 1.07)	0.29 (0.05, 1.78)	0.31 (0.05, 1.92)	0.39 (0.02, 7.02)	0.55 (0.09, 3.36)	SGLT2i_DPP4i

Data presents RR [95%CIs]. Network meta-analysis results are presented as estimated effect sizes for the outcome of gastric tumor risk. Interventions are reported in order of mean ranking of beneficial prophylactic effect on gastric tumor risk, and outcomes are expressed as risk ratio (RR) (95% confidence intervals) (95%CIs). For the upper-right portion, RR of less than 1 indicates that the treatment specified in the row got more beneficial effect than that specified in the column. For the lower-left portion, RR of less than 1 indicates that the treatment specified in the column has a more beneficial effect than that specified in the row. Bold results marked with * indicate statistical significance. Abbreviation: 95%CIs: 95% confidence intervals; DPP4i: dipeptidyl-peptidase 4 inhibitor; GLP1RA: glucagon-like peptide-1 agonist; NMA: network meta-analysis; RR: risk ratio; RCT: randomized controlled trial; SGLT2i: sodium–glucose cotransporter 2 inhibitor.

## Data Availability

These data were derived from the following resources available in the public domain: https://clinicaltrials.gov/ (access date: 10 January2026).

## Supplementary Materials

The following supporting information can be downloaded at: https://www.mdpi.com/article/10.3390/ijms27062619/s1.

## Abbreviations

95%CIs: 95% confidence intervals; DPP4 inhibitor: dipeptidyl peptidase 4 inhibitor; GLP-1 agonist: glucagon-like peptide-1 agonist; NMA: network meta-analysis; RCT: randomized controlled trial; RR: risk ratio; SGLT2 inhibitor: sodium–glucose cotransporter 2 inhibitor.
