# Supplementary material for: Gastric Neoplasm Risk with DPP-4 Inhibitors, GLP-1 Receptor Agonists, and SGLT2 Inhibitors: Network Meta-Analysis of Randomized Trials
Source: Int J Mol Sci. 2026 Mar 13;27(6):2619. doi: 10.3390/ijms27062619 (PMC13026302; doi:10.3390/ijms27062619)
Supplement: Supplementary file 1 [file ijms-27-02619-s001.zip › ijms-4126376-supplementary.pdf]

List of content in supplement materials

Gastric Neoplasms Risk With DPP-4 Inhibitors, GLP-1 Receptor Agonists, and SGLT2 Inhibitors

Network Meta-analysis of Randomized Trials

Chao-Ming Hung, et al.

Figure S1	(A) Network structure of NMA of primary outcome: gastric tumor risk – study duration at least 52 weeks
	(B) Network structure of NMA of primary outcome: gastric tumor risk – study duration less than 52 weeks
	(C) Network structure of NMA of primary outcome: gastric tumor risk – at least 60 years old
	(D) Network structure of NMA of primary outcome: gastric tumor risk – less than 60 years old
	(E) Network structure of NMA of secondary outcome: *Helicobacter pylori* risk
	(F) Network structure of NMA of acceptability: drop-out rate
	(G) Network structure of NMA of primary outcome: gastric tumor risk – dose level
Figure S2	(A) Forest plot of NMA of primary outcome: gastric tumor risk – study duration at least 52 weeks
	(B) Forest plot of NMA of primary outcome: gastric tumor risk – study duration less than 52 weeks
	(C) Forest plot of NMA of primary outcome: gastric tumor risk – at least 60 years old
	(D) Forest plot of NMA of primary outcome: gastric tumor risk – less than 60 years old
	(E) Forest plot of NMA of secondary outcome: *Helicobacter pylori* risk
	(F) Forest plot of NMA of acceptability: drop-out rate
	(G) Forest plot of NMA of primary outcome: gastric tumor risk – dose level
Figure S3	Individual study result of primary outcome: gastric tumor risk
Figure S4	Funnel plot for primary outcome: gastric tumor risk
Figure S5	Egger test for primary outcome: gastric tumor risk
Figure S6	Bayesian network meta-analysis of primary outcome: gastric tumor risk
Figure S7	Risk of bias tool 2.0
Table S1	PRISMA 2020 checklist of the current network meta-analysis
Table S2	Keyword used in each database and search results
Table S3	Dosage stratification (stratified according to the included original RCTs)
Table S4	Excluded studies and reason
Table S5	Characteristics of the included studies

Table S6	(A) League table of NMA of primary outcome: gastric tumor risk – study duration at least 52 weeks
	(B) League table of NMA of primary outcome: gastric tumor risk – study duration less than 52 weeks
	(C) League table of NMA of primary outcome: gastric tumor risk – at least 60 years old
	(D) League table of NMA of primary outcome: gastric tumor risk – less than 60 years old
	(E) League table of NMA of secondary outcome: *Helicobacter pylori* risk
	(F) League table of NMA of acceptability: drop-out rate
Table S7	(A) SUCRA of NMA of primary outcome: gastric tumor risk – class level
	(B) SUCRA of NMA of primary outcome: gastric tumor risk – regimen level
	(C) SUCRA of NMA of primary outcome: gastric tumor risk – focus participants with diabetes mellitus
	(D) SUCRA of NMA of primary outcome: gastric tumor risk – study duration at least 52 weeks
	(E) SUCRA of NMA of primary outcome: gastric tumor risk – study duration less than 52 weeks
	(F) SUCRA of NMA of primary outcome: gastric tumor risk – at least 60 years old
	(G) SUCRA of NMA of primary outcome: gastric tumor risk – less than 60 years old
	(H) SUCRA of NMA of acceptability: drop-out rate
Table S8	Heterogeneity for primary outcome: gastric tumor risk
Table S9	(A) Side-splitting model inconsistency for primary outcome: gastric tumor risk
	(B) Design-by-treatment model and loop inconsistency for primary outcome: gastric tumor risk
Table S10	GRADE for primary outcome: gastric tumor risk

Figure S1A Network structure of NMA of primary outcome: gastric tumor risk – study duration at least 52 weeks

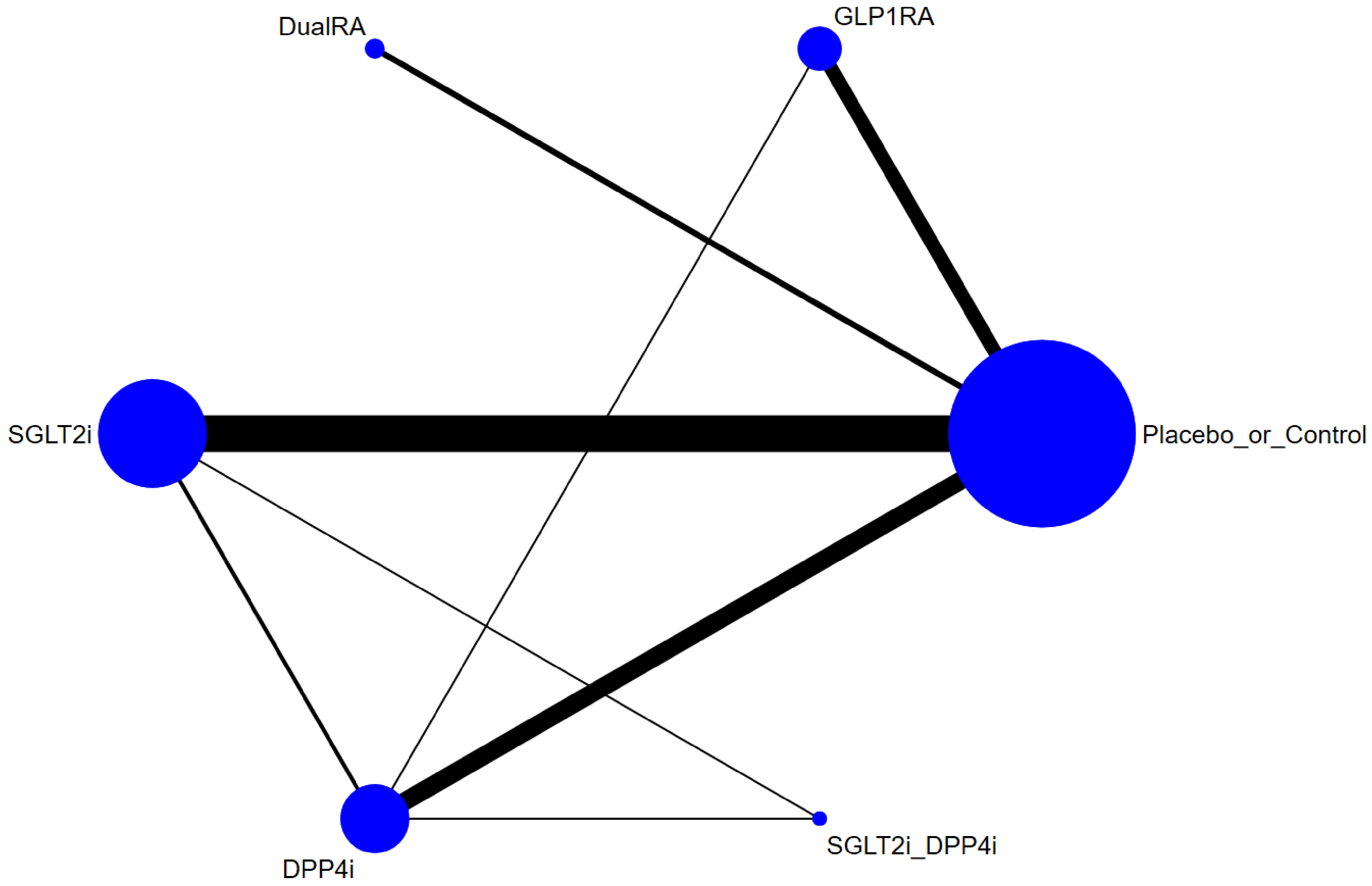

Figure S1B Network structure of NMA of primary outcome: gastric tumor risk – study duration less than 52 weeks

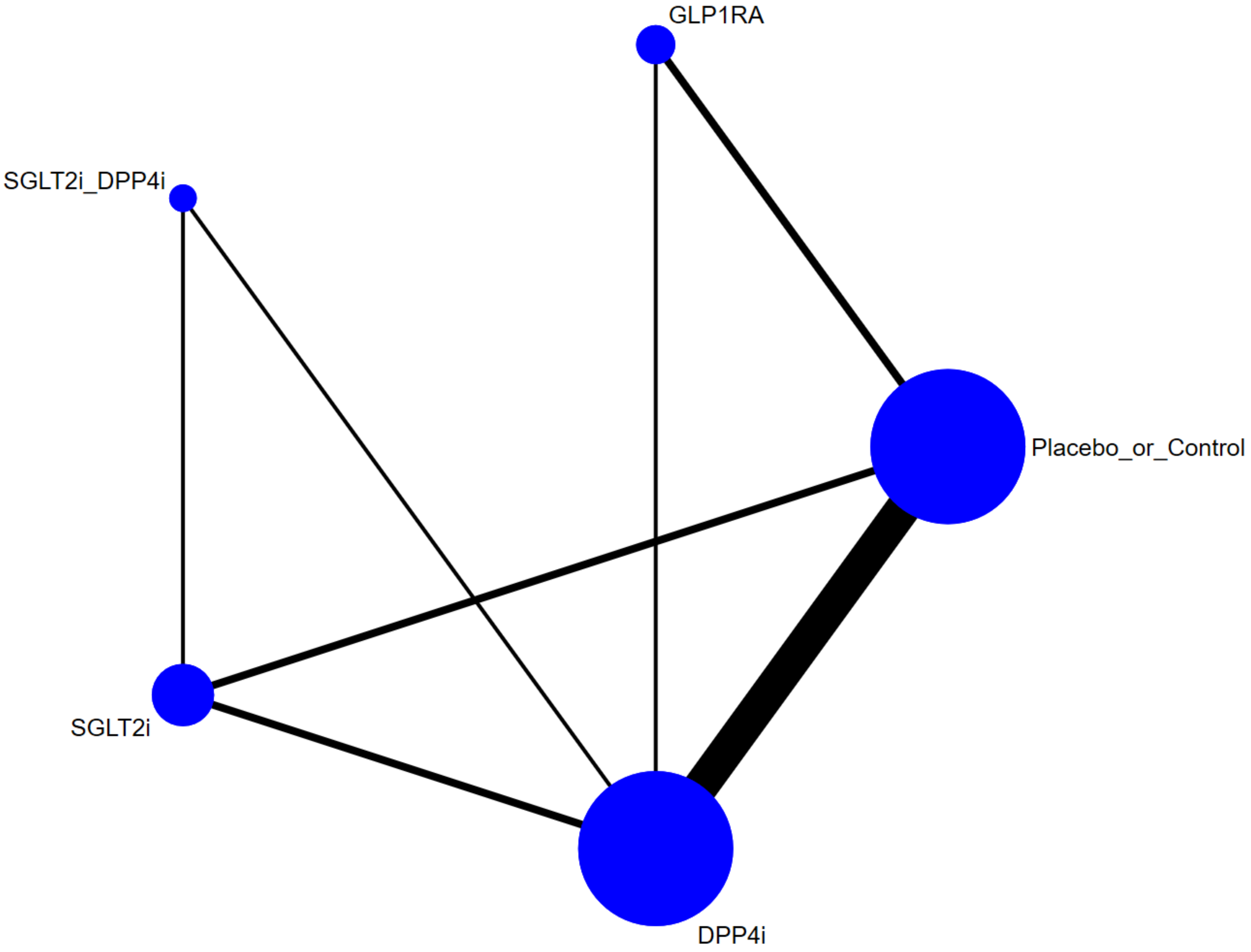

Figure S1C Network structure of NMA of primary outcome: gastric tumor risk – at least 60 years old

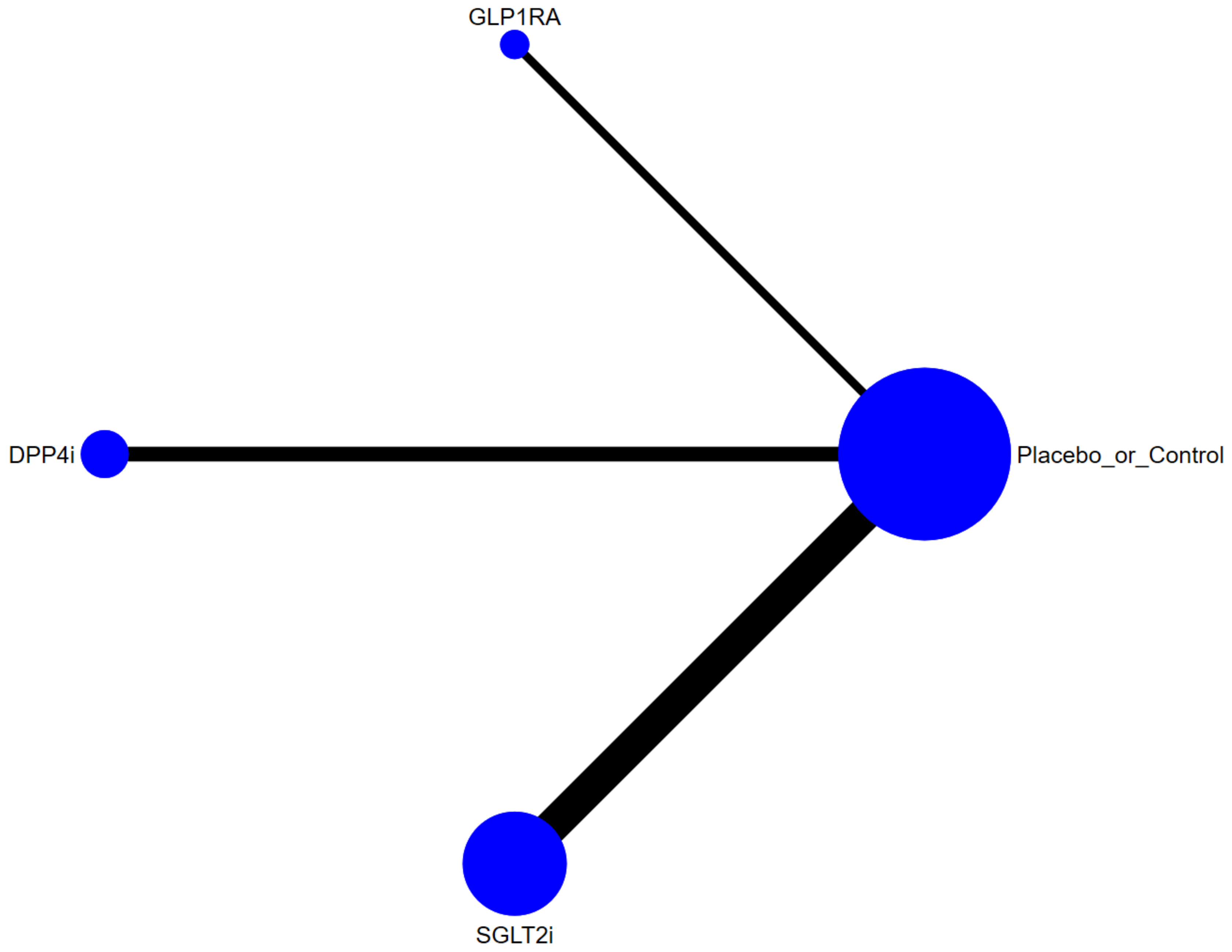

Figure S1D Network structure of NMA of primary outcome: gastric tumor risk – less than 60 years old

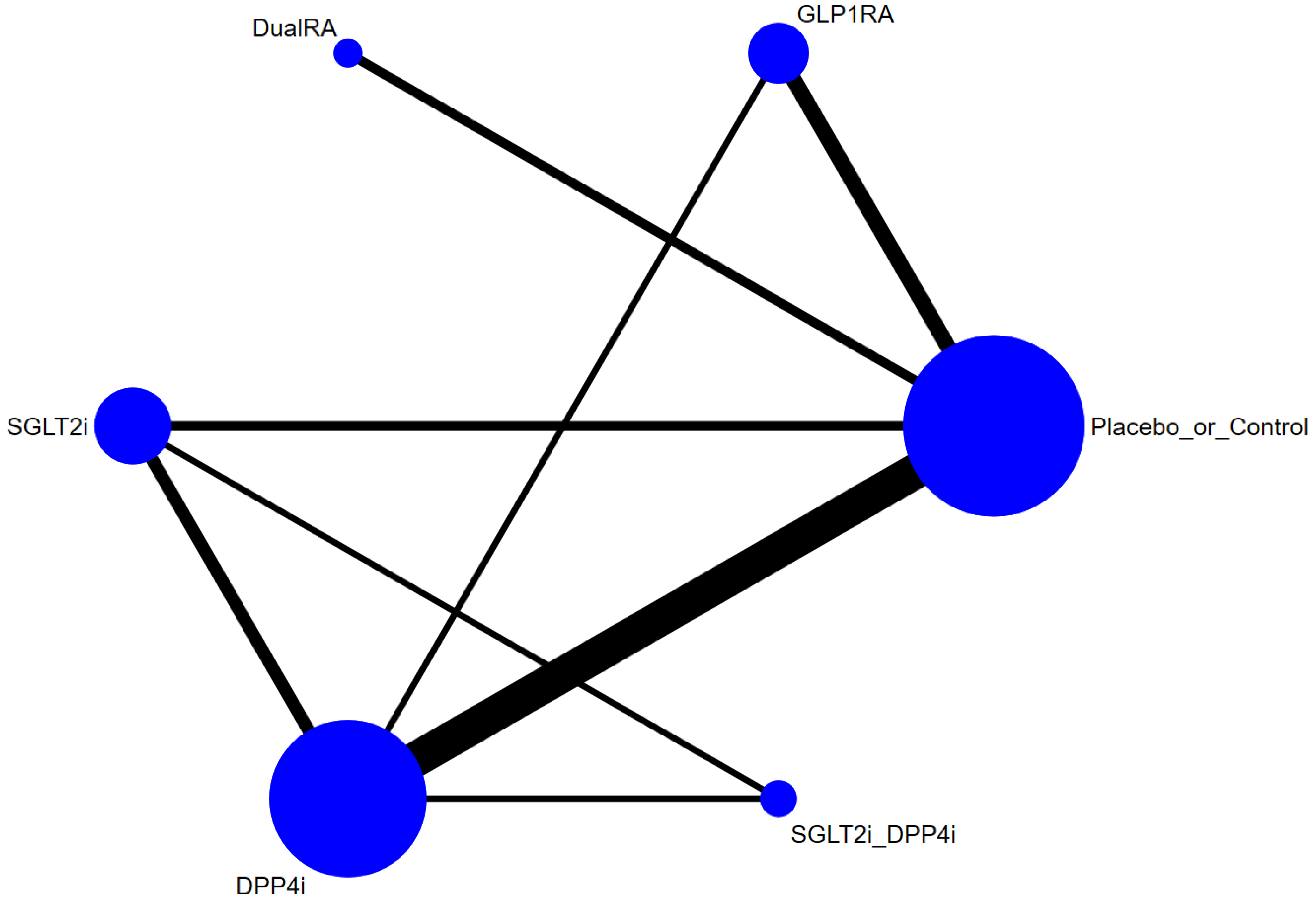

Figure S1E Network structure of NMA of secondary outcome: *Helicobacter pylori* risk

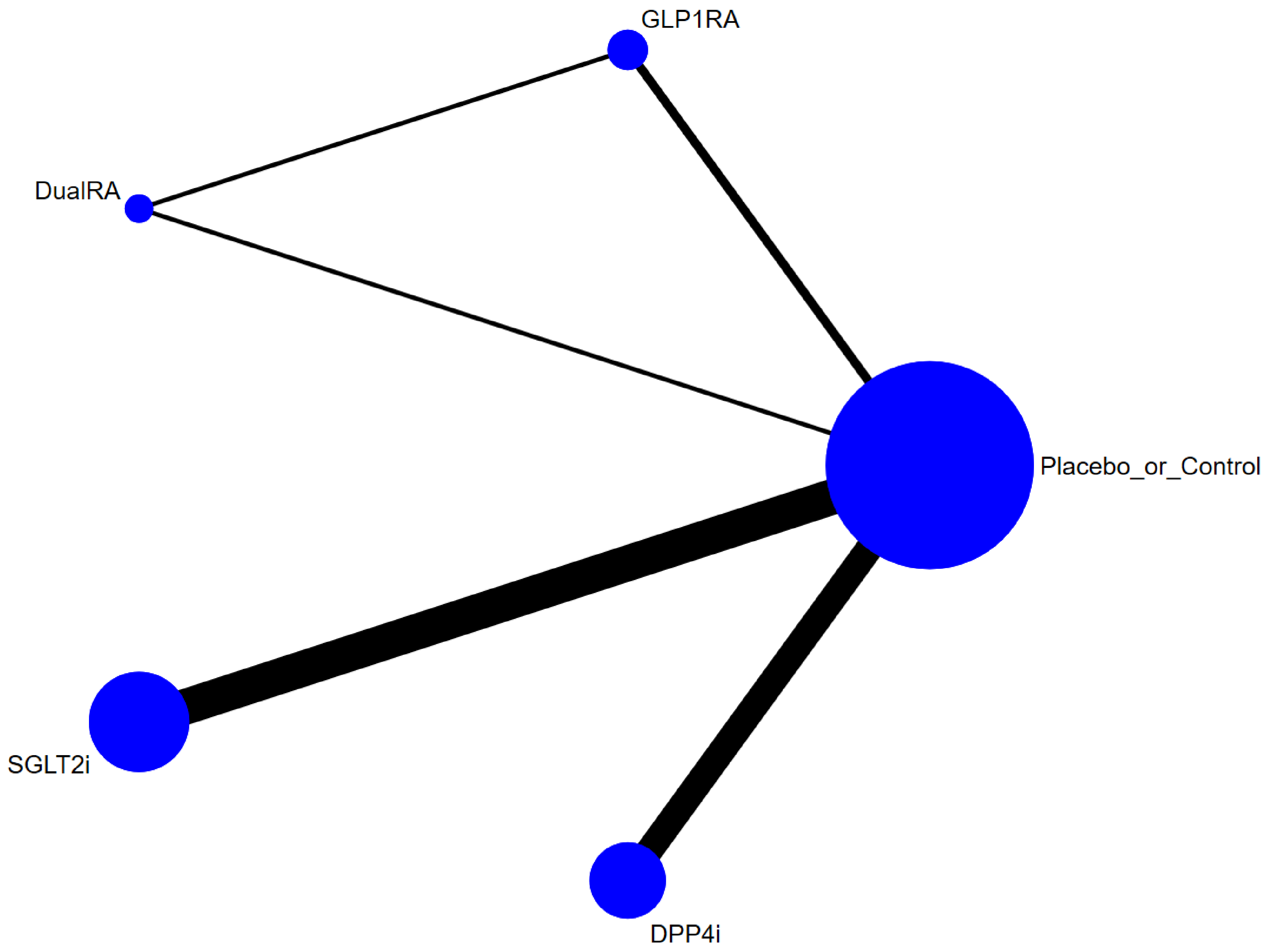

Figure S1F Network structure of NMA of acceptability: drop-out rate

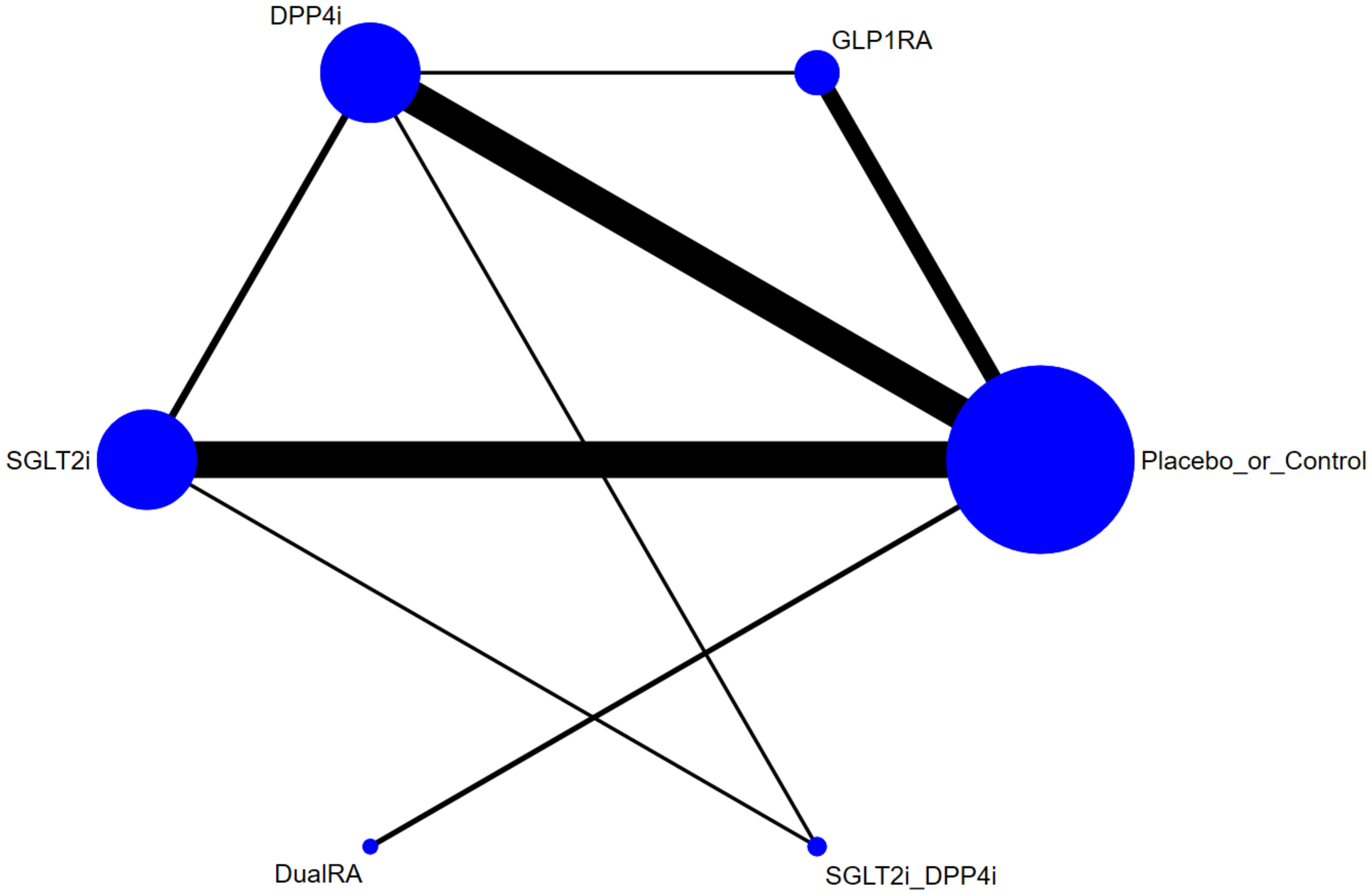

Figure S1G Network structure of NMA of primary outcome: gastric tumor risk – dose level

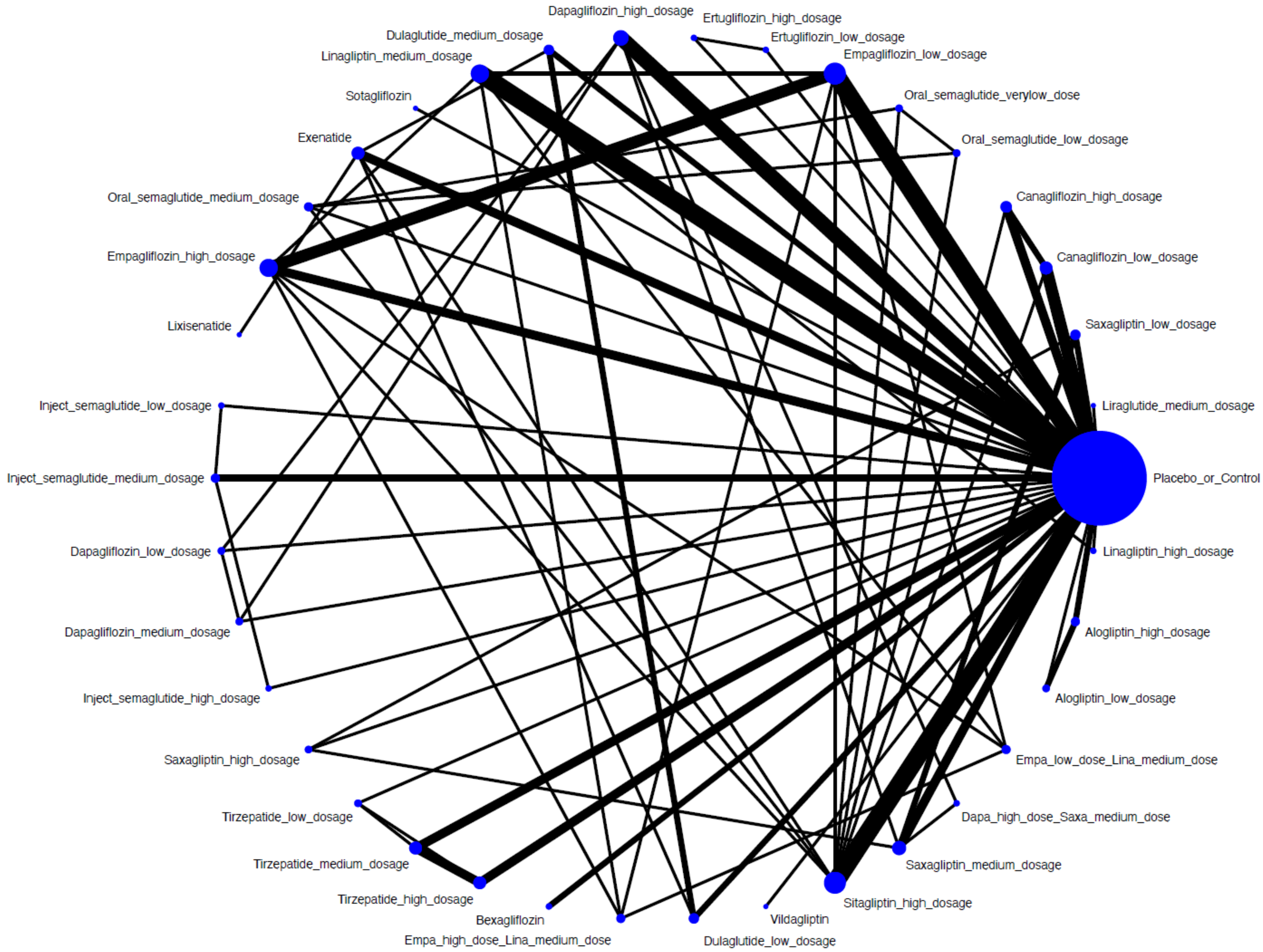

Figure legend of Figure S1A-S1G

Overall structure of the network meta-analysis. The lines between nodes represent direct comparisons in various trials, and the size of each circle is proportional to the number of participants in each specific treatment. The thickness of the lines is proportional to the number of trials connected to the network.

Abbreviation for Figure S1A-S1G:

95%CIs: 95% confidence intervals; DPP4 inhibitor: dipeptidyl peptidase 4 inhibitor; GLP-1 agonist: glucagon-like peptide-1 agonist; NMA: network meta-analysis; RCT: randomized controlled trial; RR: risk ratio; SGLT2 inhibitor: sodium–glucose cotransporter 2 inhibitor

Figure S2A Forest plot of NMA of primary outcome: gastric tumor risk – study duration at least 52 weeks

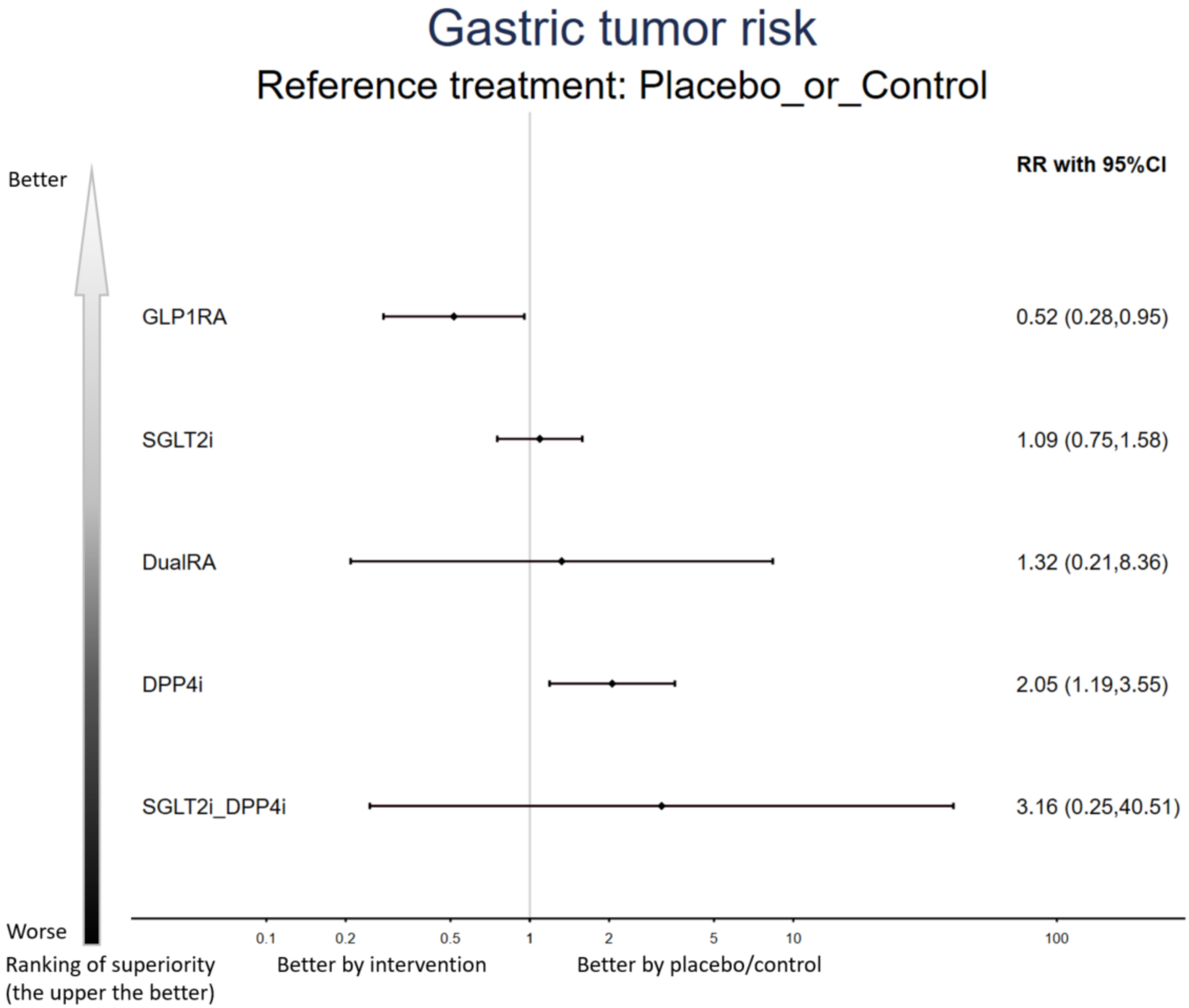

Figure S2B Forest plot of NMA of primary outcome: gastric tumor risk – study duration less than 52 weeks

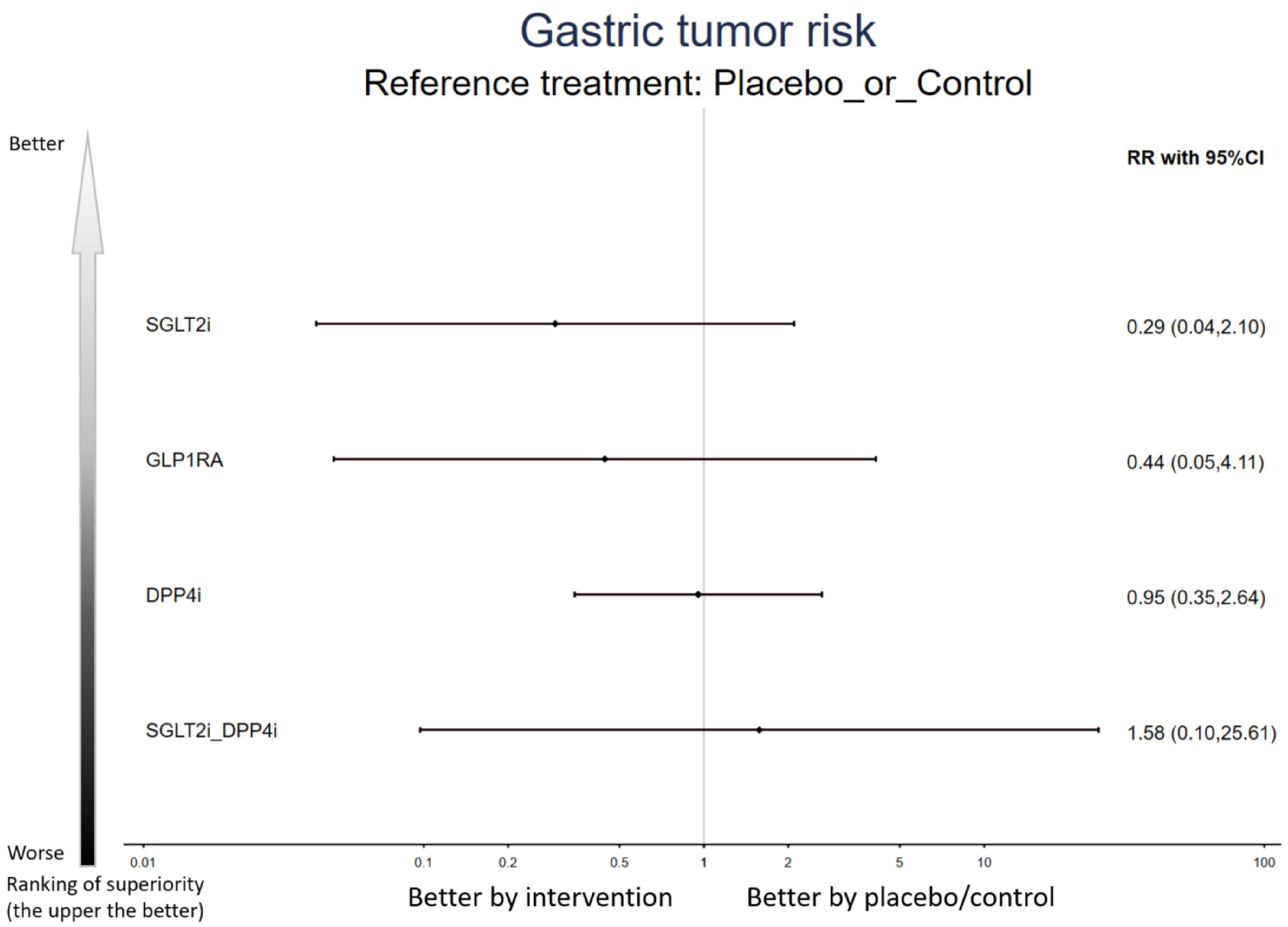

Figure S2C Forest plot of NMA of primary outcome: gastric tumor risk – at least 60 years old

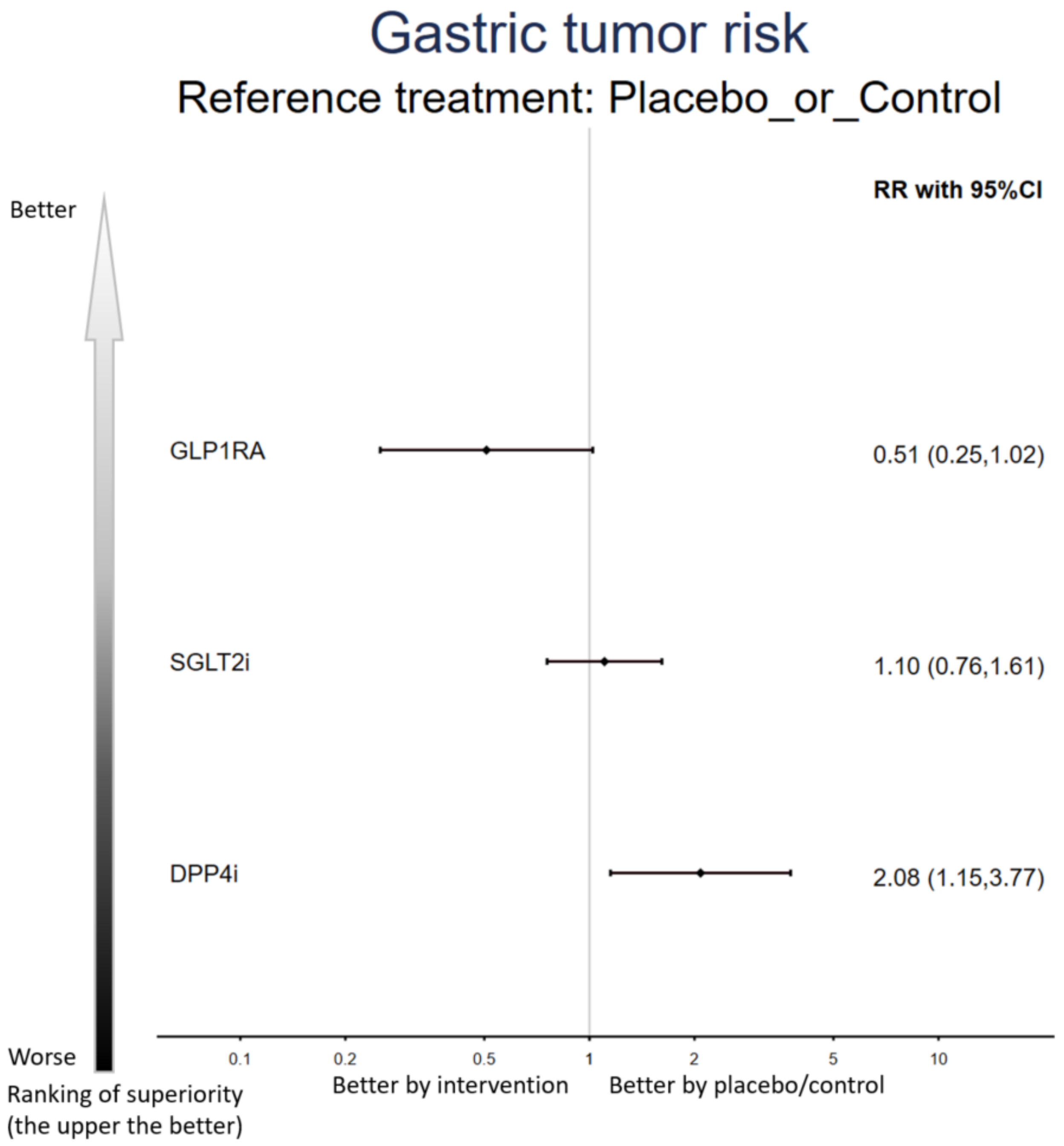

Figure S2D Forest plot of NMA of primary outcome: gastric tumor risk – less than 60 years old

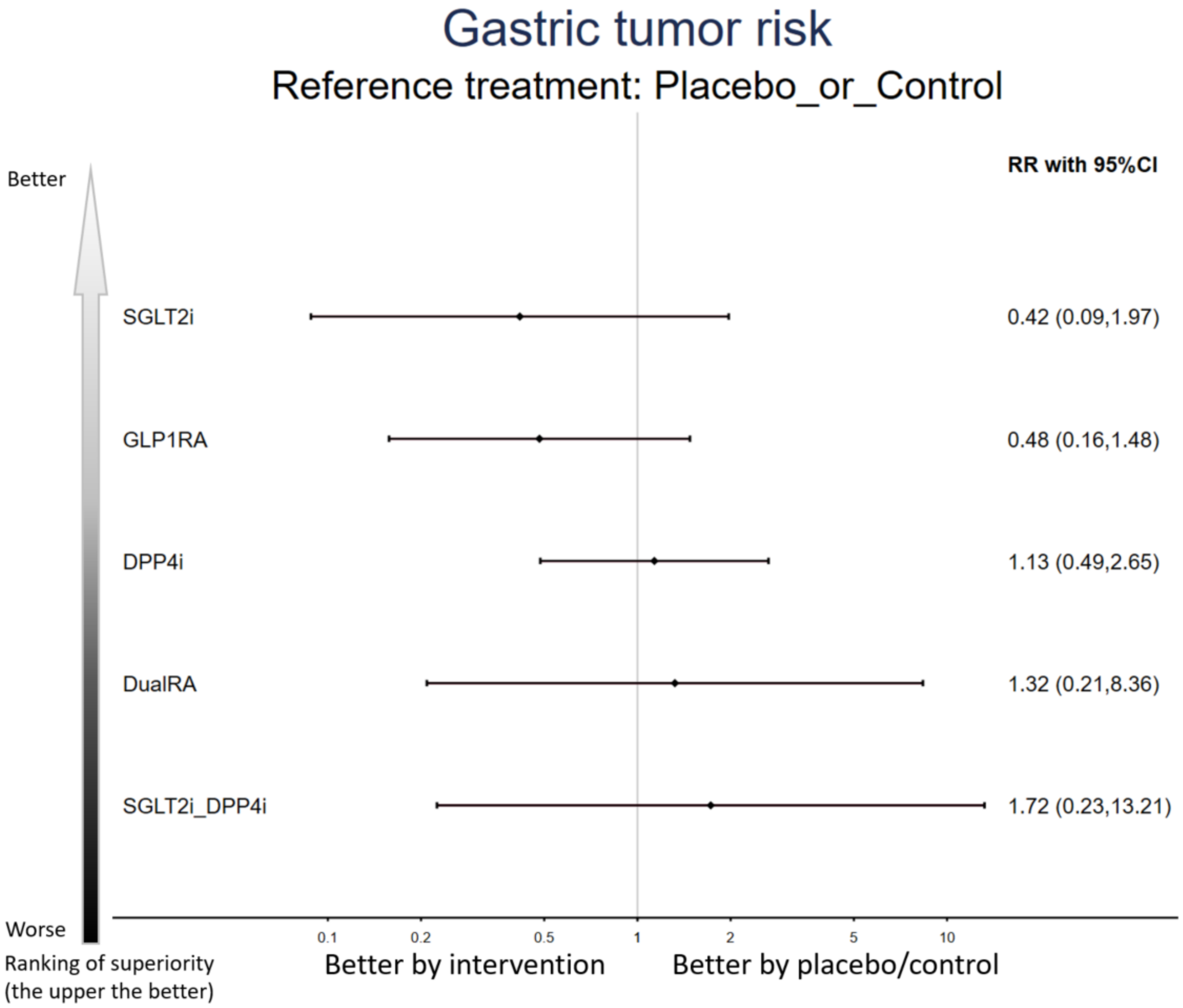

Figure S2E Forest plot of NMA of secondary outcome: *Helicobacter pylori* risk

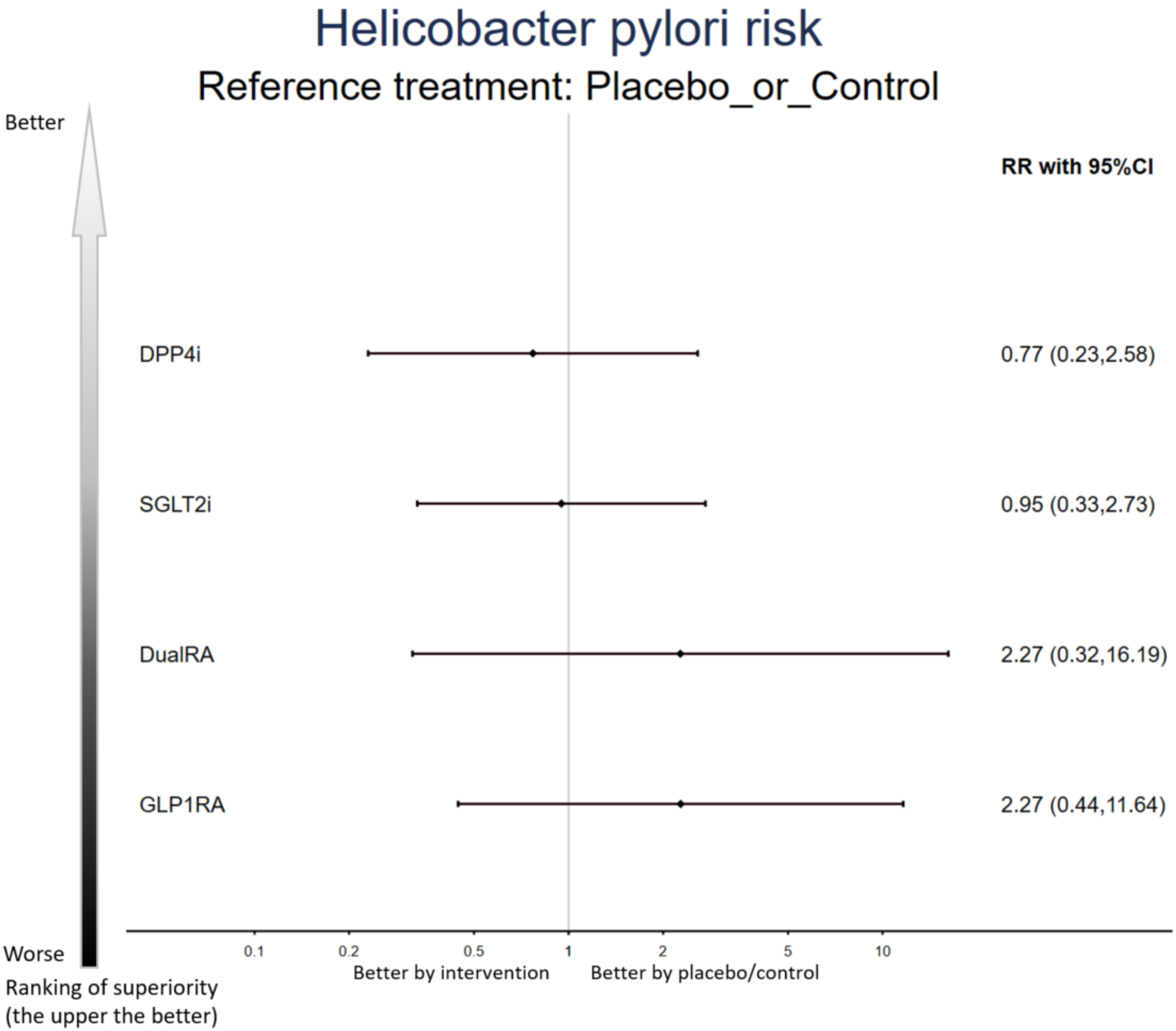

Figure S2F Forest plot of NMA of acceptability: drop-out rate

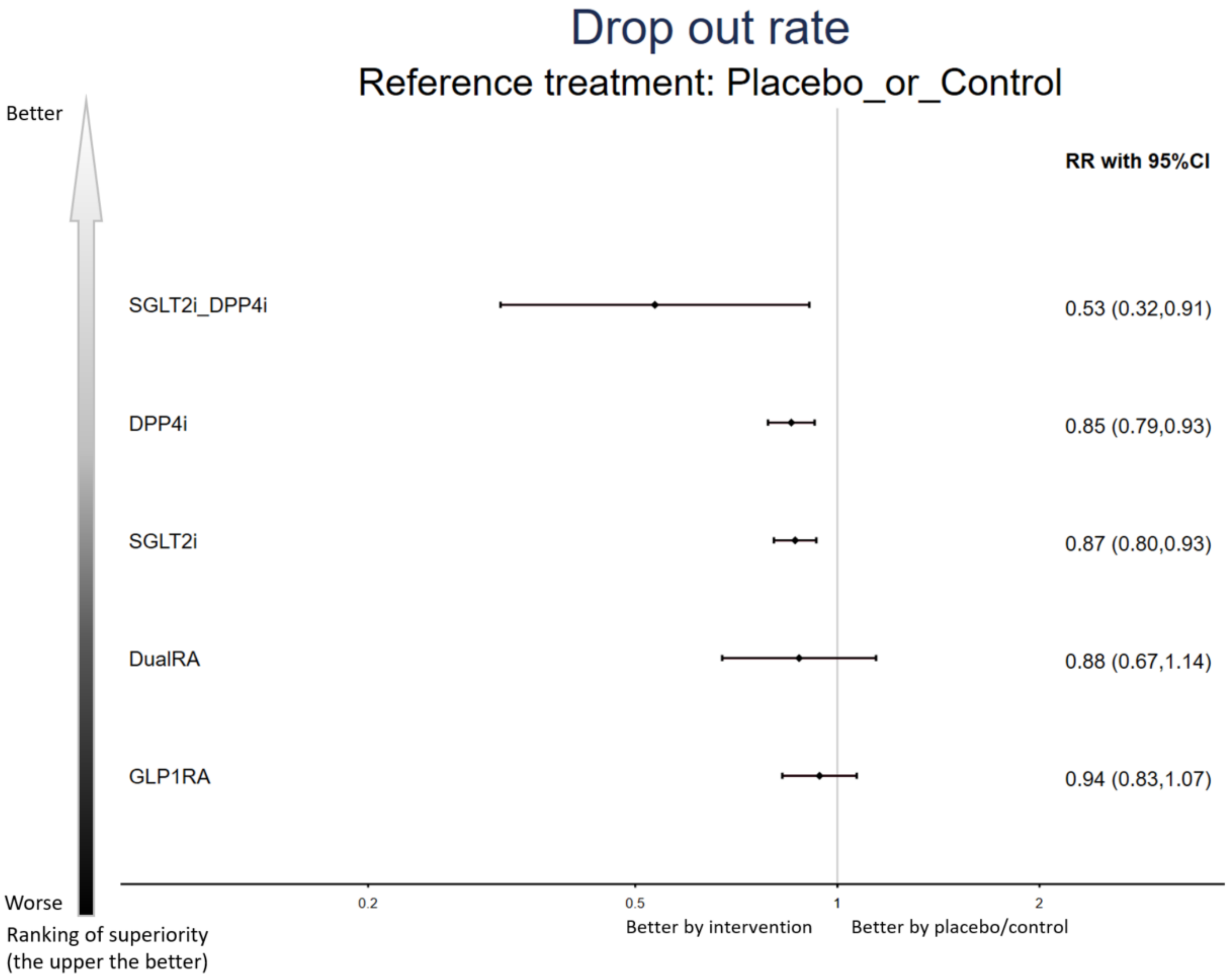

Figure S2G Forest plot of NMA of primary outcome: gastric tumor risk – dose level

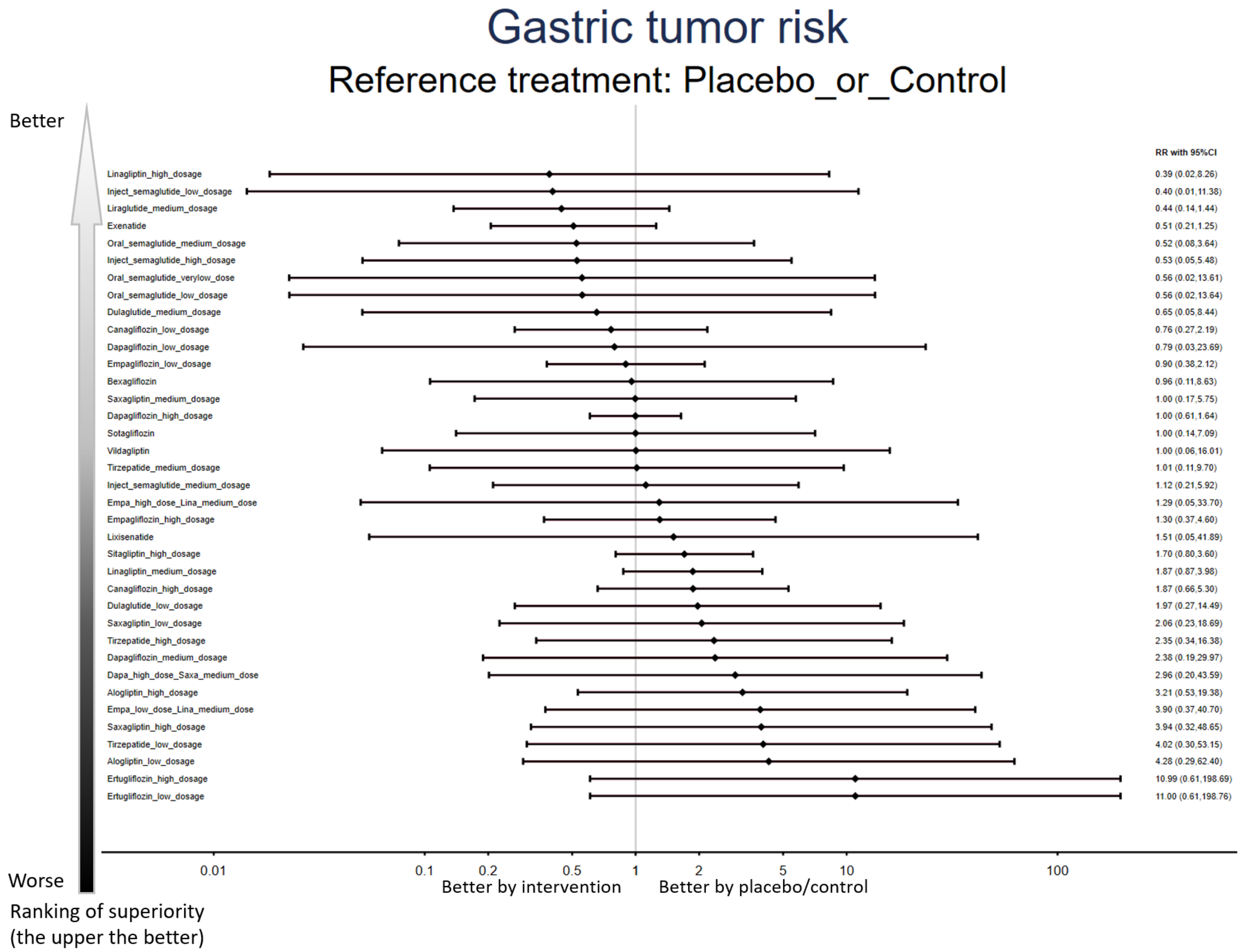

Abbreviation for Figure S2A-S2G:

95%CIs: 95% confidence intervals; DPP4 inhibitor: dipeptidyl peptidase 4 inhibitor; GLP-1 agonist: glucagon-like peptide-1 agonist; NMA: network meta-analysis; RCT: randomized controlled trial; RR: risk ratio; SGLT2 inhibitor: sodium–glucose cotransporter 2 inhibitor

Figure S3 Individual study result of primary outcome: gastric tumor risk

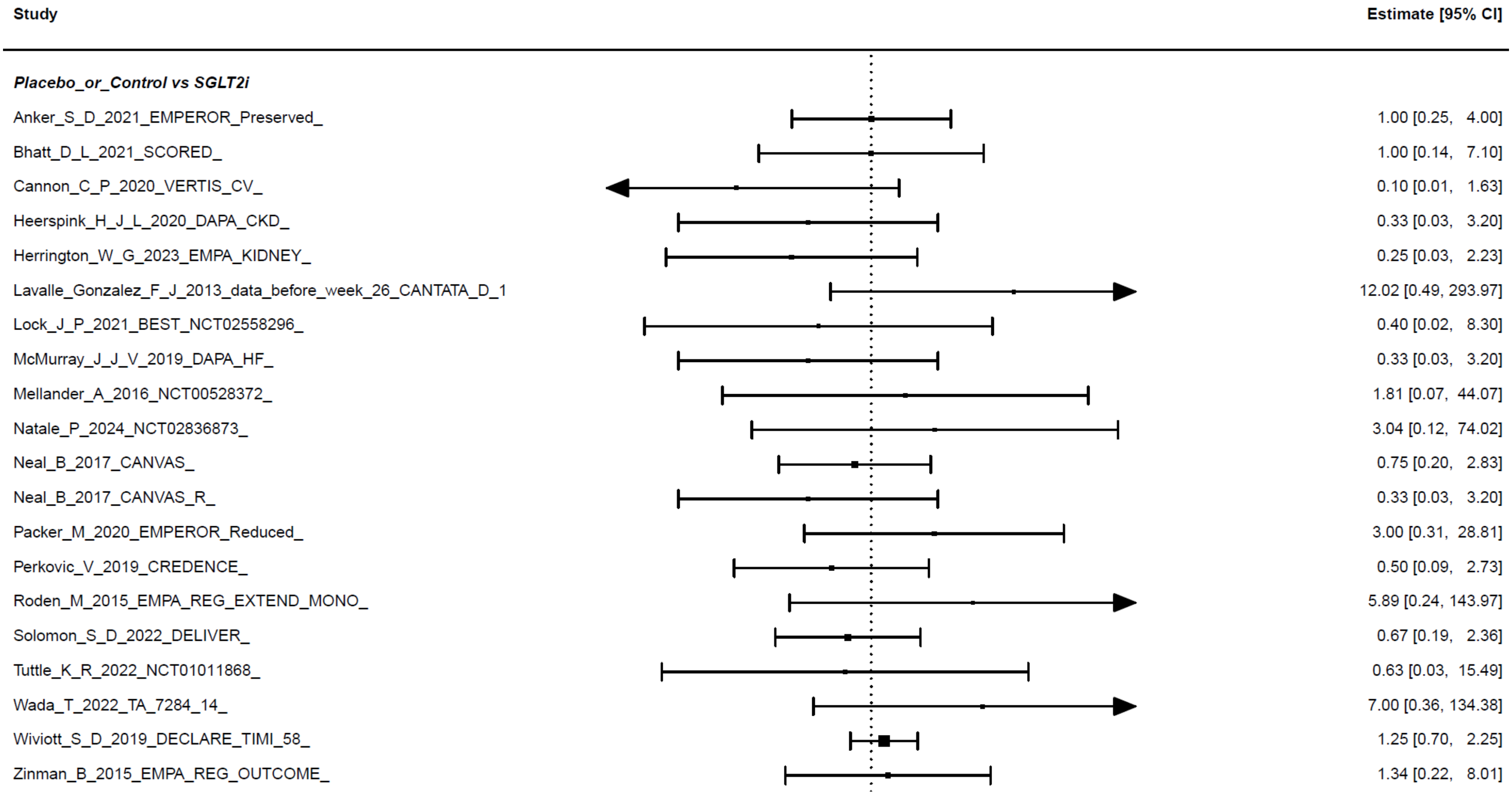

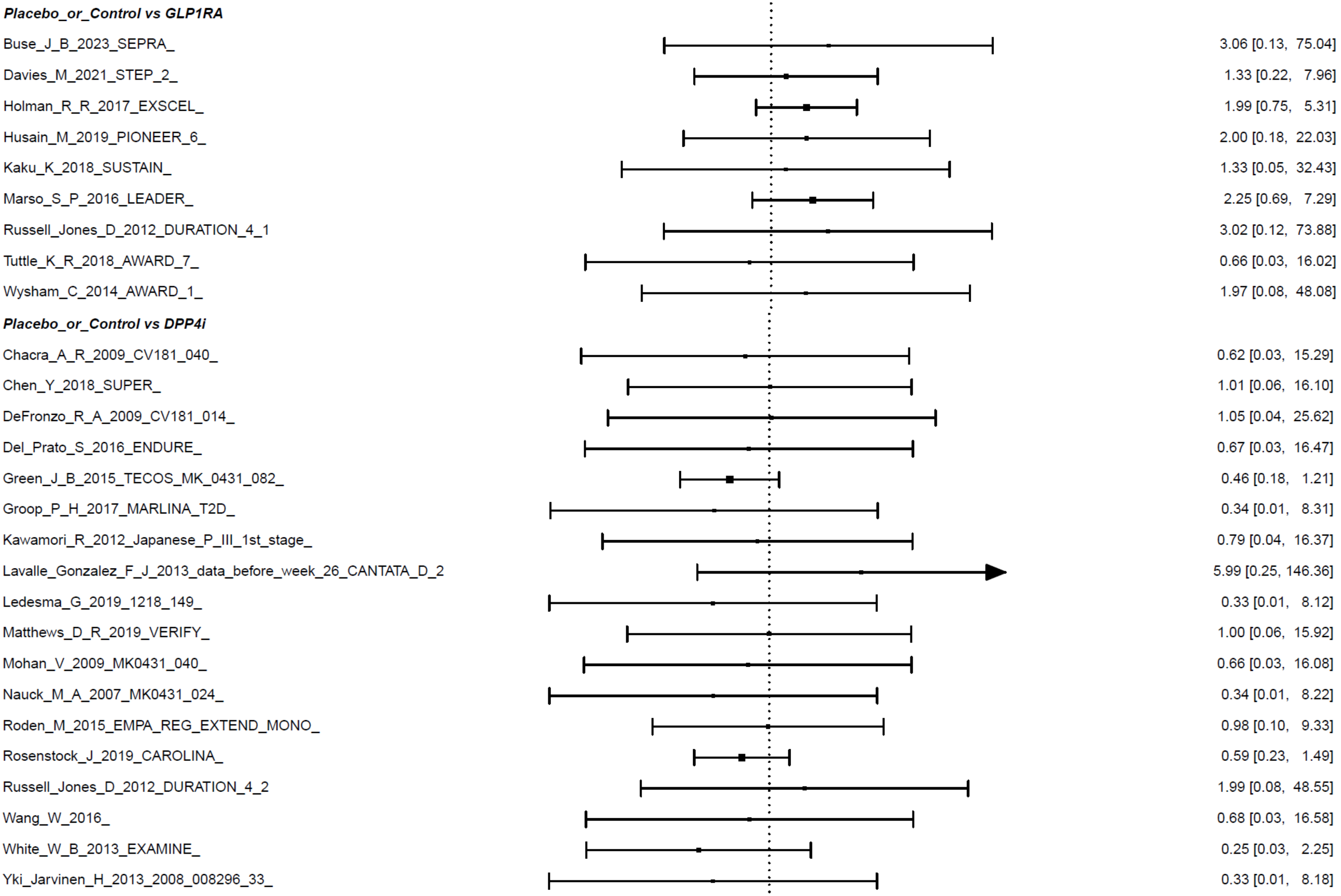
Placebo_or_Control vs GLP1RA
Buse_J_B_2023_SEPRA_ 3.06 [0.13, 75.04]
Davies_M_2021_STEP_2_ 1.33 [0.22, 7.96]
Holman_R_R_2017_EXSCEL_ 1.99 [0.75, 5.31]
Husain_M_2019_PIONEER_6_ 2.00 [0.18, 22.03]
Kaku_K_2018_SUSTAIN_ 1.33 [0.05, 32.43]
Marso_S_P_2016_LEADER_ 2.25 [0.69, 7.29]
Russell_Jones_D_2012_DURATION_4_1 3.02 [0.12, 73.88]
Tuttle_K_R_2018_AWARD_7_ 0.66 [0.03, 16.02]
Wysham_C_2014_AWARD_1_ 1.97 [0.08, 48.08]
Placebo_or_Control vs DPP4i
Chacra_A_R_2009_CV181_040_ 0.62 [0.03, 15.29]
Chen_Y_2018_SUPER_ 1.01 [0.06, 16.10]
DeFronzo_R_A_2009_CV181_014_ 1.05 [0.04, 25.62]
Del_Prato_S_2016_ENDURE_ 0.67 [0.03, 16.47]
Green_J_B_2015_TECOS_MK_0431_082_ 0.46 [0.18, 1.21]
Groop_P_H_2017_MARLINA_T2D_ 0.34 [0.01, 8.31]
Kawamori_R_2012_Japanese_P_III_1st_stage_ 0.79 [0.04, 16.37]
Lavalle_Gonzalez_F_J_2013_data_before_week_26_CANTATA_D_2 5.99 [0.25, 146.36]
Ledesma_G_2019_1218_149_ 0.33 [0.01, 8.12]
Matthews_D_R_2019_VERIFY_ 1.00 [0.06, 15.92]
Mohan_V_2009_MK0431_040_ 0.66 [0.03, 16.08]
Nauck_M_A_2007_MK0431_024_ 0.34 [0.01, 8.22]
Roden_M_2015_EMPA_REG_EXTEND_MONO_ 0.98 [0.10, 9.33]
Rosenstock_J_2019_CAROLINA_ 0.59 [0.23, 1.49]
Russell_Jones_D_2012_DURATION_4_2 1.99 [0.08, 48.55]
Wang_W_2016_ 0.68 [0.03, 16.58]
White_W_B_2013_EXAMINE_ 0.25 [0.03, 2.25]
Yki_Jarvinen_H_2013_2008_008296_33_ 0.33 [0.01, 8.18]

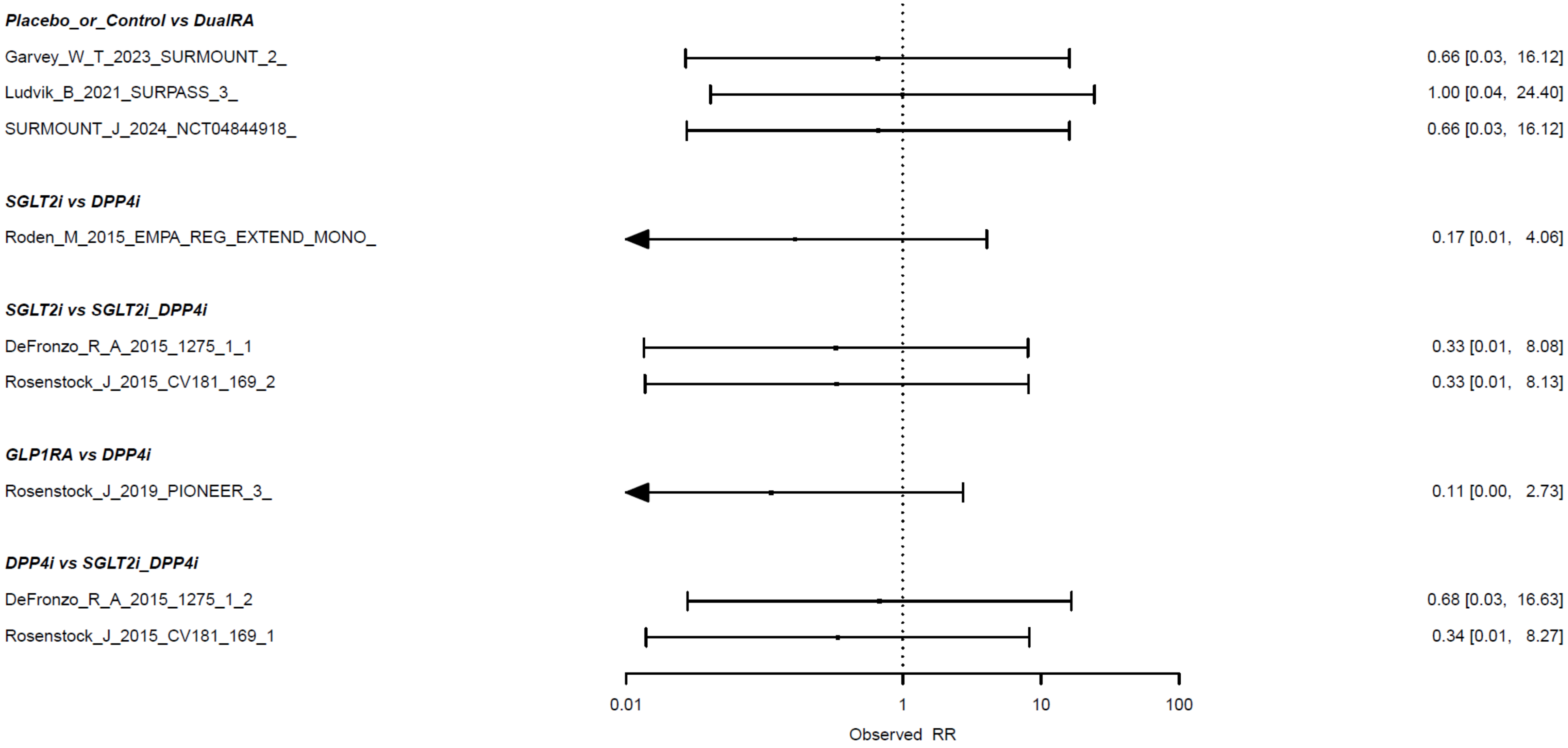
Placebo_or_Control vs DualRA
Garvey_W_T_2023_SURMOUNT_2_ 0.66 [0.03, 16.12]
Ludvik_B_2021_SURPASS_3_ 1.00 [0.04, 24.40]
SURMOUNT_J_2024_NCT04844918_ 0.66 [0.03, 16.12]
SGLT2i vs DPP4i
Roden_M_2015_EMPA_REG_EXTEND_MONO_ 0.17 [0.01, 4.06]
SGLT2i vs SGLT2i_DPP4i
DeFronzo_R_A_2015_1275_1_1 0.33 [0.01, 8.08]
Rosenstock_J_2015_CV181_169_2 0.33 [0.01, 8.13]
GLP1RA vs DPP4i
Rosenstock_J_2019_PIONEER_3_ 0.11 [0.00, 2.73]
DPP4i vs SGLT2i_DPP4i
DeFronzo_R_A_2015_1275_1_2 0.68 [0.03, 16.63]
Rosenstock_J_2015_CV181_169_1 0.34 [0.01, 8.27]
0.01
1
10
100
Observed RR

Abbreviation for Figure S3:

95%CIs: 95% confidence intervals; DPP4 inhibitor: dipeptidyl peptidase 4 inhibitor; GLP-1 agonist: glucagon-like peptide-1 agonist; NMA: network meta-analysis; RCT: randomized controlled trial; RR: risk ratio; SGLT2 inhibitor: sodium–glucose cotransporter 2 inhibitor

Figure S4 Funnel plot for primary outcome: gastric tumor risk

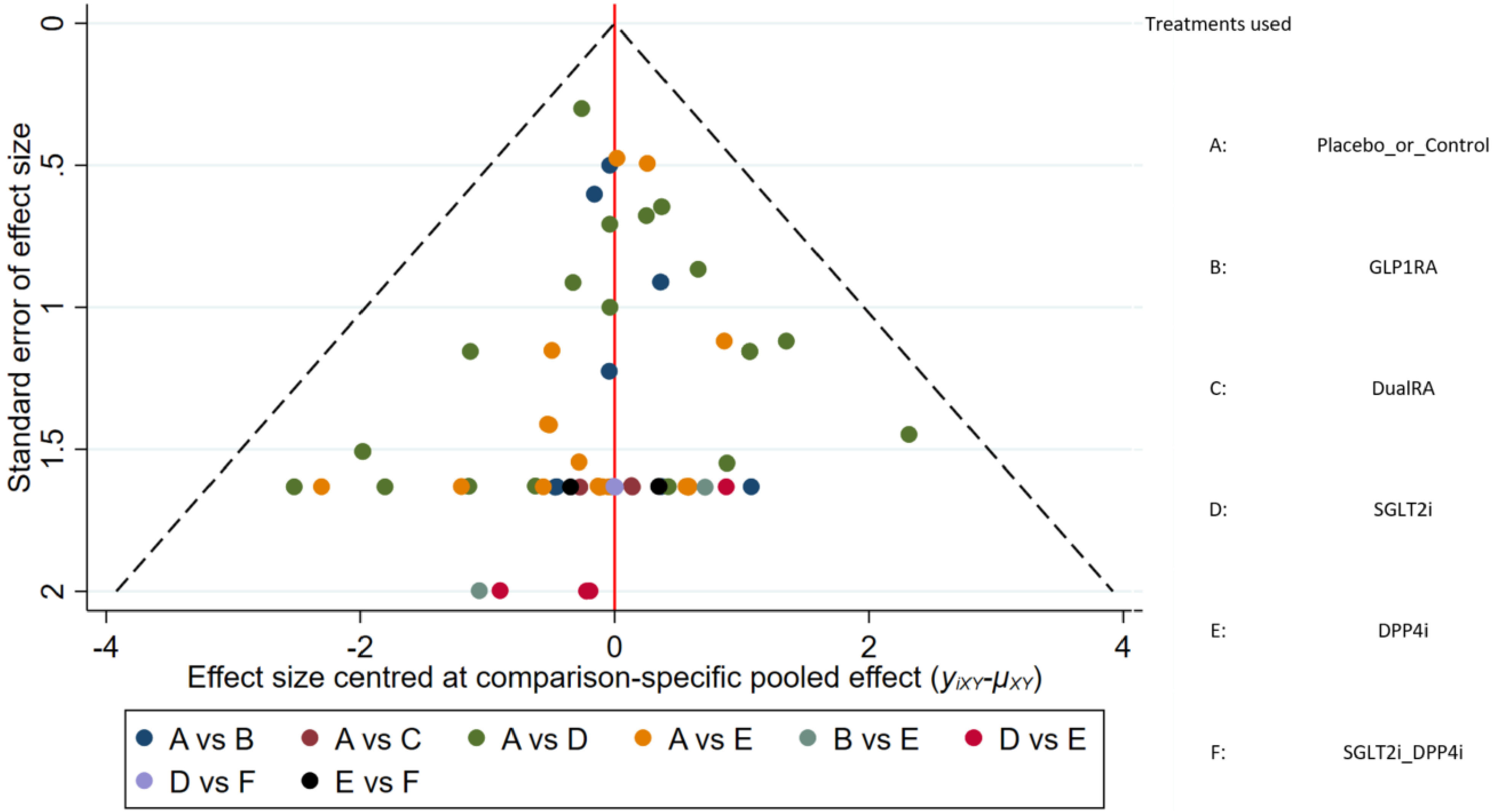

Abbreviation for Figure S4:

95%CIs: 95% confidence intervals; DPP4 inhibitor: dipeptidyl peptidase 4 inhibitor; GLP-1 agonist: glucagon-like peptide-1 agonist; NMA: network meta-analysis; RCT: randomized controlled trial; RR: risk ratio; SGLT2 inhibitor: sodium–glucose cotransporter 2 inhibitor

Figure S5 Egger test for primary outcome: gastric tumor risk

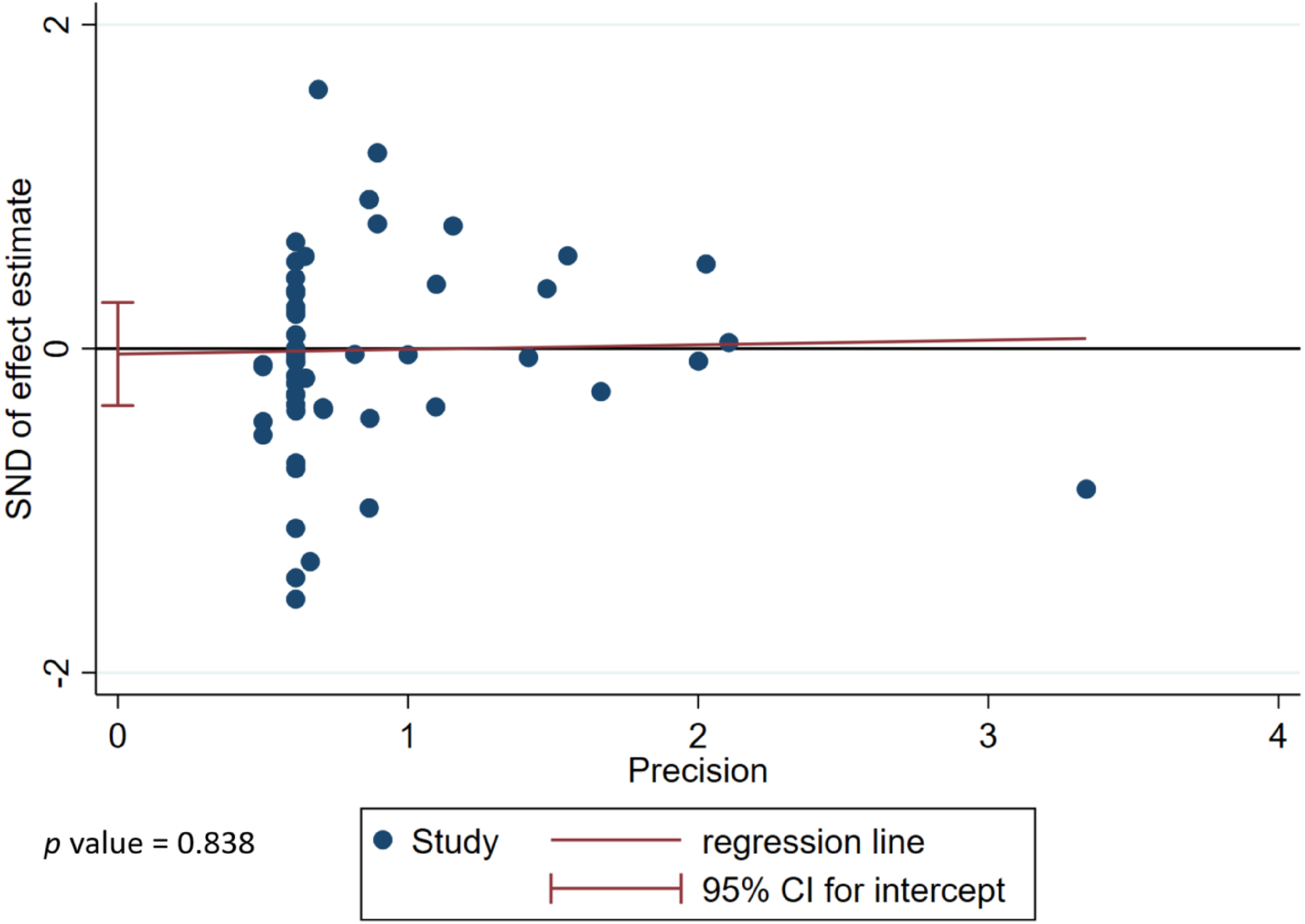

p value = 0.838

Abbreviation for Figure S5:

95%CIs: 95% confidence intervals; DPP4 inhibitor: dipeptidyl peptidase 4 inhibitor; GLP-1 agonist: glucagon-like peptide-1 agonist; NMA: network meta-analysis; RCT: randomized controlled trial; RR: risk ratio; SGLT2 inhibitor: sodium–glucose cotransporter 2 inhibitor

Figure S6 Bayesian network meta-analysis of primary outcome: gastric tumor risk

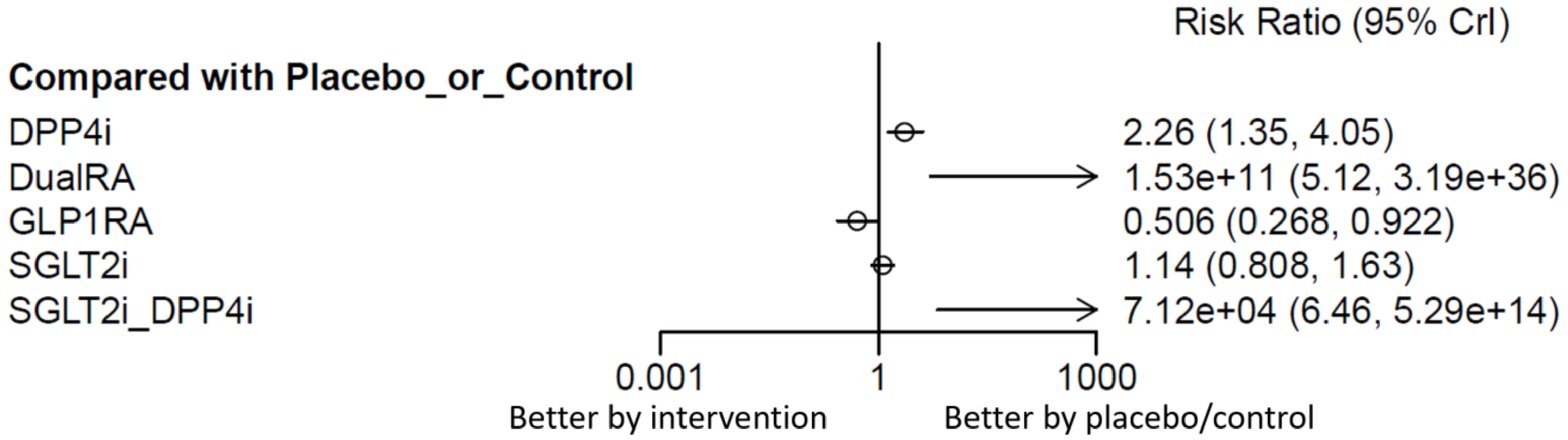

Figure S7 Risk of bias tool 2.0

Study name	Randomization process	Intervention adherence	Missing outcome data	Outcome measurement	Selective reporting	Overall RoB
Anker, S.D. (2021) (EMPEROR-Preserved)	Green	Green	Green	Green	Green	Green
Bhatt, D.L. (2021) (SCORED)	Green	Green	Green	Green	Red	Red
Buse, J.B. (2023) (SEPRA)	Green	Yellow	Green	Green	Green	Yellow
Cannon, C.P. (2020) (VERTIS CV)	Green	Green	Green	Green	Green	Green
Chacra, A.R. (2009) (CV181-040)	Green	Green	Green	Green	Green	Green
Chen, Y. (2018) (SUPER)	Green	Green	Green	Green	Green	Green
Davies, M. (2021) (STEP 2)	Green	Green	Green	Green	Green	Green
DeFronzo, R.A. (2009) (CV181-014)	Green	Green	Green	Green	Green	Green
DeFronzo, R.A. (2015) (1275.1)	Green	Green	Yellow	Green	Green	Yellow
Del Prato, S. (2016) (ENDURE)	Yellow	Green	Yellow	Green	Red	Red
Garvey, W.T. (2023) (SURMOUNT-2)	Green	Green	Green	Green	Green	Green
Green, J.B. (2015) (TECOS - MK-0431-082)	Green	Green	Green	Green	Green	Green
Groop, P.H. (2017) (MARLINA-T2D)	Green	Green	Green	Green	Green	Green
Heerspink, H.J.L. (2020) (DAPA-CKD)	Green	Green	Green	Green	Green	Yellow
Herrington, W.G. (2023) (EMPA-KIDNEY)	Green	Green	Green	Green	Green	Green
Holman, R.R. (2017) (EXSCEL)	Green	Green	Green	Green	Green	Green
Husain, M. (2019) (PIONEER 6)	Green	Green	Green	Green	Green	Green
Kaku, K. (2018) (SUSTAIN)	Green	Yellow	Green	Green	Green	Yellow
Kawamori, R. (2012) (Japanese P III) (1st stage)	Green	Green	Green	Green	Green	Green
Lavalle-Gonzalez, F.J. (2013) (data before week 26) (CANTATA-D)	Green	Green	Green	Green	Green	Green
Ledesma, G. (2019) (1218.149)	Green	Green	Yellow	Green	Green	Yellow
Lock, J.P. (2021) (BEST) (NCT02558296)	Green	Yellow	Red	Yellow	Yellow	Red
Ludvik, B. (2021) (SURPASS-3)	Green	Yellow	Green	Green	Green	Yellow
Marso, S.P. (2016) (LEADER)	Green	Green	Green	Green	Green	Green
Matthews, D.R. (2019) (VERIFY)	Green	Green	Yellow	Green	Green	Yellow
McMurray, J.J.V. (2019) (DAPA-HF)	Green	Green	Green	Green	Green	Yellow

Mellander, A. (2016) (NCT00528372)	🟢	🟢	🟢	🟢	🟢	🟢
Mohan, V. (2009) (MK0431-040)	🟢	🟢	🟢	🟢	🟢	🟢
Natale, P. (2024) (NCT02836873)	🟢	🟢	🟢	🟢	🟢	🟢
Nauck, M.A. (2007) (MK0431-024)	🟢	🟢	🟡	🟢	🟢	🟡
Neal, B. (2017) (CANVAS)	🟢	🟢	🟢	🟢	🟢	🟢
Neal, B. (2017) (CANVAS-R)	🟢	🟢	🟢	🟢	🟢	🟢
Packer, M. (2020) (EMPEROR-Reduced)	🟢	🟢	🟢	🟢	🟢	🟢
Perkovic, V. (2019) (CREDENCE)	🟢	🟢	🟢	🟢	🟢	🟢
Roden, M. (2015) (EMPA-REG EXTEND MONO)	🟢	🟢	🟡	🟢	🟢	🟡
Rosenstock, J. (2013) (GetGoal-X)	🟢	🟡	🟢	🟢	🟢	🟡
Rosenstock, J. (2015) (CV181-169)	🟢	🟢	🟢	🟢	🟢	🟢
Rosenstock, J. (2019) (CAROLINA)	🟢	🟢	🟢	🟢	🟢	🟢
Rosenstock, J. (2019) (PIONEER 3)	🟢	🟢	🟢	🟢	🟢	🟢
Russell-Jones, D. (2012) (DURATION-4)	🟢	🟢	🟢	🟢	🟢	🟢
Seino, Y. (2012) (NCT01318135)	🟢	🟡	🟢	🟢	🟢	🟡
Solomon, S.D. (2022) (DELIVER)	🟢	🟢	🟢	🟢	🟢	🟡
SURMOUNT-J (2024) (NCT04844918)	🟢	🟡	🔴	🟡	🟡	🔴
Tuttle, K. R. (2018) (AWARD-7)	🟢	🟢	🟢	🟢	🟢	🟢
Tuttle, K.R. (2022) (NCT01011868)	🟢	🟢	🟢	🟢	🟢	🟢
Wada, T. (2022) (TA-7284-14)	🟢	🟢	🟢	🟢	🟢	🟢
Wang, W. (2016)	🟢	🟢	🟢	🟢	🟢	🟢
White, W.B. (2013) (EXAMINE)	🟢	🟢	🟡	🟢	🟢	🟡
Wiviott, S.D. (2019) (DECLARE-TIMI 58)	🟢	🟢	🟢	🟢	🟢	🟡
Wysham, C. (2014) (AWARD-1)	🟢	🟢	🟢	🟢	🟢	🟢
Yki-Järvinen, H. (2013) (2008-008296-33)	🟢	🟢	🟡	🟢	🟢	🟡
Zinman, B. (2015) (EMPA-REG OUTCOME)	🟢	🟢	🟢	🟢	🟢	🟢

Table S1: PRISMA 2020 checklist of the current network meta-analysis

Section and Topic	Item #	Checklist item	Page where item is reported
**TITLE**			
Title	1	Identify the report as a systematic review.	1
**ABSTRACT**			
Abstract	2	See the PRISMA 2020 for Abstracts checklist.	7-8
**INTRODUCTION**			
Rationale	3	Describe the rationale for the review in the context of existing knowledge.	9-10
Objectives	4	Provide an explicit statement of the objective(s) or question(s) the review addresses.	9-10
**METHODS**			
Eligibility criteria	5	Specify the inclusion and exclusion criteria for the review and how studies were grouped for the syntheses.	11-12
Information sources	6	Specify all databases, registers, websites, organisations, reference lists and other sources searched or consulted to identify studies. Specify the date when each source was last searched or consulted.	11-12
Search strategy	7	Present the full search strategies for all databases, registers and websites, including any filters and limits used.	11-12
Selection process	8	Specify the methods used to decide whether a study met the inclusion criteria of the review, including how many reviewers screened each record and each report retrieved, whether they worked independently, and if applicable, details of automation tools used in the process.	11-12
Data collection process	9	Specify the methods used to collect data from reports, including how many reviewers collected data from each report, whether they worked independently, any processes for obtaining or confirming data from study investigators, and if applicable, details of automation tools used in the process.	11-12
Data items	10a	List and define all outcomes for which data were sought. Specify whether all results that were compatible with each outcome domain in each study were sought (e.g. for all measures, time points, analyses), and if not, the methods used to decide which results to collect.	12-13
	10b	List and define all other variables for which data were sought (e.g. participant and intervention characteristics, funding sources). Describe any assumptions made about any missing or unclear information.	12-13
Study risk of bias assessment	11	Specify the methods used to assess risk of bias in the included studies, including details of the tool(s) used, how many reviewers assessed each study and whether they worked independently, and if applicable, details of automation tools used in the process.	12-13
Effect measures	12	Specify for each outcome the effect measure(s) (e.g. risk ratio, mean difference) used in the synthesis or presentation of results.	12-13
Synthesis methods	13a	Describe the processes used to decide which studies were eligible for each synthesis (e.g. tabulating the study intervention characteristics and comparing against the planned groups for each synthesis (item #5)).	12-13
	13b	Describe any methods required to prepare the data for presentation or synthesis, such as handling of missing summary statistics, or data conversions.	13-15
	13c	Describe any methods used to tabulate or visually display results of individual studies and syntheses.	13-15
	13d	Describe any methods used to synthesize results and provide a rationale for the choice(s). If meta-analysis was performed, describe the model(s), method(s) to identify the presence and extent of statistical heterogeneity, and software package(s) used.	13-15
	13e	Describe any methods used to explore possible causes of heterogeneity among study results (e.g. subgroup analysis, meta-regression).	13-15
	13f	Describe any sensitivity analyses conducted to assess robustness of the synthesized results.	13-15

Section and Topic	Item #	Checklist item	Page where item is reported
Reporting bias assessment	14	Describe any methods used to assess risk of bias due to missing results in a synthesis (arising from reporting biases).	13-15
Certainty assessment	15	Describe any methods used to assess certainty (or confidence) in the body of evidence for an outcome.	13-15
**RESULTS**			
Study selection	16a	Describe the results of the search and selection process, from the number of records identified in the search to the number of studies included in the review, ideally using a flow diagram.	16-17, Fig 1, eTab 2
	16b	Cite studies that might appear to meet the inclusion criteria, but which were excluded, and explain why they were excluded.	16-17, eTab 4
Study characteristics	17	Cite each included study and present its characteristics.	16-17, eTab 5
Risk of bias in studies	18	Present assessments of risk of bias for each included study.	16-17, eFig 7
Results of individual studies	19	For all outcomes, present, for each study: (a) summary statistics for each group (where appropriate) and (b) an effect estimate and its precision (e.g. confidence/credible interval), ideally using structured tables or plots.	16-17, eFig 3
Results of syntheses	20a	For each synthesis, briefly summarise the characteristics and risk of bias among contributing studies.	17-18, Fig 2
	20b	Present results of all statistical syntheses conducted. If meta-analysis was done, present for each the summary estimate and its precision (e.g. confidence/credible interval) and measures of statistical heterogeneity. If comparing groups, describe the direction of the effect.	17-18, Fig 3
	20c	Present results of all investigations of possible causes of heterogeneity among study results.	17-18, eTab 8-9
	20d	Present results of all sensitivity analyses conducted to assess the robustness of the synthesized results.	17-18
Reporting biases	21	Present assessments of risk of bias due to missing results (arising from reporting biases) for each synthesis assessed.	17-18, eFig 7
Certainty of evidence	22	Present assessments of certainty (or confidence) in the body of evidence for each outcome assessed.	17-18
**DISCUSSION**			
Discussion	23a	Provide a general interpretation of the results in the context of other evidence.	19-21
	23b	Discuss any limitations of the evidence included in the review.	21-22
	23c	Discuss any limitations of the review processes used.	21-22
	23d	Discuss implications of the results for practice, policy, and future research.	23
**OTHER INFORMATION**			
Registration and protocol	24a	Provide registration information for the review, including register name and registration number, or state that the review was not registered.	8
	24b	Indicate where the review protocol can be accessed, or state that a protocol was not prepared.	8
	24c	Describe and explain any amendments to information provided at registration or in the protocol.	8
Support	25	Describe sources of financial or non-financial support for the review, and the role of the funders or sponsors in the review.	24
Competing interests	26	Declare any competing interests of review authors.	24
Availability of data,	27	Report which of the following are publicly available and where they can be found: template data collection forms; data extracted from included	24

Section and Topic	Item #	Checklist item	Page where item is reported
code and other materials		studies; data used for all analyses; analytic code; any other materials used in the review.	

The current checklist followed the latest PRISMA 2020 guideline [1].

Table S2: Keyword used in each database and search results

Database	Keyword	Filter	Date	Result
PubMed	(gastric tumor) AND (glucagon-like peptide-1 receptor agonist OR Sodium Glucose Cotransporter 2 Inhibitor OR dipeptidyl peptidase-4 inhibitor OR lixisenatide OR orforglipron OR exenatide OR semaglutide OR liraglutide OR albiglutide OR dulaglutide OR tirzepatide OR bexagliflozin OR canagliflozin OR dapagliflozin OR empagliflozin OR ertugliflozin OR ipragliflozin OR luseogliflozin OR remogliflozin OR sergliflozin OR sotagliflozin OR tofogliflozin OR henagliflozin OR janagliflozin OR mizagliflozin OR velagliflozin OR enavogliflozin OR licogliflozin OR rongliflozin OR sitagliptin OR vildagliptin OR saxagliptin OR linagliptin OR gemigliptin OR anagliptin OR teneligliptin OR alogliptin OR trelagliptin OR omarigliptin OR evogliptin OR gosogliptin OR dutogliptin OR neogliptin OR retagliptin OR denagliptin OR cofrogliptin OR fotagliptin OR prusogliptin OR cetagliptin OR berberine OR retatrutide OR imeglimin OR dorzagliatin OR petrelintide OR teplizumab) AND (random OR randomized OR randomised)	N/A	2026/01/10	8
ClinicalKey	(gastric tumor) AND (glucagon-like peptide-1 receptor agonist OR Sodium Glucose Cotransporter 2 Inhibitor OR dipeptidyl peptidase-4 inhibitor OR lixisenatide OR orforglipron OR exenatide OR semaglutide OR liraglutide OR albiglutide OR dulaglutide OR tirzepatide OR bexagliflozin OR canagliflozin OR dapagliflozin OR empagliflozin OR ertugliflozin OR ipragliflozin OR luseogliflozin OR remogliflozin OR sergliflozin OR sotagliflozin OR tofogliflozin OR henagliflozin OR janagliflozin OR mizagliflozin OR velagliflozin OR enavogliflozin OR licogliflozin OR rongliflozin OR sitagliptin OR vildagliptin	N/A	2026/01/10	741

	OR saxagliptin OR linagliptin OR gemigliptin OR anagliptin OR teneligliptin OR alogliptin OR trelagliptin OR omarigliptin OR evogliptin OR gosogliptin OR dutogliptin OR neogliptin OR retagliptin OR denagliptin OR cofrogliptin OR fotagliptin OR prusogliptin OR cetagliptin OR berberine OR retatrutide OR imeglimin OR dorzagliatin OR petrelintide OR teplizumab) AND (random OR randomized OR randomised)			
Cochrane CENTRAL	(gastric tumor) AND (glucagon-like peptide-1 receptor agonist OR Sodium Glucose Cotransporter 2 Inhibitor OR dipeptidyl peptidase-4 inhibitor OR lixisenatide OR orforglipron OR exenatide OR semaglutide OR liraglutide OR albiglutide OR dulaglutide OR tirzepatide OR bexagliflozin OR canagliflozin OR dapagliflozin OR empagliflozin OR ertugliflozin OR ipragliflozin OR luseogliflozin OR remogliflozin OR sergliflozin OR sotagliflozin OR tofogliflozin OR henagliflozin OR janagliflozin OR mizagliflozin OR velagliflozin OR enavogliflozin OR licogliflozin OR rongliflozin OR sitagliptin OR vildagliptin OR saxagliptin OR linagliptin OR gemigliptin OR anagliptin OR teneligliptin OR alogliptin OR trelagliptin OR omarigliptin OR evogliptin OR gosogliptin OR dutogliptin OR neogliptin OR retagliptin OR denagliptin OR cofrogliptin OR fotagliptin OR prusogliptin OR cetagliptin OR berberine OR retatrutide OR imeglimin OR dorzagliatin OR petrelintide OR teplizumab) AND (random OR randomized OR randomised)	N/A	2026/01/10	7
Embase	(gastric tumor) AND (glucagon-like peptide-1 receptor agonist OR Sodium Glucose Cotransporter 2 Inhibitor OR dipeptidyl peptidase-4 inhibitor) AND (random OR randomized OR randomised)	N/A	2026/01/10	188
ProQuest	(gastric tumor) AND (glucagon-like peptide-1 receptor agonist OR Sodium Glucose Cotransporter 2 Inhibitor OR dipeptidyl peptidase-4 inhibitor) AND (random OR	N/A	2026/01/10	3399

	randomized OR randomised)			
ScienceDirect	(gastric tumor) AND (glucagon-like peptide-1 receptor agonist OR Sodium Glucose Cotransporter 2 Inhibitor OR dipeptidyl peptidase-4 inhibitor) AND (random OR randomized OR randomised)	N/A	2026/01/10	3259
Web of Science	(gastric tumor) AND (glucagon-like peptide-1 receptor agonist OR Sodium Glucose Cotransporter 2 Inhibitor OR dipeptidyl peptidase-4 inhibitor) AND (random OR randomized OR randomised)	N/A	2026/01/10	2
ClinicalTrials.gov	(gastric tumor) AND (glucagon-like peptide-1 receptor agonist OR Sodium Glucose Cotransporter 2 Inhibitor OR dipeptidyl peptidase-4 inhibitor OR lixisenatide OR orforglipron OR exenatide OR semaglutide OR liraglutide OR albiglutide OR dulaglutide OR tirzepatide OR bexagliflozin OR canagliflozin OR dapagliflozin OR empagliflozin OR ertugliflozin OR ipragliflozin OR luseogliflozin OR remogliflozin OR sergliflozin OR sotagliflozin OR tofogliflozin OR henagliflozin OR janagliflozin OR mizagliflozin OR velagliflozin OR enavogliflozin OR licogliflozin OR rongliflozin OR sitagliptin OR vildagliptin OR saxagliptin OR linagliptin OR gemigliptin OR anagliptin OR teneligliptin OR alogliptin OR trelagliptin OR omarigliptin OR evogliptin OR gosogliptin OR dutogliptin OR neogliptin OR retagliptin OR denagliptin OR cofrogliptin OR fotagliptin OR prusogliptin OR cetagliptin OR berberine OR retatrutide OR imeglimin OR dorzagliatin OR petrelintide OR teplizumab) AND (random OR randomized OR randomised)	N/A	2026/01/10	1

Abbreviation: N/A: not applied

Table S3: Dosage stratification (stratified according to the included RCTs)

Medication	Low-dose	Medium-dose	High-dose
Alogliptin	12.5 mg/day	NA	25 mg/day
Canagliflozin	50-100 mg/day	200 mg/day	300-600 mg/day
Dapagliflozin	2.5 mg/day	5 mg/day	10 mg/day
Dulaglutide	<1.5 mg/week	1.5 mg/week	>1.5 mg/week
Empagliflozin	1-10 mg/day	NA	25-50 mg/day
Ertugliflozin	5 mg/day	NA	15 mg/day
Injectable semaglutide	0.05-0.5 mg/week	1.0-1.7 mg/week	2.0-2.4 mg/week
Linagliptin	2.5 mg/day	5 mg/day	10 mg/day
Liraglutide	1.2 mg/day	1.8 mg/day	3.0 mg/day
Oral semaglutide	7-10 mg/day	14 mg/day	20-25 mg/day
Saxagliptin	2.5 mg/day	5 mg/day	10 mg/day
Sitagliptin	25 mg/day	50 mg/day	100 mg/day
Tirzepatide	1-5 mg/week	10 mg/week	15 mg/week

Abbreviation: NA: not applied; RCT: randomized controlled trial

Table S4: Excluded studies and reason

Reason	Numbers	References
Animal study	1	[2]
Meta-analysis	1	[3]
Network meta-analysis	2	[4,5]
Not randomized controlled trial	1	[6]
Not report target outcome	289	[7-150] [151-295]
Studies randomizing subjects with high baseline imbalance or improper randomization	23	[296-318]

Table S5: Characteristics of the included studies

Study name	Baseline illness	Comparison	Subjects	Mean age (year)	Female (%)	Treatment duration	Route	Category	ClinicalTrials.gov	Country
Natale, P. (2024) (NCT02836873)[319]	patients with type 2 diabetes mellitus and chronic kidney disease	Bexagliflozin 20mg/day<br>Placebo	157<br>155	69.3±8.4<br>69.9±8.3	41.4<br>32.9	24 weeks	oral	SGLT2 inhibitor	NCT02836873	Multiple countries
SURMOUNT-J (2024) (NCT04844918)[320]	patients with obesity	Tirzepatide 10mg/week<br>Tirzepatide 15mg/week<br>Placebo	73<br>77<br>75	49.0±10.9<br>51.1±10.3<br>52.3±10.9	41.1<br>41.6<br>40.0	72 weeks	injection	GLP-1 agonist	NCT04844918	Japan
Buse, J.B. (2023) (SEPRA)[321]	patients with type 2 diabetes mellitus	Inject semaglutide<br>Control	644<br>634	57.5±11.3<br>57.2±11.0	48.1<br>43.5	104 weeks	injection	GLP-1 agonist	NCT03596450	Multiple countries
Garvey, W.T. (2023) (SURMOUNT-2)[322]	patients with type 2 diabetes mellitus and obesity	Tirzepatide 10mg/week<br>Tirzepatide 15mg/week<br>Placebo	312<br>311<br>315	54.3±10.7<br>53.6±10.6<br>54.7±10.5	50.6<br>51.1<br>50.5	72 weeks	injection	GLP-1 agonist	NCT04657003	Multiple countries
Herrington, W.G. (2023) (EMPA-KIDNEY)[323]	patients with renal failure	Empagliflozin 10mg/day<br>Placebo	3304<br>3305	63.9±13.9<br>63.8±13.9	33.2<br>33.1	104 weeks	oral	SGLT2 inhibitor	NCT03594110	Multiple countries
Solomon, S.D. (2022) (DELIVER)[324]	patients with stabilized heart failure	Dapagliflozin 10mg/day<br>Placebo	3131<br>3132	71.8±9.6<br>71.5±9.5	43.6<br>44.2	120 weeks	oral	SGLT2 inhibitor	NCT03619213	Multiple countries
Tuttle, K.R. (2022) (NCT01011868)[325]	patients with type 2 diabetes mellitus and	Empagliflozin 10mg/day<br>Empagliflozin	169<br>155<br>170	58.6±9.8<br>59.9±10.5<br>58.1±9.4	45.0<br>40.0<br>47.1	78 weeks	oral	SGLT2 inhibitor	NCT01011868	Multiple countries

	chronic kidney disease	25mg/day<br>Placebo								
Wada, T. (2022) (TA-7284-14)[326]	patients with type 2 diabetes mellitus and chronic kidney disease	Canagliflozin 100 mg/day<br>Placebo	154<br>154	62.5±10.5<br>62.4±11.1	25.3<br>16.2	104 weeks	oral	SGLT2 inhibitor	NCT03436693	Japan
Anker, S.D. (2021) (EMPEROR-Preserved)[327]	patients with heart failure with preserved ejection fraction	Empagliflozin 10mg/day<br>Placebo	2997<br>2991	71.8±9.3<br>71.9±9.6	44.6<br>44.7	156 weeks	oral	SGLT2 inhibitor	NCT03057951	Multiple countries
Bhatt, D.L. (2021) (SCORED)[328]	patients with type 2 diabetes mellitus and chronic kidney disease	Sotagliflozin 200-400mg/day<br>Placebo	5292<br>5292	68.4±8.4<br>68.2±8.4	44.3<br>45.5	116 weeks	oral	SGLT2 inhibitor	NCT03315143	Multiple countries
Davies, M. (2021) (STEP 2)[329]	patients with type 2 diabetes mellitus and obesity	Inject semaglutide 1.0 mg/week<br>Inject Semaglutide 2.4 mg/week<br>Placebo	403<br>404<br>403	56.0±10.0<br>55.0±11.0<br>55.0±11.0	50.4<br>55.2<br>47.1	68 weeks	injection	GLP-1 agonist	NCT03552757	Multiple countries
Lock, J.P. (2021) (BEST) (NCT02558296)[330]	patients with type 2 diabetes mellitus	Bexagliflozin 20mg/day<br>Placebo	1132<br>567	64.4±7.9<br>64.6±8.0	30.1<br>31.2	52 weeks	oral	SGLT2 inhibitor	NCT02558296	Multiple countries

Ludvik, B. (2021) (SURPASS-3)[331]	patients with type 2 diabetes mellitus	Tirzepatide 5mg/week<br>Tirzepatide 10mg/week<br>Tirzepatide 15mg/week<br>Control	358<br>360<br>359<br>360	57.2±10.1<br>57.4±9.7<br>57.5±10.2<br>57.5±10.1	44.1<br>45.8<br>46.0<br>40.8	67 weeks	injection	GLP-1 agonist	NCT03882970	Multiple countries
Cannon, C.P. (2020) (VERTIS CV)[332]	patients with type 2 diabetes mellitus	Ertugliflozin 5 mg/day<br>Ertugliflozin 15 mg/day<br>Placebo	2752<br>2747<br>2747	64.3±8.2<br>64.4±8.0<br>64.4±8.0	29.1<br>30.3<br>30.7	182 weeks	oral	SGLT2 inhibitor	NCT01986881	Multiple countries
Heerspink, H.J.L. (2020) (DAPA-CKD)[333]	patients with renal failure	Dapagliflozin 10mg/day<br>Placebo	2152<br>2152	61.8±12.1<br>61.9±12.1	32.9<br>33.3	125 weeks	oral	SGLT2 inhibitor	NCT03036150	Multiple countries
Packer, M. (2020) (EMPEROR-Reduced)[334]	patients with chronic heart failure	Empagliflozin 10mg/day<br>Placebo	1863<br>1867	67.2±10.8<br>66.5±11.2	23.5<br>24.4	64 weeks	oral	SGLT2 inhibitor	NCT03057977	Multiple countries
Husain, M. (2019) (PIONEER 6)[335]	patients withtype 2 diabetes mellitus and cardiovascular disease or chronic kidney disease	Oral semaglutide 14mg/day<br>Placebo	1591<br>1592	66.0±7.0<br>66.0±7.0	31.9<br>31.4	64 weeks	oral	GLP-1 agonist	NCT02692716	Multiple countries
Ledesma, G. (2019) (1218.149)[336]	patients with type 2 diabetes mellitus	Linagliptin 5 mg/day<br>Placebo	151<br>151	72.3±5.1<br>72.5±5.6	39.1<br>39.7	52 weeks	oral	DPP4 inhibitor	NCT02240680	Multiple countries
Matthews, D.R. (2019) (VERIFY)[337]	patients with type 2	Vildagliptin 100mg/day<br>Placebo	998<br>1003	54.1±9.5<br>54.6±9.2	54.6<br>51.3	260 weeks	oral	DPP4 inhibitor	NCT01528254	Multiple countries

	diabetes mellitus									
McMurray, J.J.V. (2019) (DAPA-HF)[338]	patients with stabilized heart failure	Dapagliflozin 10mg/day<br>Placebo	2373<br>2371	66.2±11.0<br>66.5±10.8	23.8<br>23.0	73 weeks	oral	SGLT2 inhibitor	NCT03036124	Multiple countries
Perkovic, V. (2019) (CREDENCE)[339]	patients with type 2 diabetes mellitus and nephropathy	Canagliflozin 100 mg/day<br>Placebo	2202<br>2199	62.9±9.2<br>63.2±9.2	34.6<br>33.3	130 weeks	oral	SGLT2 inhibitor	NCT02065791	Multiple countries
Rosenstock, J. (2019) (CAROLINA)[340]	patients with type 2 diabetes mellitus	Linagliptin 5 mg/day<br>Placebo	3023<br>3010	63.9±9.5<br>64.2±9.5	39.2<br>40.8	338 weeks	oral	DPP4 inhibitor	NCT01243424	Multiple countries
Rosenstock, J. (2019) (PIONEER 3)[341]	patients with type 2 diabetes mellitus	Oral semaglutide 3 mg/day<br>Oral semaglutide 7 mg/day<br>Oral semaglutide 14 mg/day<br>Sitagliptin 100 mg/day	466<br>465<br>465<br>467	58.0±10.0<br>58.0±10.0<br>57.0±10.0<br>58.0±10.0	45.5<br>47.3<br>46.9<br>49.0	78 weeks	oral	GLP-1 agonist	NCT02607865	Multiple countries
Wiviott, S.D. (2019) (DECLARE-TIMI 58)[342]	patients with type 2 diabetes mellitus and atherosclerotic vascular disease	Dapagliflozin 10mg/day<br>Placebo	8582<br>8578	63.9±6.8<br>64.0±6.8	36.9<br>37.9	206 weeks	oral	SGLT2 inhibitor	NCT01730534	Multiple countries

Chen, Y. (2018) (SUPER)[343]	patients with type 2 diabetes mellitus	Saxagliptin 5mg/day<br>Placebo	232<br>230	59.3±7.9<br>58.9±8.2	53.0<br>56.5	24 weeks	oral	DPP4 inhibitor	NCT02104804	China
Kaku, K. (2018) (SUSTAIN)[344]	patients with type 2 diabetes mellitus	Inject semaglutide 0.5 mg/week<br>Inject semaglutide 1.0 mg/week<br>Control	239<br>241<br>121	58.0±10.6<br>58.7±10.2<br>59.2±10.1	30.5<br>27.8<br>25.8	61 weeks	injection	GLP-1 agonist	NCT02207374	Multiple countries
Tuttle, K. R. (2018) (AWARD-7)[345]	patients with type 2 diabetes mellitus and moderate-to-severe chronic kidney disease	Dulaglutide 0.75 mg/week<br>Dulaglutide 1.5 mg/week<br>Control	190<br>192<br>194	64.7±8.6<br>64.7±8.8<br>64.3±8.4	44.7<br>45.8<br>52.1	52 weeks	injection	GLP-1 agonist	NCT01621178	Multiple countries
Groop, P.H. (2017) (MARLINA-T2D)[346]	patients with type 2 diabetes mellitus	Linagliptin 5 mg/day<br>Placebo	182<br>178	61.0±10.0<br>60.1±9.3	36.3<br>36.5	24 weeks	oral	DPP4 inhibitor	NCT01792518	Multiple countries
Holman, R.R. (2017) (EXSCEL)[347]	patients with type 2 diabetes mellitus	Exenatide 2mg/day<br>Placebo	7356<br>7396	61.8±9.4<br>61.9±9.4	38.0<br>38.0	166 weeks	injection	GLP-1 agonist	NCT01144338	Multiple countries
Neal, B. (2017) (CANVAS)[348]	patients with type 2 diabetes mellitus	Canagliflozin 100 mg/day<br>Canagliflozin 300 mg/day<br>Placebo	1445<br>1443<br>1442	62.2±8.0<br>62.8±8.1<br>62.3±7.9	33.5<br>34.6<br>33.7	126 weeks	oral	SGLT2 inhibitor	NCT01032629	Multiple countries

Neal, B. (2017) (CANVAS-R)[349]	patients with type 2 diabetes mellitus	Canagliflozin 300 mg/day<br>Placebo	2907<br>2905	63.9±8.4<br>64.0±8.3	36.2<br>38.2	126 weeks oral	SGLT2 inhibitor	NCT01989754	Multiple countries
Del Prato, S. (2016) (ENDURE)[350]	patients with type 2 diabetes mellitus	Alogliptin 12.5 mg/day<br>Alogliptin 25 mg/day<br>Placebo	880<br>885<br>874	55.2±9.6<br>55.5±9.8<br>55.4±9.6	52.4<br>48.9<br>49.5	104 weeks oral	DPP4 inhibitor	NCT00856284	Multiple countries
Marso, S.P. (2016) (LEADER)[351]	patients with type 2 diabetes mellitus	Liraglutide 1.8mg/day<br>Placebo	4668<br>4672	64.2±7.2<br>64.4±7.2	35.5<br>36.0	198 weeks injection	GLP-1 agonist	NCT01179048	Multiple countries
Mellander, A. (2016) (NCT00528372)[124]	patients with type 2 diabetes mellitus	Dapagliflozin 2.5 mg/day<br>Dapagliflozin 5 mg/day<br>Dapagliflozin 10 mg/day<br>Placebo	132<br>132<br>146<br>75	NA	50.7<br>54.6<br>50.0<br>58.7	102 weeks oral	SGLT2 inhibitor	NCT00528372	Multiple countries
Wang, W. (2016)[352]	patients with type 2 diabetes mellitus	Linagliptin 5 mg/day<br>Placebo	205<br>100	55.1±10.7<br>56.5±8.7	50.2<br>50.0	24 weeks oral	DPP4 inhibitor	NCT01215097	Multiple countries
DeFronzo, R.A. (2015) (1275.1)[353]	patients with type 2 diabetes mellitus	Empagliflozin 25 mg/day + Linagliptin 5 mg/day<br>Empagliflozin 10 mg/day + Linagliptin 5 mg/day<br>Empagliflozin	134<br>135<br>140<br>137<br>128	57.1±10.2<br>56.2±10.3<br>55.5±10.0<br>56.1±10.5<br>56.2±10.0	46.3<br>38.5<br>53.6<br>43.1<br>50.0	52 weeks oral	DPP4 inhibitor/SGLT2 inhibitor	NCT01422876	Multiple countries

		25 mg/day<br>Empagliflozin 10 mg/day<br>Linagliptin 5 mg/day								
Green, J.B. (2015) (TECOS - MK-0431-082)[354]	patients with type 2 diabetes mellitus	Sitagliptin 100 mg/day<br>Placebo	7332<br>7339	65.4±7.9<br>65.5±8.0	29.1<br>29.5	192 weeks	oral	DPP4 inhibitor	NCT00790205	Multiple countries
Roden, M. (2015) (EMPA-REG EXTEND MONO)[355]	patients with type 2 diabetes mellitus	Empagliflozin 10mg/day<br>Empagliflozin 25mg/day<br>Sitagliptin 100 mg/day<br>Placebo	224<br>224<br>223<br>228	56.2±11.6<br>53.8±11.6<br>55.1±9.9<br>54.9±10.9	36.6<br>35.3<br>36.8<br>46.1	76 weeks	oral	DPP4 inhibitor/SGLT2 inhibitor	NCT01289990	Multiple countries
Rosenstock, J. (2015) (CV181-169)[356]	patients with type 2 diabetes mellitus	Saxagliptin 5mg/day<br>Dapagliflozin 10mg/day<br>Saxagliptin 5mg/day + Dapagliflozin 10mg/day	176<br>179<br>179	55.0±10.0<br>54.0±10.0<br>53.0±10.0	46.6<br>50.3<br>52.5	24 weeks	oral	DPP4 inhibitor/SGLT2 inhibitor	NCT01606007	Multiple countries
Zinman, B. (2015) (EMPA-REG OUTCOME)[357]	patients with type 2 diabetes mellitus	Empagliflozin 10mg/day<br>Empagliflozin 25mg/day<br>Placebo	2345<br>2342<br>2333	63.0±8.6<br>63.2±8.6<br>63.2±8.8	29.5<br>28.1<br>28.0	135 weeks	oral	SGLT2 inhibitor	NCT01131676	Multiple countries

Wysham, C. (2014) (AWARD-1)[358]	patients with type 2 diabetes mellitus	Dulaglutide 0.75 mg/week<br>Dulaglutide 1.5 mg/week<br>Exenatide<br>Placebo	280<br>279<br>276<br>141	55.8±9.5<br>56.3±9.7<br>55.0±10.0<br>55.0±10.0	40.0<br>41.6<br>43.5<br>41.1	26 weeks	injection	GLP-1 agonist	NCT01064687	Multiple countries
Lavalle-Gonzalez, F.J. (2013) (data before week 26) (CANTATA-D)[359]	patients with type 2 diabetes mellitus	Canagliflozin 100 mg/day<br>Canagliflozin 300 mg/day<br>Placebo<br>Sitagliptin 100 mg/day	368<br>367<br>183<br>366	55.5±9.4<br>55.3±9.2<br>55.3±9.8	52.7<br>55.0<br>48.6	26 weeks	oral	SGLT2 inhibitor	NCT01106677	Multiple countries
Rosenstock, J. (2013) (GetGoal-X)[360]	patients with type 2 diabetes mellitus	Lixisenatide 20ug/day<br>Exenatide	318<br>316	57.3±9.2<br>57.6±10.7	52.5<br>40.8	24 weeks	injection	GLP-1 agonist	NCT00707031	Multiple countries
White, W.B. (2013) (EXAMINE)[361]	patients with type 2 diabetes mellitus	Alogliptin 25 mg/day<br>Placebo	2701<br>2679	61.0<br>61.0	32.3<br>32.0	52 weeks	oral	DPP4 inhibitor	NCT00968708	Multiple countries
Yki-Järvinen, H. (2013) (2008-008296-33)[362]	patients with type 2 diabetes mellitus	Linagliptin 5 mg/day<br>Placebo	631<br>630	59.7±9.9<br>60.4±10.0	47.9<br>47.8	52 weeks	oral	DPP4 inhibitor	NCT00954447	Multiple countries
Kawamori, R. (2012) (Japanese P III) (1st stage)[363]	patients with type 2 diabetes mellitus	Linagliptin 5 mg/day<br>Linagliptin 10 mg/day<br>Placebo	159<br>160<br>80	60.3±9.4<br>61.3±10.0<br>59.7±8.9	30.2<br>30.0<br>28.7	12 weeks	oral	DPP4 inhibitor	NCT00654381	Japan

Russell-Jones, D. (2012) (DURATION-4)[364]	patients with type 2 diabetes mellitus	Exenatide 2 mg/day<br>Sitagliptin 100 mg/day<br>Placebo	248<br>163<br>246	53.7±10.9<br>52.3±11.1<br>53.7±11.1	44.0<br>42.3<br>37.4	26 weeks	oral/injection	DPP4 inhibitor/GLP-1 receptor agonist	NCT00676338	Multiple countries
Seino, Y. (2012) (NCT01318135)[186]	patients with type 2 diabetes mellitus	Alogliptin 12.5 mg/day<br>Alogliptin 25 mg/day	292<br>297	NA	33.9<br>32.3	40 weeks	oral	DPP4 inhibitor	NCT01318135	Japan
Chacra, A.R. (2009) (CV181-040)[365]	patients with type 2 diabetes mellitus	Saxagliptin 2.5mg/day<br>Saxagliptin 5mg/day<br>Placebo	248<br>253<br>267	55.4±9.6<br>54.9±10.0<br>55.1±10.7	54.4<br>56.5<br>53.9	24 weeks	oral	DPP4 inhibitor	NCT00313313	Multiple countries
DeFronzo, R.A. (2009) (CV181-014)[366]	patients with type 2 diabetes mellitus	Saxagliptin 2.5mg/day<br>Saxagliptin 5mg/day<br>Saxagliptin 10mg/day<br>Placebo	192<br>191<br>181<br>179	54.7±10.1<br>54.7±9.6<br>54.2±10.1<br>54.8±10.2	56.8<br>46.1<br>47.5<br>46.4	24 weeks	oral	DPP4 inhibitor	NCT00121667	Multiple countries
Mohan, V. (2009) (MK0431-040)[367]	patients with type 2 diabetes mellitus	Sitagliptin 100 mg/day<br>Placebo	352<br>178	50.9±9.3<br>50.9±9.3	43.2<br>40.4	18 weeks	oral	DPP4 inhibitor	NCT00289848	Multiple countries
Nauck, M.A. (2007) (MK0431-024)[368]	patients with type 2 diabetes mellitus	Sitagliptin 100 mg/day<br>Placebo	588<br>584	56.8±9.3<br>56.6±9.8	42.9<br>38.7	52 weeks	oral	DPP4 inhibitor	NCT00094770	Multiple countries

Abbreviations: DPP4 inhibitor: dipeptidyl peptidase 4 inhibitor; GLP-1 agonist: glucagon-like peptide-1 agonist; NA: not available; SGLT2 inhibitor: sodium–glucose cotransporter 2 inhibitor

Table S6A: League table of NMA of primary outcome: gastric tumor risk – study duration at least 52 weeks

GLP1RA	***1.94 (1.05,3.59)**	***2.12 (1.03,4.35)**	2.57 (0.37,17.93)	6.14 (0.45,84.44)	***3.99 (1.77,8.97)**
***0.52 (0.28,0.95)**	Placebo_or_Control	1.09 (0.75,1.58)	1.32 (0.21,8.36)	3.16 (0.25,40.51)	***2.05 (1.19,3.55)**
***0.47 (0.23,0.97)**	0.92 (0.63,1.33)	SGLT2i	1.21 (0.18,7.95)	2.90 (0.23,37.10)	1.88 (0.98,3.62)
0.39 (0.06,2.72)	0.76 (0.12,4.79)	0.83 (0.13,5.42)	DualRA	2.39 (0.10,55.71)	1.55 (0.23,10.65)
0.16 (0.01,2.24)	0.32 (0.02,4.05)	0.34 (0.03,4.41)	0.42 (0.02,9.72)	SGLT2i_DPP4i	0.65 (0.05,8.31)
***0.25 (0.11,0.56)**	***0.49 (0.28,0.84)**	0.53 (0.28,1.02)	0.64 (0.09,4.41)	1.54 (0.12,19.70)	DPP4i

Data presents RR [95%CIs]. Network meta-analysis results are presented as estimate effect sizes for the outcome of gastric tumor risk. Interventions are reported in order of mean ranking of beneficially prophylactic effect on gastric tumor risk, and outcomes are expressed as risk ratio (RR) (95% confidence intervals) (95%CIs). For the upper-right portion, RR of less than 1 indicates that the treatment specified in the row got more beneficial effect than that specified in the column. For the lower-left portion, RR of less than 1 indicates that the treatment specified in the column has more beneficial effect than that specified in the row. Bold results marked with * indicate statistical significance.

Table S6B: League table of NMA of primary outcome: gastric tumor risk – study duration less than 52 weeks

SGLT2i	1.50 (0.08,28.83)	3.24 (0.42,25.06)	3.39 (0.48,24.19)	5.35 (0.35,81.56)
0.67 (0.03,12.76)	GLP1RA	2.16 (0.20,23.59)	2.26 (0.24,20.94)	3.56 (0.10,123.09)
0.31 (0.04,2.39)	0.46 (0.04,5.08)	DPP4i	1.05 (0.38,2.89)	1.65 (0.11,25.17)
0.29 (0.04,2.10)	0.44 (0.05,4.11)	0.95 (0.35,2.64)	Placebo_or_Control	1.58 (0.10,25.61)
0.19 (0.01,2.84)	0.28 (0.01,9.70)	0.61 (0.04,9.22)	0.63 (0.04,10.29)	SGLT2i_DPP4i

Data presents RR [95%CIs]. Network meta-analysis results are presented as estimate effect sizes for the outcome of gastric tumor risk. Interventions are reported in order of mean ranking of beneficially prophylactic effect on gastric tumor risk, and outcomes are expressed as risk ratio (RR) (95% confidence intervals) (95%CIs). For the upper-right portion, RR of less than 1 indicates that the treatment specified in the row got more beneficial effect than that specified in the column. For the lower-left portion, RR of less than 1 indicates that the treatment specified in the column has more beneficial effect than that specified in the row. Bold results marked with * indicate statistical significance.

Table S6C: League table of NMA of primary outcome: gastric tumor risk – at least 60 years old

GLP1RA	1.97 (0.98,3.97)	2.18 (0.98,4.83)	***4.10 (1.64,10.29)**
0.51 (0.25,1.02)	Placebo_or_Control	1.10 (0.76,1.61)	***2.08 (1.15,3.77)**
0.46 (0.21,1.02)	0.91 (0.62,1.32)	SGLT2i	1.88 (0.93,3.81)
***0.24 (0.10,0.61)**	***0.48 (0.27,0.87)**	0.53 (0.26,1.07)	DPP4i

Data presents RR [95%CIs]. Network meta-analysis results are presented as estimate effect sizes for the outcome of gastric tumor risk. Interventions are reported in order of mean ranking of beneficially prophylactic effect on gastric tumor risk, and outcomes are expressed as risk ratio (RR) (95% confidence intervals) (95%CIs). For the upper-right portion, RR of less than 1 indicates that the treatment specified in the row got more beneficial effect than that specified in the column. For the lower-left portion, RR of less than 1 indicates that the treatment specified in the column has more beneficial effect than that specified in the row. Bold results marked with * indicate statistical significance.

Table S6D: League table of NMA of primary outcome: gastric tumor risk – less than 60 years old

SGLT2i	1.16 (0.18,7.64)	2.40 (0.51,11.34)	2.72 (0.57,13.00)	3.17 (0.28,35.35)	4.14 (0.59,29.07)
0.86 (0.13,5.70)	GLP1RA	2.07 (0.68,6.34)	2.35 (0.63,8.83)	2.74 (0.32,23.68)	3.57 (0.36,35.22)
0.42 (0.09,1.97)	0.48 (0.16,1.48)	Placebo_or_Control	1.13 (0.49,2.65)	1.32 (0.21,8.36)	1.72 (0.23,13.21)
0.37 (0.08,1.76)	0.43 (0.11,1.60)	0.88 (0.38,2.06)	DPP4i	1.16 (0.15,8.87)	1.52 (0.22,10.69)
0.32 (0.03,3.52)	0.37 (0.04,3.16)	0.76 (0.12,4.79)	0.86 (0.11,6.54)	DualRA	1.31 (0.08,20.36)
0.24 (0.03,1.70)	0.28 (0.03,2.76)	0.58 (0.08,4.44)	0.66 (0.09,4.62)	0.77 (0.05,11.95)	SGLT2i_DPP4i

Data presents RR [95%CIs]. Network meta-analysis results are presented as estimate effect sizes for the outcome of gastric tumor risk. Interventions are reported in order of mean ranking of beneficially prophylactic effect on gastric tumor risk, and outcomes are expressed as risk ratio (RR) (95% confidence intervals) (95%CIs). For the upper-right portion, RR of less than 1 indicates that the treatment specified in the row got more beneficial effect than that specified in the column. For the lower-left portion, RR of less than 1 indicates that the treatment specified in the column has more beneficial effect than that specified in the row. Bold results marked with * indicate statistical significance.

Table S6E: League table of NMA of secondary outcome: *Helicobacter pylori* risk

DPP4i	1.23 (0.25,6.14)	1.30 (0.39,4.35)	2.95 (0.29,29.60)	2.96 (0.39,22.52)
0.81 (0.16,4.04)	SGLT2i	1.05 (0.37,3.03)	2.39 (0.26,22.27)	2.40 (0.34,16.76)
0.77 (0.23,2.58)	0.95 (0.33,2.73)	Placebo_or_Control	2.27 (0.32,16.19)	2.27 (0.44,11.64)
0.34 (0.03,3.40)	0.42 (0.04,3.89)	0.44 (0.06,3.14)	DualRA	1.00 (0.12,8.21)
0.34 (0.04,2.58)	0.42 (0.06,2.91)	0.44 (0.09,2.25)	1.00 (0.12,8.18)	GLP1RA

Data presents RR [95%CIs]. Network meta-analysis results are presented as estimate effect sizes for the outcome of *Helicobacter pylori* risk. Interventions are reported in order of mean ranking of beneficially prophylactic effect on *Helicobacter pylori* risk, and outcomes are expressed as risk ratio (RR) (95% confidence intervals) (95%CIs). For the upper-right portion, RR of less than 1 indicates that the treatment specified in the row got more beneficial effect than that specified in the column. For the lower-left portion, RR of less than 1 indicates that the treatment specified in the column has more beneficial effect than that specified in the row. Bold results marked with * indicate statistical significance.

Table S6F: League table of NMA of acceptability: drop-out rate

SGLT2i_DPP4i	1.60 (0.94,2.71)	1.62 (0.95,2.74)	1.64 (0.91,2.96)	***1.76 (1.02,3.03)**	***1.87 (1.10,3.17)**
0.63 (0.37,1.06)	DPP4i	1.01 (0.92,1.12)	1.03 (0.78,1.35)	1.10 (0.95,1.28)	***1.17 (1.08,1.27)**
0.62 (0.36,1.05)	0.99 (0.89,1.09)	SGLT2i	1.01 (0.77,1.33)	1.09 (0.94,1.26)	***1.16 (1.07,1.24)**
0.61 (0.34,1.10)	0.97 (0.74,1.28)	0.99 (0.75,1.30)	DualRA	1.07 (0.80,1.44)	1.14 (0.88,1.48)
***0.57 (0.33,0.98)**	0.91 (0.78,1.05)	0.92 (0.79,1.07)	0.93 (0.70,1.25)	GLP1RA	1.06 (0.94,1.21)
***0.53 (0.32,0.91)**	***0.85 (0.79,0.93)**	***0.87 (0.80,0.93)**	0.88 (0.67,1.14)	0.94 (0.83,1.07)	Placebo_or_Control

Data presents RR [95%CIs]. Network meta-analysis results are presented as estimate effect sizes for the outcome of drop-out rate. Interventions are reported in order of mean ranking of acceptability, and outcomes are expressed as risk ratio (RR) (95% confidence intervals) (95%CIs). For the upper-right portion, RR of less than 1 indicates that the treatment specified in the row got better acceptability than that specified in the column. For the lower-left portion, RR of less than 1 indicates that the treatment specified in the column has better acceptability than that specified in the row. Bold results marked with * indicate statistical significance.

Abbreviation: 95%CIs: 95% confidence intervals; DPP4 inhibitor: dipeptidyl-peptidase 4 inhibitor; GLP-1 agonist: glucagon-like peptide-1 agonist; NMA: network meta-analysis; RR: risk ratio; RCT: randomized controlled trial; SGLT2 inhibitor: sodium–glucose cotransporter 2 inhibitor

Table S7A SUCRA for primary outcome: gastric tumor risk – class level

Treatment	SUCRA	PrBest	MeanRank
GLP1RA	95.5	80.3	1.2
Placebo_or_Control	62.2	0.2	2.9
SGLT2i	57.9	1.2	3.1
DualRA	45.8	15.4	3.7
DPP4i	24.2	0.1	4.8
SGLT2i_DPP4i	14.5	2.8	5.3

Table S7B SUCRA for primary outcome: gastric tumor risk – regimen level

Treatment	SUCRA	PrBest	MeanRank
Oral_semaglutide	81.6	28.3	4.7
Liraglutide	80.9	10.5	4.8
Exenatide	79.0	3.6	5.2
Inject_semaglutide	69.0	5.7	7.2
Empagliflozin	57.7	0.1	9.5
Placebo_or_Control	55.5	0.0	9.9
Bexagliflozin	55.2	8.1	10.0
Dapagliflozin	55.2	0.0	10.0
Dulaglutide	55.2	5.8	10.0
Sotagliflozin	54.8	5.2	10.0
Vildagliptin	53.8	11.6	10.2
Saxagliptin	52.5	2.9	10.5
Canagliflozin	49.0	0.0	11.2
Lixisenatide	46.6	10.1	11.7
Tirzepatide	45.7	3.8	11.9
Sitagliptin	32.8	0.0	14.4
Linagliptin	31.9	0.0	14.6
Dapagliflozin_Saxagliptin	29.2	1.7	15.2
Empagliflozin_Linagliptin	29.2	2.1	15.2

Alogliptin	23.9	0.2	16.2
Ertugliflozin	11.3	0.3	18.7

Table S7C SUCRA for primary outcome: gastric tumor risk – focus participants with diabetes mellitus

Treatment	SUCRA	PrBest	MeanRank
GLP1RA	93.6	72.5	1.3
SGLT2i	63.5	2.9	2.8
Placebo_or_Control	56.6	0.2	3.2
DualRA	47.4	21.4	3.6
DPP4i	23.3	0.1	4.8
SGLT2i_DPP4i	15.5	2.9	5.2

Table S7D SUCRA for primary outcome: gastric tumor risk – study duration at least 52 weeks

Treatment	SUCRA	PrBest	MeanRank
GLP1RA	94.3	75.6	1.3
Placebo_or_Control	61.8	0.1	2.9
SGLT2i	54.3	1.0	3.3
DualRA	46.3	15.0	3.7
SGLT2i_DPP4i	23.3	8.2	4.8
DPP4i	20.0	0.1	5.0

Table S7E SUCRA for primary outcome: gastric tumor risk – study duration less than 52 weeks

Treatment	SUCRA	PrBest	MeanRank
SGLT2i	81.4	53.5	1.7
GLP1RA	65.1	35.6	2.4
DPP4i	40.1	3.1	3.4
Placebo_or_Control	36.2	1.0	3.6
SGLT2i_DPP4i	27.2	6.8	3.9

Table S7F SUCRA for primary outcome: gastric tumor risk – at least 60 years old

Treatment	SUCRA	PrBest	MeanRank
GLP1RA	98.1	96.2	1.1
Placebo_or_Control	56.5	1.5	2.3
SGLT2i	44.3	2.3	2.7
DPP4i	1.1	0.0	4.0

Table S7G SUCRA for primary outcome: gastric tumor risk – less than 60 years old

Treatment	SUCRA	PrBest	MeanRank
SGLT2i	82.2	50.3	1.9
GLP1RA	78.9	37.0	2.1
Placebo_or_Control	42.8	0.3	3.9
DPP4i	36.5	0.9	4.2
DualRA	33.6	7.6	4.3
SGLT2i_DPP4i	26.0	3.9	4.7

Table S7H SUCRA for acceptability: drop-out rate

Treatment	SUCRA	PrBest	MeanRank
SGLT2i_DPP4i	96.7	92.4	1.2
DPP4i	61.5	1.5	2.9
SGLT2i	57.6	1.2	3.1
DualRA	49.0	4.7	3.5
GLP1RA	28.7	0.2	4.6
Placebo_or_Control	6.5	0.0	5.7

Abbreviation: 95%CIs: 95% confidence intervals; DPP4 inhibitor: dipeptidyl-peptidase 4 inhibitor; GLP-1 agonist: glucagon-like peptide-1 agonist; NMA: network meta-analysis; RR: risk ratio; RCT: randomized controlled trial; SGLT2 inhibitor: sodium–glucose cotransporter 2 inhibitor

Table S8 Heterogeneity for primary outcome: gastric tumor risk

	Heterogeneity statistic	degrees of freedom	*p*	*I squared*	*Tau-squared*	Treatments used	
D - A	15.83	19	0.668	0.00%	0	A:	Placebo_or_Control
B - A	0.88	8	0.999	0.00%	0	B:	GLP1RA
E - A	4.54	17	0.999	0.00%	0	C:	DualRA
E - D	0.51	3	0.916	0.00%	0	D:	SGLT2i
E - B	0.47	1	0.491	0.00%	0	E:	DPP4i
C - A	0.04	2	0.979	0.00%	0	F:	SGLT2i_DPP4i
F - D	0	1	0.996	0.00%	0		
F - E	0.09	1	0.763	0.00%	0		

Abbreviation: 95%CIs: 95% confidence intervals; DPP4 inhibitor: dipeptidyl-peptidase 4 inhibitor; GLP-1 agonist: glucagon-like peptide-1 agonist; NMA: network meta-analysis; RR: risk ratio; RCT: randomized controlled trial; SGLT2 inhibitor: sodium–glucose cotransporter 2 inhibitor

Table S9A Side-splitting model inconsistency for primary outcome: gastric tumor risk

Side	Direct		Indirect		Difference			tau		
	Coef.	Std. Err.	Coef.	Std. Err.	Coef.	Std. Err.	P>z		Treatments used	
A B	-0.663138	0.3078802	-0.9485768	1.481474	0.2854388	1.506676	0.85	9.10E-06	A:	Placebo_or_Control
A C	.	.	.	.	.	.	.	.	B:	GLP1RA
A D	0.03204	0.1878562	0.6703223	1.163062	-0.6382823	1.172573	0.586	4.71E-07	C:	DualRA
A E	0.5087985	0.2523739	1.474418	0.9431071	-0.9656197	0.9709345	0.32	1.96E-06	D:	SGLT2i
B E	1.481535	1.263865	1.218406	0.4015266	0.2631288	1.32611	0.843	1.42E-06	E:	DPP4i
D E *	0.741505	0.9204074	0.4961487	0.3210475	0.2453563	0.9775789	0.802	5.49E-07	F:	SGLT2i_DPP4i
D F *	1.104199	1.153101	1.426615	2.388144	-0.322416	2.892442	0.911	9.14E-07		
E F *	0.7347187	1.152883	0.4123024	2.38846	0.3224162	2.892442	0.911	2.55E-07		

Table S9B Design-by-treatment model and loop inconsistency for primary outcome: gastric tumor risk

Inconsistency model	chi^2	*p* value of Prob>chi^2
	Primary outcome: gastric tumor risk	
design-by-treatment	5.13	0.5275
loop inconsistency	0.37	0.8321

Abbreviation: 95%CIs: 95% confidence intervals; DPP4 inhibitor: dipeptidyl-peptidase 4 inhibitor; GLP-1 agonist: glucagon-like peptide-1 agonist; NMA: network meta-analysis; RR: risk ratio; RCT: randomized controlled trial; SGLT2 inhibitor: sodium–glucose cotransporter 2 inhibitor

Table S10 GRADE for primary outcome: gastric tumor risk

	Comparison	Study limitations	Imprecision	Inconsistency	Indirectness	Publication bias	GRADE
1	DPP4i:DualRA	No downgrade	Downgrade because the opposite limit exceed	No downgrade	No downgrade	No downgrade	LOW
2	DPP4i:GLP1RA	No downgrade	Upgrade due to large effect size	No downgrade	No downgrade	No downgrade	HIGH
3	DPP4i:Placebo_or_Control	No downgrade	Upgrade due to large effect size	No downgrade	No downgrade	No downgrade	HIGH
4	DPP4i:SGLT2i	No downgrade	No downgrade	No downgrade	No downgrade	No downgrade	MODERATE
5	DPP4i:SGLT2i_DPP4i	No downgrade	No downgrade	No downgrade	No downgrade	No downgrade	MODERATE
6	DualRA:GLP1RA	No downgrade	No downgrade	No downgrade	No downgrade	No downgrade	MODERATE
7	DualRA:Placebo_or_Control	Downgrade because risk of bias	No downgrade	No downgrade	No downgrade	No downgrade	LOW
8	DualRA:SGLT2i	No downgrade	Downgrade because the opposite limit exceed	No downgrade	No downgrade	No downgrade	LOW
9	DualRA:SGLT2i_DPP4i	No downgrade	Downgrade because the opposite limit exceed	No downgrade	No downgrade	No downgrade	LOW
10	GLP1RA:Placebo_or_Control	No downgrade	Upgrade due to large effect size	No downgrade	No downgrade	No downgrade	HIGH
11	GLP1RA:SGLT2i	No downgrade	Upgrade due to large effect size	No downgrade	No downgrade	No downgrade	HIGH
12	GLP1RA:SGLT2i_DPP4i	No downgrade	No downgrade	No downgrade	No downgrade	No downgrade	MODERATE
13	SGLT2i:Placebo_or_Control	Downgrade because risk of bias	No downgrade	No downgrade	No downgrade	No downgrade	LOW
14	SGLT2i_DPP4i:Placebo_or_Control	No downgrade	No downgrade	No downgrade	No downgrade	No downgrade	MODERATE
15	SGLT2i:SGLT2i_DPP4i	No downgrade	No downgrade	No downgrade	No downgrade	No downgrade	MODERATE

Abbreviation: 95%CIs: 95% confidence intervals; DPP4 inhibitor: dipeptidyl-peptidase 4 inhibitor; GLP-1 agonist: glucagon-like peptide-1 agonist; NMA: network meta-analysis; RR: risk ratio; RCT: randomized controlled trial; SGLT2 inhibitor: sodium–glucose cotransporter 2 inhibitor

Reference list of supplement tables:

1. Page, M.J.; McKenzie, J.E.; Bossuyt, P.M.; Boutron, I.; Hoffmann, T.C.; Mulrow, C.D.; Shamseer, L.; Tetzlaff, J.M.; Akl, E.A.; Brennan, S.E.; et al. The PRISMA 2020 statement: an updated guideline for reporting systematic reviews. *Bmj* **2021**, *372*, n71, doi:10.1136/bmj.n71.
2. Arslan, H.E.; Teksen, Y.; Ozatik, O.; Algin, M.C. The effect of liraglutide, a GLP-1 analog, on indomethacin-induced gastric ulcers in diabetic rats. *Acta Cir Bras* **2025**, *40*, e407325, doi:10.1590/acb407325.
3. Hajishah, H.; Mazloom, P.; Salehi, A.; Kazemi, D.; Samiee, R.; Majlesi, H.; Amini, M.J.; Meyari, A.; Dehnavi, N.S.; Zangi, M. Comparative risk of cancer associated with SGLT inhibitors and DPP-4 inhibitors in patients with diabetes: a systematic review and meta-analysis. *Diabetol Metab Syndr* **2025**, *17*, 321, doi:10.1186/s13098-025-01898-z.
4. Hung, C.M.; Zeng, B.Y.; Hsu, C.W.; Chen, P.H.; Sun, C.K.; Carvalho, A.F.; Stubbs, B.; Chen, Y.W.; Chen, T.Y.; Lei, W.T.; et al. The different colorectal tumor risk related to GLP-1 receptor agonists and SGLT2 inhibitors use: a network meta-analysis of 68 randomized controlled trials. *Int J Surg* **2025**, doi:10.1097/JS9.0000000000003450.
5. Tseng, P.T.; Zeng, B.Y.; Hsu, C.W.; Sun, C.K.; Suen, M.W.; Carvalho, A.F.; Stubbs, B.; Chen, Y.W.; Chen, T.Y.; Lei, W.T.; et al. The gynecologic tumor risk related to GLP-1 receptor agonists and SGLT2 inhibitors use: a network meta-analysis of 91 randomized controlled trials. *J Hematol Oncol* **2025**, *18*, 109, doi:10.1186/s13045-025-01750-x.
6. Wang, L.; Xu, R.; Kaelber, D.C.; Berger, N.A. Glucagon-Like Peptide 1 Receptor Agonists and 13 Obesity-Associated Cancers in Patients With Type 2 Diabetes. *JAMA Netw Open* **2024**, *7*, e2421305, doi:10.1001/jamanetworkopen.2024.21305.
7. Moon, S.; Choi, J.W.; Park, J.H.; Kim, D.S.; Ahn, Y.; Kim, Y.; Kong, S.H.; Oh, C.M. Association of Appendicular Skeletal Muscle Mass Index and Insulin Resistance With Mortality in Multi-Nationwide Cohorts. *Journal of cachexia, sarcopenia and muscle* **2025**, *16*, e13811, doi:10.1002/jcsm.13811.
8. Packer, M.; Zile, M.R.; Kramer, C.M.; Baum, S.J.; Litwin, S.E.; Menon, V.; Ge, J.; Weerakkody, G.J.; Ou, Y.; Bunck, M.C.; et al. Tirzepatide for

Heart Failure with Preserved Ejection Fraction and Obesity. *N Engl J Med* **2025**, *392*, 427-437, doi:10.1056/NEJMoa2410027.
9. Aronne, L.J.; Sattar, N.; Horn, D.B.; Bays, H.E.; Wharton, S.; Lin, W.Y.; Ahmad, N.N.; Zhang, S.; Liao, R.; Bunck, M.C.; et al. Continued Treatment With Tirzepatide for Maintenance of Weight Reduction in Adults With Obesity: The SURMOUNT-4 Randomized Clinical Trial. *Jama* **2024**, *331*, 38-48, doi:10.1001/jama.2023.24945.
10. Bliddal, H.; Bays, H.; Czernichow, S.; Udden Hemmingsson, J.; Hjelmesaeth, J.; Hoffmann Morville, T.; Koroleva, A.; Skov Neergaard, J.; Velez Sanchez, P.; Wharton, S.; et al. Once-Weekly Semaglutide in Persons with Obesity and Knee Osteoarthritis. *N Engl J Med* **2024**, *391*, 1573-1583, doi:10.1056/NEJMoa2403664.
11. Docherty, K.F.; Buendia Lopez, R.; Folkvaljon, F.; de Boer, R.A.; Cowie, M.R.; Hammarstedt, A.; Kitzman, D.W.; Kosiborod, M.N.; Langkilde, A.M.; Reicher, B.; et al. Effect of Dapagliflozin on Accelerometer-Based Measures of Physical Activity in Patients With Heart Failure: An Analysis of the DETERMINE Trials. *Circ Heart Fail* **2024**, *17*, e012349, doi:10.1161/CIRCHEARTFAILURE.124.012349.
12. Ji, L.; Agesen, R.M.; Bain, S.C.; Fu, F.; Gabery, S.; Geng, J.; Li, Y.; Lu, Y.; Luo, B.; Pang, W.; et al. Efficacy and safety of oral semaglutide vs sitagliptin in a predominantly Chinese population with type 2 diabetes uncontrolled with metformin: PIONEER 12, a double-blind, Phase IIIa, randomised trial. *Diabetologia* **2024**, *67*, 1800-1816, doi:10.1007/s00125-024-06133-4.
13. Lee, B.W.; Cho, Y.M.; Kim, S.G.; Ko, S.H.; Lim, S.; Dahaoui, A.; Jeong, J.S.; Lim, H.J.; Yu, J.M. Efficacy and Safety of Once-Weekly Semaglutide Versus Once-Daily Sitagliptin as Metformin Add-on in a Korean Population with Type 2 Diabetes. *Diabetes Ther* **2024**, *15*, 547-563, doi:10.1007/s13300-023-01515-0.
14. McGowan, B.M.; Bruun, J.M.; Capehorn, M.; Pedersen, S.D.; Pietilainen, K.H.; Muniraju, H.A.K.; Quiroga, M.; Varbo, A.; Lau, D.C.W.; Group, S.S. Efficacy and safety of once-weekly semaglutide 2.4 mg versus placebo in people with obesity and prediabetes (STEP 10): a randomised, double-blind, placebo-controlled, multicentre phase 3 trial. *Lancet Diabetes Endocrinol* **2024**, *12*, 631-642, doi:10.1016/S2213-8587(24)00182-7.
15. McMurray, J.J.V.; Docherty, K.F.; de Boer, R.A.; Hammarstedt, A.; Kitzman, D.W.; Kosiborod, M.N.; Maria Langkilde, A.; Reicher, B.; Senni, M.; Shah, S.J.; et al. Effect of Dapagliflozin Versus Placebo on Symptoms and 6-Minute Walk Distance in Patients With Heart Failure: The DETERMINE Randomized Clinical Trials. *Circulation* **2024**, *149*, 825-838, doi:10.1161/CIRCULATIONAHA.123.065061.

16. Mu, Y.; Bao, X.; Eliaschewitz, F.G.; Hansen, M.R.; Kim, B.T.; Koroleva, A.; Ma, R.C.W.; Yang, T.; Zu, N.; Liu, M.; et al. Efficacy and safety of once weekly semaglutide 2.4 mg for weight management in a predominantly east Asian population with overweight or obesity (STEP 7): a double-blind, multicentre, randomised controlled trial. *Lancet Diabetes Endocrinol* **2024**, *12*, 184-195, doi:10.1016/S2213-8587(23)00388-1.
17. Tuttle, K.R.; Hauske, S.J.; Canziani, M.E.; Caramori, M.L.; Cherney, D.; Cronin, L.; Heerspink, H.J.L.; Hugo, C.; Nangaku, M.; Rotter, R.C.; et al. Efficacy and safety of aldosterone synthase inhibition with and without empagliflozin for chronic kidney disease: a randomised, controlled, phase 2 trial. *Lancet* **2024**, *403*, 379-390, doi:10.1016/S0140-6736(23)02408-X.
18. Wang, W.; Bain, S.C.; Bian, F.; Chen, R.; Gabery, S.; Huang, S.; Jensen, T.B.; Luo, B.; Yuan, G.; Ning, G.; et al. Efficacy and safety of oral semaglutide monotherapy vs placebo in a predominantly Chinese population with type 2 diabetes (PIONEER 11): a double-blind, Phase IIIa, randomised trial. *Diabetologia* **2024**, *67*, 1783-1799, doi:10.1007/s00125-024-06142-3.
19. Zhao, L.; Cheng, Z.; Lu, Y.; Liu, M.; Chen, H.; Zhang, M.; Wang, R.; Yuan, Y.; Li, X. Tirzepatide for Weight Reduction in Chinese Adults With Obesity: The SURMOUNT-CN Randomized Clinical Trial. *Jama* **2024**, *332*, 551-560, doi:10.1001/jama.2024.9217.
20. Aroda, V.R.; Frias, J.P.; Ji, L.; Niemoeller, E.; Nguyen-Pascal, M.L.; Denkel, K.; Espinasse, M.; Guo, H.; Baek, S.; Choi, J.; et al. Efficacy and safety of once-weekly efpeglenatide in people with suboptimally controlled type 2 diabetes: The AMPLITUDE-D, AMPLITUDE-L and AMPLITUDE-S randomized controlled trials. *Diabetes Obes Metab* **2023**, *25*, 2084-2095, doi:10.1111/dom.15079.
21. Aroda, V.R.; Aberle, J.; Bardtrum, L.; Christiansen, E.; Knop, F.K.; Gabery, S.; Pedersen, S.D.; Buse, J.B. Efficacy and safety of once-daily oral semaglutide 25 mg and 50 mg compared with 14 mg in adults with type 2 diabetes (PIONEER PLUS): a multicentre, randomised, phase 3b trial. *Lancet* **2023**, *402*, 693-704, doi:10.1016/S0140-6736(23)01127-3.
22. Cherney, D.Z.I.; Ferrannini, E.; Umpierrez, G.E.; Peters, A.L.; Rosenstock, J.; Powell, D.R.; Davies, M.J.; Banks, P.; Agarwal, R. Efficacy and safety of sotagliflozin in patients with type 2 diabetes and stage 3 chronic kidney disease. *Diabetes Obes Metab* **2023**, *25*, 1646-1657, doi:10.1111/dom.15019.
23. (SURPASS-CN-INS), E.L. A Study of Tirzepatide (LY3298176) in Chinese Participants With Type 2 Diabetes (SURPASS-CN-INS) (NCT05691712). Available online: https://clinicaltrials.gov/study/NCT05691712?cond=NCT05691712&rank=1 (accessed on 2025/9/1).

24. Feng, P.; Sheng, X.; Ji, Y.; Urva, S.; Wang, F.; Miller, S.; Qian, C.; An, Z.; Cui, Y. A Phase 1 Multiple Dose Study of Tirzepatide in Chinese Patients with Type 2 Diabetes. *Adv Ther* **2023**, *40*, 3434-3445, doi:10.1007/s12325-023-02536-8.
25. Frias, J.P.; Hsia, S.; Eyde, S.; Liu, R.; Ma, X.; Konig, M.; Kazda, C.; Mather, K.J.; Haupt, A.; Pratt, E.; et al. Efficacy and safety of oral orforglipron in patients with type 2 diabetes: a multicentre, randomised, dose-response, phase 2 study. *Lancet* **2023**, *402*, 472-483, doi:10.1016/S0140-6736(23)01302-8.
26. Gao, L.; Lee, B.W.; Chawla, M.; Kim, J.; Huo, L.; Du, L.; Huang, Y.; Ji, L. Tirzepatide versus insulin glargine as second-line or third-line therapy in type 2 diabetes in the Asia-Pacific region: the SURPASS-AP-Combo trial. *Nat Med* **2023**, *29*, 1500-1510, doi:10.1038/s41591-023-02344-1.
27. Jastreboff, A.M.; Kaplan, L.M.; Frias, J.P.; Wu, Q.; Du, Y.; Gurbuz, S.; Coskun, T.; Haupt, A.; Milicevic, Z.; Hartman, M.L.; et al. Triple-Hormone-Receptor Agonist Retatrutide for Obesity - A Phase 2 Trial. *N Engl J Med* **2023**, *389*, 514-526, doi:10.1056/NEJMoa2301972.
28. Ji, L.; Lu, Y.; Li, Q.; Fu, L.; Luo, Y.; Lei, T.; Li, L.; Ye, S.; Shi, B.; Li, X.; et al. Efficacy and safety of empagliflozin in combination with insulin in Chinese patients with type 2 diabetes and insufficient glycaemic control: A phase III, randomized, double-blind, placebo-controlled, parallel study. *Diabetes Obes Metab* **2023**, *25*, 1839-1848, doi:10.1111/dom.15041.
29. Kosiborod, M.N.; Abildstrom, S.Z.; Borlaug, B.A.; Butler, J.; Rasmussen, S.; Davies, M.; Hovingh, G.K.; Kitzman, D.W.; Lindegaard, M.L.; Moller, D.V.; et al. Semaglutide in Patients with Heart Failure with Preserved Ejection Fraction and Obesity. *N Engl J Med* **2023**, *389*, 1069-1084, doi:10.1056/NEJMoa2306963.
30. Loomba, R.; Abdelmalek, M.F.; Armstrong, M.J.; Jara, M.; Kjaer, M.S.; Krarup, N.; Lawitz, E.; Ratziu, V.; Sanyal, A.J.; Schattenberg, J.M.; et al. Semaglutide 2.4 mg once weekly in patients with non-alcoholic steatohepatitis-related cirrhosis: a randomised, placebo-controlled phase 2 trial. *Lancet Gastroenterol Hepatol* **2023**, *8*, 511-522, doi:10.1016/S2468-1253(23)00068-7.
31. Ramos, E.L.; Dayan, C.M.; Chatenoud, L.; Sumnik, Z.; Simmons, K.M.; Szypowska, A.; Gitelman, S.E.; Knecht, L.A.; Niemoeller, E.; Tian, W.; et al. Teplizumab and beta-Cell Function in Newly Diagnosed Type 1 Diabetes. *N Engl J Med* **2023**, *389*, 2151-2161, doi:10.1056/NEJMoa2308743.
32. Rosenstock, J.; Frias, J.; Jastreboff, A.M.; Du, Y.; Lou, J.; Gurbuz, S.; Thomas, M.K.; Hartman, M.L.; Haupt, A.; Milicevic, Z.; et al.

Retatrutide, a GIP, GLP-1 and glucagon receptor agonist, for people with type 2 diabetes: a randomised, double-blind, placebo and active-controlled, parallel-group, phase 2 trial conducted in the USA. *Lancet* **2023**, *402*, 529-544, doi:10.1016/S0140-6736(23)01053-X.

33. Rosenstock, J.; Frias, J.P.; Rodbard, H.W.; Tofe, S.; Sears, E.; Huh, R.; Fernandez Lando, L.; Patel, H. Tirzepatide vs Insulin Lispro Added to Basal Insulin in Type 2 Diabetes: The SURPASS-6 Randomized Clinical Trial. *Jama* **2023**, *330*, 1631-1640, doi:10.1001/jama.2023.20294.
34. Wadden, T.A.; Chao, A.M.; Machineni, S.; Kushner, R.; Ard, J.; Srivastava, G.; Halpern, B.; Zhang, S.; Chen, J.; Bunck, M.C.; et al. Tirzepatide after intensive lifestyle intervention in adults with overweight or obesity: the SURMOUNT-3 phase 3 trial. *Nat Med* **2023**, *29*, 2909-2918, doi:10.1038/s41591-023-02597-w.
35. Dahl, D.; Onishi, Y.; Norwood, P.; Huh, R.; Bray, R.; Patel, H.; Rodriguez, A. Effect of Subcutaneous Tirzepatide vs Placebo Added to Titrated Insulin Glargine on Glycemic Control in Patients With Type 2 Diabetes: The SURPASS-5 Randomized Clinical Trial. *Jama* **2022**, *327*, 534-545, doi:10.1001/jama.2022.0078.
36. Fox, C.K.; Clark, J.M.; Rudser, K.D.; Ryder, J.R.; Gross, A.C.; Nathan, B.M.; Sunni, M.; Dengel, D.R.; Billington, C.J.; Bensignor, M.O.; et al. Exenatide for weight-loss maintenance in adolescents with severe obesity: A randomized, placebo-controlled trial. *Obesity (Silver Spring)* **2022**, *30*, 1105-1115, doi:10.1002/oby.23395.
37. Frias, J.P.; Choi, J.; Rosenstock, J.; Popescu, L.; Niemoeller, E.; Muehlen-Bartmer, I.; Baek, S. Efficacy and Safety of Once-Weekly Efpeglenatide Monotherapy Versus Placebo in Type 2 Diabetes: The AMPLITUDE-M Randomized Controlled Trial. *Diabetes Care* **2022**, *45*, 1592-1600, doi:10.2337/dc21-2656.
38. Garvey, W.T.; Batterham, R.L.; Bhatta, M.; Buscemi, S.; Christensen, L.N.; Frias, J.P.; Jodar, E.; Kandler, K.; Rigas, G.; Wadden, T.A.; et al. Two-year effects of semaglutide in adults with overweight or obesity: the STEP 5 trial. *Nat Med* **2022**, *28*, 2083-2091, doi:10.1038/s41591-022-02026-4.
39. Heise, T.; Mari, A.; DeVries, J.H.; Urva, S.; Li, J.; Pratt, E.J.; Coskun, T.; Thomas, M.K.; Mather, K.J.; Haupt, A.; et al. Effects of subcutaneous tirzepatide versus placebo or semaglutide on pancreatic islet function and insulin sensitivity in adults with type 2 diabetes: a multicentre, randomised, double-blind, parallel-arm, phase 1 clinical trial. *Lancet Diabetes Endocrinol* **2022**, *10*, 418-429, doi:10.1016/S2213-8587(22)00085-7.

40. Inagaki, N.; Takeuchi, M.; Oura, T.; Imaoka, T.; Seino, Y. Efficacy and safety of tirzepatide monotherapy compared with dulaglutide in Japanese patients with type 2 diabetes (SURPASS J-mono): a double-blind, multicentre, randomised, phase 3 trial. *Lancet Diabetes Endocrinol* **2022**, *10*, 623-633, doi:10.1016/S2213-8587(22)00188-7.
41. Jastreboff, A.M.; Aronne, L.J.; Ahmad, N.N.; Wharton, S.; Connery, L.; Alves, B.; Kiyosue, A.; Zhang, S.; Liu, B.; Bunck, M.C.; et al. Tirzepatide Once Weekly for the Treatment of Obesity. *N Engl J Med* **2022**, *387*, 205-216, doi:10.1056/NEJMoa2206038.
42. Kadowaki, T.; Isendahl, J.; Khalid, U.; Lee, S.Y.; Nishida, T.; Ogawa, W.; Tobe, K.; Yamauchi, T.; Lim, S.; investigators, S. Semaglutide once a week in adults with overweight or obesity, with or without type 2 diabetes in an east Asian population (STEP 6): a randomised, double-blind, double-dummy, placebo-controlled, phase 3a trial. *Lancet Diabetes Endocrinol* **2022**, *10*, 193-206, doi:10.1016/S2213-8587(22)00008-0.
43. Kadowaki, T.; Chin, R.; Ozeki, A.; Imaoka, T.; Ogawa, Y. Safety and efficacy of tirzepatide as an add-on to single oral antihyperglycaemic medication in patients with type 2 diabetes in Japan (SURPASS J-combo): a multicentre, randomised, open-label, parallel-group, phase 3 trial. *Lancet Diabetes Endocrinol* **2022**, *10*, 634-644, doi:10.1016/S2213-8587(22)00187-5.
44. Nordisk, N. Research Study Investigating How Well Semaglutide Works in People From Thailand and South Korea Living With Obesity. Available online: https://clinicaltrials.gov/study/NCT04998136?cond=NCT04998136&rank=1 (accessed on 2025/5/20).
45. Rubino, D.M.; Greenway, F.L.; Khalid, U.; O'Neil, P.M.; Rosenstock, J.; Sorrig, R.; Wadden, T.A.; Wizert, A.; Garvey, W.T.; Investigators, S. Effect of Weekly Subcutaneous Semaglutide vs Daily Liraglutide on Body Weight in Adults With Overweight or Obesity Without Diabetes: The STEP 8 Randomized Clinical Trial. *Jama* **2022**, *327*, 138-150, doi:10.1001/jama.2021.23619.
46. Spertus, J.A.; Birmingham, M.C.; Nassif, M.; Damaraju, C.V.; Abbate, A.; Butler, J.; Lanfear, D.E.; Lingvay, I.; Kosiborod, M.N.; Januzzi, J.L. The SGLT2 inhibitor canagliflozin in heart failure: the CHIEF-HF remote, patient-centered randomized trial. *Nat Med* **2022**, *28*, 809-813, doi:10.1038/s41591-022-01703-8.
47. Voors, A.A.; Angermann, C.E.; Teerlink, J.R.; Collins, S.P.; Kosiborod, M.; Biegus, J.; Ferreira, J.P.; Nassif, M.E.; Psotka, M.A.; Tromp, J.; et al. The SGLT2 inhibitor empagliflozin in patients hospitalized for acute heart failure: a multinational randomized trial. *Nat Med* **2022**, *28*, 568-574, doi:10.1038/s41591-021-01659-1.

48. Anker, S.D.; Ponikowski, P.; Wanner, C.; Pfarr, E.; Hauske, S.; Peil, B.; Salsali, A.; Ritter, I.; Koitka-Weber, A.; Brueckmann, M.; et al. Kidney Function After Initiation and Discontinuation of Empagliflozin in Patients With Heart Failure With and Without Type 2 Diabetes: Insights From the EMPERIAL Trials. *Circulation* **2021**, *144*, 1265-1267, doi:10.1161/CIRCULATIONAHA.121.054669.
49. Del Prato, S.; Kahn, S.E.; Pavo, I.; Weerakkody, G.J.; Yang, Z.; Doupis, J.; Aizenberg, D.; Wynne, A.G.; Riesmeyer, J.S.; Heine, R.J.; et al. Tirzepatide versus insulin glargine in type 2 diabetes and increased cardiovascular risk (SURPASS-4): a randomised, open-label, parallel-group, multicentre, phase 3 trial. *Lancet* **2021**, *398*, 1811-1824, doi:10.1016/S0140-6736(21)02188-7.
50. Frias, J.P.; Davies, M.J.; Rosenstock, J.; Perez Manghi, F.C.; Fernandez Lando, L.; Bergman, B.K.; Liu, B.; Cui, X.; Brown, K.; Investigators, S.-. Tirzepatide versus Semaglutide Once Weekly in Patients with Type 2 Diabetes. *N Engl J Med* **2021**, *385*, 503-515, doi:10.1056/NEJMoa2107519.
51. Frias, J.P.; Auerbach, P.; Bajaj, H.S.; Fukushima, Y.; Lingvay, I.; Macura, S.; Sondergaard, A.L.; Tankova, T.I.; Tentolouris, N.; Buse, J.B. Efficacy and safety of once-weekly semaglutide 2.0 mg versus 1.0 mg in patients with type 2 diabetes (SUSTAIN FORTE): a double-blind, randomised, phase 3B trial. *Lancet Diabetes Endocrinol* **2021**, *9*, 563-574, doi:10.1016/S2213-8587(21)00174-1.
52. Gerstein, H.C.; Sattar, N.; Rosenstock, J.; Ramasundarahettige, C.; Pratley, R.; Lopes, R.D.; Lam, C.S.P.; Khurmi, N.S.; Heenan, L.; Del Prato, S.; et al. Cardiovascular and Renal Outcomes with Efpeglenatide in Type 2 Diabetes. *N Engl J Med* **2021**, *385*, 896-907, doi:10.1056/NEJMoa2108269.
53. Kosiborod, M.N.; Esterline, R.; Furtado, R.H.M.; Oscarsson, J.; Gasparyan, S.B.; Koch, G.G.; Martinez, F.; Mukhtar, O.; Verma, S.; Chopra, V.; et al. Dapagliflozin in patients with cardiometabolic risk factors hospitalised with COVID-19 (DARE-19): a randomised, double-blind, placebo-controlled, phase 3 trial. *Lancet Diabetes Endocrinol* **2021**, *9*, 586-594, doi:10.1016/S2213-8587(21)00180-7.
54. Nassif, M.E.; Spertus, J.A.; Tang, F.; Windsor, S.L.; Jones, P.; Thomas, M.; Khariton, Y.; Brush, J.; Gordon, R.A.; Jermyn, R.; et al. Association Between Change in Ambulatory Hemodynamic Pressures and Symptoms of Heart Failure. *Circ Heart Fail* **2021**, *14*, e008446, doi:10.1161/CIRCHEARTFAILURE.121.008446.
55. Nassif, M.E.; Windsor, S.L.; Borlaug, B.A.; Kitzman, D.W.; Shah, S.J.; Tang, F.; Khariton, Y.; Malik, A.O.; Khumri, T.; Umpierrez, G.; et al. The SGLT2 inhibitor dapagliflozin in heart failure with preserved ejection fraction: a multicenter randomized trial. *Nat Med* **2021**, *27*, 1954-

1960, doi:10.1038/s41591-021-01536-x.
56. Rodgers, M.; Migdal, A.L.; Rodriguez, T.G.; Chen, Z.Z.; Nath, A.K.; Gerszten, R.E.; Kasid, N.; Toschi, E.; Tripaldi, J.; Heineman, B.; et al. Weight Loss Outcomes Among Early High Responders to Exenatide Treatment: A Randomized, Placebo Controlled Study in Overweight and Obese Women. *Front Endocrinol (Lausanne)* **2021**, *12*, 742873, doi:10.3389/fendo.2021.742873.
57. Rosenstock, J.; Wysham, C.; Frias, J.P.; Kaneko, S.; Lee, C.J.; Fernandez Lando, L.; Mao, H.; Cui, X.; Karanikas, C.A.; Thieu, V.T. Efficacy and safety of a novel dual GIP and GLP-1 receptor agonist tirzepatide in patients with type 2 diabetes (SURPASS-1): a double-blind, randomised, phase 3 trial. *Lancet* **2021**, *398*, 143-155, doi:10.1016/S0140-6736(21)01324-6.
58. Rubino, D.; Abrahamsson, N.; Davies, M.; Hesse, D.; Greenway, F.L.; Jensen, C.; Lingvay, I.; Mosenzon, O.; Rosenstock, J.; Rubio, M.A.; et al. Effect of Continued Weekly Subcutaneous Semaglutide vs Placebo on Weight Loss Maintenance in Adults With Overweight or Obesity: The STEP 4 Randomized Clinical Trial. *Jama* **2021**, *325*, 1414-1425, doi:10.1001/jama.2021.3224.
59. Santos-Gallego, C.G.; Vargas-Delgado, A.P.; Requena-Ibanez, J.A.; Garcia-Ropero, A.; Mancini, D.; Pinney, S.; Macaluso, F.; Sartori, S.; Roque, M.; Sabatel-Perez, F.; et al. Randomized Trial of Empagliflozin in Nondiabetic Patients With Heart Failure and Reduced Ejection Fraction. *J Am Coll Cardiol* **2021**, *77*, 243-255, doi:10.1016/j.jacc.2020.11.008.
60. Stack, A.G.; Han, D.; Goldwater, R.; Johansson, S.; Dronamraju, N.; Oscarsson, J.; Johnsson, E.; Parkinson, J.; Erlandsson, F. Dapagliflozin Added to Verinurad Plus Febuxostat Further Reduces Serum Uric Acid in Hyperuricemia: The QUARTZ Study. *J Clin Endocrinol Metab* **2021**, *106*, e2347-e2356, doi:10.1210/clinem/dgaa748.
61. Wadden, T.A.; Bailey, T.S.; Billings, L.K.; Davies, M.; Frias, J.P.; Koroleva, A.; Lingvay, I.; O'Neil, P.M.; Rubino, D.M.; Skovgaard, D.; et al. Effect of Subcutaneous Semaglutide vs Placebo as an Adjunct to Intensive Behavioral Therapy on Body Weight in Adults With Overweight or Obesity: The STEP 3 Randomized Clinical Trial. *Jama* **2021**, *325*, 1403-1413, doi:10.1001/jama.2021.1831.
62. Wason, S. Efficacy and Bone Safety of Sotagliflozin 400 and 200 mg Versus Placebo in Participants With Type 2 Diabetes Mellitus Who Have Inadequate Glycemic Control (SOTA-BONE). Available online: https://clinicaltrials.gov/study/NCT03386344?cond=NCT03386344&rank=1 (accessed on 2024/10/28).
63. Wason, S. Efficacy and Safety of Sotagliflozin Versus Placebo in Participants With Type 2 Diabetes Mellitus Who Have Inadequate

Glycemic Control While Taking Insulin Alone or With Other Oral Antidiabetic Agents (SOTA-INS). Available online: https://clinicaltrials.gov/study/NCT03285594?cond=NCT03285594&rank=1 (accessed on 2024/10/28).
64. Yang, W.; Xu, X.; Lei, T.; Ma, J.; Li, L.; Shen, J.; Ye, B.; Zhu, S.; Meinicke, T. Efficacy and safety of linagliptin as add-on therapy to insulin in Chinese patients with type 2 diabetes mellitus: A randomized, double-blind, placebo-controlled trial. *Diabetes Obes Metab* **2021**, *23*, 642-647, doi:10.1111/dom.14231.
65. Capehorn, M.S.; Catarig, A.M.; Furberg, J.K.; Janez, A.; Price, H.C.; Tadayon, S.; Verges, B.; Marre, M. Efficacy and safety of once-weekly semaglutide 1.0mg vs once-daily liraglutide 1.2mg as add-on to 1-3 oral antidiabetic drugs in subjects with type 2 diabetes (SUSTAIN 10). *Diabetes Metab* **2020**, *46*, 100-109, doi:10.1016/j.diabet.2019.101117.
66. Aroda, V.R.; Rosenstock, J.; Terauchi, Y.; Altuntas, Y.; Lalic, N.M.; Morales Villegas, E.C.; Jeppesen, O.K.; Christiansen, E.; Hertz, C.L.; Haluzik, M.; et al. PIONEER 1: Randomized Clinical Trial of the Efficacy and Safety of Oral Semaglutide Monotherapy in Comparison With Placebo in Patients With Type 2 Diabetes. *Diabetes Care* **2019**, *42*, 1724-1732, doi:10.2337/dc19-0749.
67. Gallo, S.; Charbonnel, B.; Goldman, A.; Shi, H.; Huyck, S.; Darekar, A.; Lauring, B.; Terra, S.G. Long-term efficacy and safety of ertugliflozin in patients with type 2 diabetes mellitus inadequately controlled with metformin monotherapy: 104-week VERTIS MET trial. *Diabetes Obes Metab* **2019**, *21*, 1027-1036, doi:10.1111/dom.13631.
68. Herold, K.C.; Bundy, B.N.; Long, S.A.; Bluestone, J.A.; DiMeglio, L.A.; Dufort, M.J.; Gitelman, S.E.; Gottlieb, P.A.; Krischer, J.P.; Linsley, P.S.; et al. An Anti-CD3 Antibody, Teplizumab, in Relatives at Risk for Type 1 Diabetes. *N Engl J Med* **2019**, *381*, 603-613, doi:10.1056/NEJMoa1902226.
69. Lingvay, I.; Catarig, A.M.; Frias, J.P.; Kumar, H.; Lausvig, N.L.; le Roux, C.W.; Thielke, D.; Viljoen, A.; McCrimmon, R.J. Efficacy and safety of once-weekly semaglutide versus daily canagliflozin as add-on to metformin in patients with type 2 diabetes (SUSTAIN 8): a double-blind, phase 3b, randomised controlled trial. *Lancet Diabetes Endocrinol* **2019**, *7*, 834-844, doi:10.1016/S2213-8587(19)30311-0.
70. Mosenzon, O.; Blicher, T.M.; Rosenlund, S.; Eriksson, J.W.; Heller, S.; Hels, O.H.; Pratley, R.; Sathyapalan, T.; Desouza, C.; Investigators, P. Efficacy and safety of oral semaglutide in patients with type 2 diabetes and moderate renal impairment (PIONEER 5): a placebo-controlled, randomised, phase 3a trial. *Lancet Diabetes Endocrinol* **2019**, *7*, 515-527, doi:10.1016/S2213-8587(19)30192-5.

71. Mullins, R.J.; Mustapic, M.; Chia, C.W.; Carlson, O.; Gulyani, S.; Tran, J.; Li, Y.; Mattson, M.P.; Resnick, S.; Egan, J.M.; et al. A Pilot Study of Exenatide Actions in Alzheimer's Disease. *Curr Alzheimer Res* **2019**, *16*, 741-752, doi:10.2174/1567205016666190913155950.
72. Nassif, M.E.; Windsor, S.L.; Tang, F.; Khariton, Y.; Husain, M.; Inzucchi, S.E.; McGuire, D.K.; Pitt, B.; Scirica, B.M.; Austin, B.; et al. Dapagliflozin Effects on Biomarkers, Symptoms, and Functional Status in Patients With Heart Failure With Reduced Ejection Fraction: The DEFINE-HF Trial. *Circulation* **2019**, *140*, 1463-1476, doi:10.1161/CIRCULATIONAHA.119.042929.
73. Pollock, C.; Stefansson, B.; Reyner, D.; Rossing, P.; Sjostrom, C.D.; Wheeler, D.C.; Langkilde, A.M.; Heerspink, H.J.L. Albuminuria-lowering effect of dapagliflozin alone and in combination with saxagliptin and effect of dapagliflozin and saxagliptin on glycaemic control in patients with type 2 diabetes and chronic kidney disease (DELIGHT): a randomised, double-blind, placebo-controlled trial. *Lancet Diabetes Endocrinol* **2019**, *7*, 429-441, doi:10.1016/S2213-8587(19)30086-5.
74. Pratley, R.; Amod, A.; Hoff, S.T.; Kadowaki, T.; Lingvay, I.; Nauck, M.; Pedersen, K.B.; Saugstrup, T.; Meier, J.J.; investigators, P. Oral semaglutide versus subcutaneous liraglutide and placebo in type 2 diabetes (PIONEER 4): a randomised, double-blind, phase 3a trial. *Lancet* **2019**, *394*, 39-50, doi:10.1016/S0140-6736(19)31271-1.
75. Rodbard, H.W.; Rosenstock, J.; Canani, L.H.; Deerochanawong, C.; Gumprecht, J.; Lindberg, S.O.; Lingvay, I.; Sondergaard, A.L.; Treppendahl, M.B.; Montanya, E.; et al. Oral Semaglutide Versus Empagliflozin in Patients With Type 2 Diabetes Uncontrolled on Metformin: The PIONEER 2 Trial. *Diabetes Care* **2019**, *42*, 2272-2281, doi:10.2337/dc19-0883.
76. Rosenstock, J.; Perl, S.; Johnsson, E.; Garcia-Sanchez, R.; Jacob, S. Triple therapy with low-dose dapagliflozin plus saxagliptin versus dual therapy with each monocomponent, all added to metformin, in uncontrolled type 2 diabetes. *Diabetes Obes Metab* **2019**, *21*, 2152-2162, doi:10.1111/dom.13795.
77. Wang, J.; Li, H.Q.; Xu, X.H.; Kong, X.C.; Sun, R.; Jing, T.; Ye, L.; Su, X.F.; Ma, J.H. The Effects of Once-Weekly Dulaglutide and Insulin Glargine on Glucose Fluctuation in Poorly Oral-Antidiabetic Controlled Patients with Type 2 Diabetes Mellitus. *Biomed Res Int* **2019**, *2019*, 2682657, doi:10.1155/2019/2682657.
78. Ahmann, A.J.; Capehorn, M.; Charpentier, G.; Dotta, F.; Henkel, E.; Lingvay, I.; Holst, A.G.; Annett, M.P.; Aroda, V.R. Efficacy and Safety of Once-Weekly Semaglutide Versus Exenatide ER in Subjects With Type 2 Diabetes (SUSTAIN 3): A 56-Week, Open-Label, Randomized

Clinical Trial. *Diabetes Care* **2018**, *41*, 258-266, doi:10.2337/dc17-0417.
79. Aronson, R.; Frias, J.; Goldman, A.; Darekar, A.; Lauring, B.; Terra, S.G. Long-term efficacy and safety of ertugliflozin monotherapy in patients with inadequately controlled T2DM despite diet and exercise: VERTIS MONO extension study. *Diabetes Obes Metab* **2018**, *20*, 1453-1460, doi:10.1111/dom.13251.
80. Buse, J.B.; Garg, S.K.; Rosenstock, J.; Bailey, T.S.; Banks, P.; Bode, B.W.; Danne, T.; Kushner, J.A.; Lane, W.S.; Lapuerta, P.; et al. Sotagliflozin in Combination With Optimized Insulin Therapy in Adults With Type 1 Diabetes: The North American inTandem1 Study. *Diabetes Care* **2018**, *41*, 1970-1980, doi:10.2337/dc18-0343.
81. Coskun, T.; Sloop, K.W.; Loghin, C.; Alsina-Fernandez, J.; Urva, S.; Bokvist, K.B.; Cui, X.; Briere, D.A.; Cabrera, O.; Roell, W.C.; et al. LY3298176, a novel dual GIP and GLP-1 receptor agonist for the treatment of type 2 diabetes mellitus: From discovery to clinical proof of concept. *Mol Metab* **2018**, *18*, 3-14, doi:10.1016/j.molmet.2018.09.009.
82. Danne, T.; Cariou, B.; Banks, P.; Brandle, M.; Brath, H.; Franek, E.; Kushner, J.A.; Lapuerta, P.; McGuire, D.K.; Peters, A.L.; et al. HbA(1c) and Hypoglycemia Reductions at 24 and 52 Weeks With Sotagliflozin in Combination With Insulin in Adults With Type 1 Diabetes: The European inTandem2 Study. *Diabetes Care* **2018**, *41*, 1981-1990, doi:10.2337/dc18-0342.
83. Dou, J.; Ma, J.; Liu, J.; Wang, C.; Johnsson, E.; Yao, H.; Zhao, J.; Pan, C. Efficacy and safety of saxagliptin in combination with metformin as initial therapy in Chinese patients with type 2 diabetes: Results from the START study, a multicentre, randomized, double-blind, active-controlled, phase 3 trial. *Diabetes Obes Metab* **2018**, *20*, 590-598, doi:10.1111/dom.13117.
84. Frias, J.P.; Nauck, M.A.; Van, J.; Kutner, M.E.; Cui, X.; Benson, C.; Urva, S.; Gimeno, R.E.; Milicevic, Z.; Robins, D.; et al. Efficacy and safety of LY3298176, a novel dual GIP and GLP-1 receptor agonist, in patients with type 2 diabetes: a randomised, placebo-controlled and active comparator-controlled phase 2 trial. *Lancet* **2018**, *392*, 2180-2193, doi:10.1016/S0140-6736(18)32260-8.
85. Grunberger, G.; Camp, S.; Johnson, J.; Huyck, S.; Terra, S.G.; Mancuso, J.P.; Jiang, Z.W.; Golm, G.; Engel, S.S.; Lauring, B. Ertugliflozin in Patients with Stage 3 Chronic Kidney Disease and Type 2 Diabetes Mellitus: The VERTIS RENAL Randomized Study. *Diabetes Ther* **2018**, *9*, 49-66, doi:10.1007/s13300-017-0337-5.
86. Iwamoto, N.; Matsui, A.; Kazama, H.; Oura, T. Subgroup Analysis Stratified by Baseline Pancreatic beta-cell Function in a Japanese Study

of Dulaglutide in Patients with Type 2 Diabetes. *Diabetes Ther* **2018**, *9*, 383-394, doi:10.1007/s13300-017-0346-4.
87. Ludvik, B.; Frias, J.P.; Tinahones, F.J.; Wainstein, J.; Jiang, H.; Robertson, K.E.; Garcia-Perez, L.E.; Woodward, D.B.; Milicevic, Z. Dulaglutide as add-on therapy to SGLT2 inhibitors in patients with inadequately controlled type 2 diabetes (AWARD-10): a 24-week, randomised, double-blind, placebo-controlled trial. *Lancet Diabetes Endocrinol* **2018**, *6*, 370-381, doi:10.1016/S2213-8587(18)30023-8.
88. Muller-Wieland, D.; Kellerer, M.; Cypryk, K.; Skripova, D.; Rohwedder, K.; Johnsson, E.; Garcia-Sanchez, R.; Kurlyandskaya, R.; Sjostrom, C.D.; Jacob, S.; et al. Efficacy and safety of dapagliflozin or dapagliflozin plus saxagliptin versus glimepiride as add-on to metformin in patients with type 2 diabetes. *Diabetes Obes Metab* **2018**, *20*, 2598-2607, doi:10.1111/dom.13437.
89. O'Neil, P.M.; Birkenfeld, A.L.; McGowan, B.; Mosenzon, O.; Pedersen, S.D.; Wharton, S.; Carson, C.G.; Jepsen, C.H.; Kabisch, M.; Wilding, J.P.H. Efficacy and safety of semaglutide compared with liraglutide and placebo for weight loss in patients with obesity: a randomised, double-blind, placebo and active controlled, dose-ranging, phase 2 trial. *Lancet* **2018**, *392*, 637-649, doi:10.1016/S0140-6736(18)31773-2.
90. Pratley, R.E.; Aroda, V.R.; Lingvay, I.; Ludemann, J.; Andreassen, C.; Navarria, A.; Viljoen, A.; investigators, S. Semaglutide versus dulaglutide once weekly in patients with type 2 diabetes (SUSTAIN 7): a randomised, open-label, phase 3b trial. *Lancet Diabetes Endocrinol* **2018**, *6*, 275-286, doi:10.1016/S2213-8587(18)30024-X.
91. Pratley, R.E.; Eldor, R.; Raji, A.; Golm, G.; Huyck, S.B.; Qiu, Y.; Sunga, S.; Johnson, J.; Terra, S.G.; Mancuso, J.P.; et al. Ertugliflozin plus sitagliptin versus either individual agent over 52 weeks in patients with type 2 diabetes mellitus inadequately controlled with metformin: The VERTIS FACTORIAL randomized trial. *Diabetes Obes Metab* **2018**, *20*, 1111-1120, doi:10.1111/dom.13194.
92. Rodbard, H.W.; Lingvay, I.; Reed, J.; de la Rosa, R.; Rose, L.; Sugimoto, D.; Araki, E.; Chu, P.L.; Wijayasinghe, N.; Norwood, P. Semaglutide Added to Basal Insulin in Type 2 Diabetes (SUSTAIN 5): A Randomized, Controlled Trial. *J Clin Endocrinol Metab* **2018**, *103*, 2291-2301, doi:10.1210/jc.2018-00070.
93. Scott, R.; Morgan, J.; Zimmer, Z.; Lam, R.L.H.; O'Neill, E.A.; Kaufman, K.D.; Engel, S.S.; Raji, A. A randomized clinical trial of the efficacy and safety of sitagliptin compared with dapagliflozin in patients with type 2 diabetes mellitus and mild renal insufficiency: The CompoSIT-R study. *Diabetes Obes Metab* **2018**, *20*, 2876-2884, doi:10.1111/dom.13473.

94. Seino, Y.; Terauchi, Y.; Osonoi, T.; Yabe, D.; Abe, N.; Nishida, T.; Zacho, J.; Kaneko, S. Safety and efficacy of semaglutide once weekly vs sitagliptin once daily, both as monotherapy in Japanese people with type 2 diabetes. *Diabetes Obes Metab* **2018**, *20*, 378-388, doi:10.1111/dom.13082.
95. Zhu, D.; Gan, S.; Liu, Y.; Ma, J.; Dong, X.; Song, W.; Zeng, J.; Wang, G.; Zhao, W.; Zhang, Q.; et al. Dorzagliatin monotherapy in Chinese patients with type 2 diabetes: a dose-ranging, randomised, double-blind, placebo-controlled, phase 2 study. *Lancet Diabetes Endocrinol* **2018**, *6*, 627-636, doi:10.1016/S2213-8587(18)30105-0.
96. Ahren, B.; Masmiquel, L.; Kumar, H.; Sargin, M.; Karsbol, J.D.; Jacobsen, S.H.; Chow, F. Efficacy and safety of once-weekly semaglutide versus once-daily sitagliptin as an add-on to metformin, thiazolidinediones, or both, in patients with type 2 diabetes (SUSTAIN 2): a 56-week, double-blind, phase 3a, randomised trial. *Lancet Diabetes Endocrinol* **2017**, *5*, 341-354, doi:10.1016/S2213-8587(17)30092-X.
97. Aroda, V.R.; Bain, S.C.; Cariou, B.; Piletic, M.; Rose, L.; Axelsen, M.; Rowe, E.; DeVries, J.H. Efficacy and safety of once-weekly semaglutide versus once-daily insulin glargine as add-on to metformin (with or without sulfonylureas) in insulin-naive patients with type 2 diabetes (SUSTAIN 4): a randomised, open-label, parallel-group, multicentre, multinational, phase 3a trial. *Lancet Diabetes Endocrinol* **2017**, *5*, 355-366, doi:10.1016/S2213-8587(17)30085-2.
98. Ba, J.; Han, P.; Yuan, G.; Mo, Z.; Pan, C.; Wu, F.; Xu, L.; Hanson, M.E.; Engel, S.S.; Shankar, R.R. Randomized trial assessing the safety and efficacy of sitagliptin in Chinese patients with type 2 diabetes mellitus inadequately controlled on sulfonylurea alone or combined with metformin. *J Diabetes* **2017**, *9*, 667-676, doi:10.1111/1753-0407.12456.
99. Du, J.; Liang, L.; Fang, H.; Xu, F.; Li, W.; Shen, L.; Wang, X.; Xu, C.; Bian, F.; Mu, Y. Efficacy and safety of saxagliptin compared with acarbose in Chinese patients with type 2 diabetes mellitus uncontrolled on metformin monotherapy: Results of a Phase IV open-label randomized controlled study (the SMART study). *Diabetes Obes Metab* **2017**, *19*, 1513-1520, doi:10.1111/dom.12942.
100. Gadde, K.M.; Vetter, M.L.; Iqbal, N.; Hardy, E.; Ohman, P.; investigators, D.-N.-s. Efficacy and safety of autoinjected exenatide once-weekly suspension versus sitagliptin or placebo with metformin in patients with type 2 diabetes: The DURATION-NEO-2 randomized clinical study. *Diabetes Obes Metab* **2017**, *19*, 979-988, doi:10.1111/dom.12908.
101. Gantz, I.; Okamoto, T.; Ito, Y.; Okuyama, K.; O'Neill, E.A.; Kaufman, K.D.; Engel, S.S.; Lai, E.; the Omarigliptin Study, G. A randomized,

placebo- and sitagliptin-controlled trial of the safety and efficacy of omarigliptin, a once-weekly dipeptidyl peptidase-4 inhibitor, in Japanese patients with type 2 diabetes. *Diabetes Obes Metab* **2017**, *19*, 1602-1609, doi:10.1111/dom.12988.
102. Handelsman, Y.; Lauring, B.; Gantz, I.; Iredale, C.; O'Neill, E.A.; Wei, Z.; Suryawanshi, S.; Kaufman, K.D.; Engel, S.S.; Lai, E. A randomized, double-blind, non-inferiority trial evaluating the efficacy and safety of omarigliptin, a once-weekly DPP-4 inhibitor, or glimepiride in patients with type 2 diabetes inadequately controlled on metformin monotherapy. *Curr Med Res Opin* **2017**, *33*, 1861-1868, doi:10.1080/03007995.2017.1335638.
103. Trials, G.C. Safety and Efficacy of Albiglutide in Type 2 Diabetes: HARMONY 1 (NCT00849056). Available online: https://clinicaltrials.gov/study/NCT00849056?cond=NCT00849056&rank=1 (accessed on 2026/01/07).
104. Trials, G.C. Efficacy and Safety of Albiglutide in Treatment of Type 2 Diabetes: HARMONY 3 (NCT00838903). Available online: https://clinicaltrials.gov/study/NCT00838903?cond=NCT00838903&rank=1 (accessed on 2026/01/07).
105. Trials, G.C. A Study to Determine the Safety and Efficacy of Albiglutide in Subjects With Type 2 Diabetes: HARMONY 5 (NCT00839527). Available online: https://clinicaltrials.gov/study/NCT00839527?cond=NCT00839527&rank=1 (accessed on 2026/01/07).
106. Januzzi, J.L., Jr.; Butler, J.; Jarolim, P.; Sattar, N.; Vijapurkar, U.; Desai, M.; Davies, M.J. Effects of Canagliflozin on Cardiovascular Biomarkers in Older Adults With Type 2 Diabetes. *J Am Coll Cardiol* **2017**, *70*, 704-712, doi:10.1016/j.jacc.2017.06.016.
107. Lee, S.H.; Gantz, I.; Round, E.; Latham, M.; O'Neill, E.A.; Ceesay, P.; Suryawanshi, S.; Kaufman, K.D.; Engel, S.S.; Lai, E. A randomized, placebo-controlled clinical trial evaluating the safety and efficacy of the once-weekly DPP-4 inhibitor omarigliptin in patients with type 2 diabetes mellitus inadequately controlled by glimepiride and metformin. *BMC Endocr Disord* **2017**, *17*, 70, doi:10.1186/s12902-017-0219-x.
108. Meneilly, G.S.; Roy-Duval, C.; Alawi, H.; Dailey, G.; Bellido, D.; Trescoli, C.; Manrique Hurtado, H.; Guo, H.; Pilorget, V.; Perfetti, R.; et al. Lixisenatide Therapy in Older Patients With Type 2 Diabetes Inadequately Controlled on Their Current Antidiabetic Treatment: The GetGoal-O Randomized Trial. *Diabetes Care* **2017**, *40*, 485-493, doi:10.2337/dc16-2143.
109. Mu, Y.; Pan, C.; Fan, B.; Hehnke, U.; Zhang, X.; Zhang, X.; Wang, X.; Liu, J.; Zhang, Y.; Du, J.; et al. Efficacy and safety of linagliptin/metformin single-pill combination as initial therapy in drug-naive Asian patients with type 2 diabetes. *Diabetes Res Clin Pract*

2017, *124*, 48-56, doi:10.1016/j.diabres.2016.11.026.

110. Pan, C.; Han, P.; Ji, Q.; Li, C.; Lu, J.; Yang, J.; Li, W.; Zeng, J.; Hsieh, A.T.; Chan, J. Efficacy and safety of alogliptin in patients with type 2 diabetes mellitus: A multicentre randomized double-blind placebo-controlled Phase 3 study in mainland China, Taiwan, and Hong Kong. *J Diabetes* **2017**, *9*, 386-395, doi:10.1111/1753-0407.12425.
111. Shankar, R.R.; Inzucchi, S.E.; Scarabello, V.; Gantz, I.; Kaufman, K.D.; Lai, E.; Ceesay, P.; Suryawanshi, S.; Engel, S.S. A randomized clinical trial evaluating the efficacy and safety of the once-weekly dipeptidyl peptidase-4 inhibitor omarigliptin in patients with type 2 diabetes inadequately controlled on metformin monotherapy. *Curr Med Res Opin* **2017**, *33*, 1853-1860, doi:10.1080/03007995.2017.1335637.
112. Sorli, C.; Harashima, S.I.; Tsoukas, G.M.; Unger, J.; Karsbol, J.D.; Hansen, T.; Bain, S.C. Efficacy and safety of once-weekly semaglutide monotherapy versus placebo in patients with type 2 diabetes (SUSTAIN 1): a double-blind, randomised, placebo-controlled, parallel-group, multinational, multicentre phase 3a trial. *Lancet Diabetes Endocrinol* **2017**, *5*, 251-260, doi:10.1016/S2213-8587(17)30013-X.
113. Terauchi, Y.; Yamada, Y.; Ishida, H.; Ohsugi, M.; Kitaoka, M.; Satoh, J.; Yabe, D.; Shihara, N.; Seino, Y. Efficacy and safety of sitagliptin as compared with glimepiride in Japanese patients with type 2 diabetes mellitus aged >/= 60 years (START-J trial). *Diabetes Obes Metab* **2017**, *19*, 1188-1192, doi:10.1111/dom.12933.
114. Tinahones, F.J.; Gallwitz, B.; Nordaby, M.; Gotz, S.; Maldonado-Lutomirsky, M.; Woerle, H.J.; Broedl, U.C. Linagliptin as add-on to empagliflozin and metformin in patients with type 2 diabetes: Two 24-week randomized, double-blind, double-dummy, parallel-group trials. *Diabetes Obes Metab* **2017**, *19*, 266-274, doi:10.1111/dom.12814.
115. Wang, W.; Ning, G.; Ma, J.; Liu, X.; Zheng, S.; Wu, F.; Xu, L.; O'Neill, E.A.; Fujita, K.P.; Engel, S.S.; et al. A randomized clinical trial of the safety and efficacy of sitagliptin in patients with type 2 diabetes mellitus inadequately controlled by acarbose alone. *Curr Med Res Opin* **2017**, *33*, 693-699, doi:10.1080/03007995.2016.1277200.
116. Yu, M.; Brunt, K.V.; Milicevic, Z.; Varnado, O.; Boye, K.S. Patient-reported Outcomes in Patients with Type 2 Diabetes Treated with Dulaglutide Added to Titrated Insulin Glargine (AWARD-9). *Clin Ther* **2017**, *39*, 2284-2295, doi:10.1016/j.clinthera.2017.10.002.
117. Davies, M.J.; Bain, S.C.; Atkin, S.L.; Rossing, P.; Scott, D.; Shamkhalova, M.S.; Bosch-Traberg, H.; Syren, A.; Umpierrez, G.E. Efficacy and Safety of Liraglutide Versus Placebo as Add-on to Glucose-Lowering Therapy in Patients With Type 2 Diabetes and Moderate Renal

Impairment (LIRA-RENAL): A Randomized Clinical Trial. *Diabetes Care* **2016**, *39*, 222-230, doi:10.2337/dc14-2883.
118. Dungan, K.M.; Weitgasser, R.; Perez Manghi, F.; Pintilei, E.; Fahrbach, J.L.; Jiang, H.H.; Shell, J.; Robertson, K.E. A 24-week study to evaluate the efficacy and safety of once-weekly dulaglutide added on to glimepiride in type 2 diabetes (AWARD-8). *Diabetes Obes Metab* **2016**, *18*, 475-482, doi:10.1111/dom.12634.
119. Investigators, F.-S.T. Glucose Variability in a 26-Week Randomized Comparison of Mealtime Treatment With Rapid-Acting Insulin Versus GLP-1 Agonist in Participants With Type 2 Diabetes at High Cardiovascular Risk. *Diabetes Care* **2016**, *39*, 973-981, doi:10.2337/dc15-2782.
120. Hadjadj, S.; Rosenstock, J.; Meinicke, T.; Woerle, H.J.; Broedl, U.C. Initial Combination of Empagliflozin and Metformin in Patients With Type 2 Diabetes. *Diabetes Care* **2016**, *39*, 1718-1728, doi:10.2337/dc16-0522.
121. Leiter, L.A.; Cefalu, W.T.; de Bruin, T.W.; Xu, J.; Parikh, S.; Johnsson, E.; Gause-Nilsson, I. Long-term maintenance of efficacy of dapagliflozin in patients with type 2 diabetes mellitus and cardiovascular disease. *Diabetes Obes Metab* **2016**, *18*, 766-774, doi:10.1111/dom.12666.
122. Lepore, J.J.; Olson, E.; Demopoulos, L.; Haws, T.; Fang, Z.; Barbour, A.M.; Fossler, M.; Davila-Roman, V.G.; Russell, S.D.; Gropler, R.J. Effects of the Novel Long-Acting GLP-1 Agonist, Albiglutide, on Cardiac Function, Cardiac Metabolism, and Exercise Capacity in Patients With Chronic Heart Failure and Reduced Ejection Fraction. *JACC Heart Fail* **2016**, *4*, 559-566, doi:10.1016/j.jchf.2016.01.008.
123. Margulies, K.B.; Hernandez, A.F.; Redfield, M.M.; Givertz, M.M.; Oliveira, G.H.; Cole, R.; Mann, D.L.; Whellan, D.J.; Kiernan, M.S.; Felker, G.M.; et al. Effects of Liraglutide on Clinical Stability Among Patients With Advanced Heart Failure and Reduced Ejection Fraction: A Randomized Clinical Trial. *Jama* **2016**, *316*, 500-508, doi:10.1001/jama.2016.10260.
124. Mellander, A.; Billger, M.; Johnsson, E.; Traff, A.K.; Yoshida, S.; Johnsson, K. Hypersensitivity Events, Including Potentially Hypersensitivity-Related Skin Events, with Dapagliflozin in Patients with Type 2 Diabetes Mellitus: A Pooled Analysis. *Clinical drug investigation* **2016**, *36*, 925-933, doi:10.1007/s40261-016-0438-3.
125. Moses, R.G.; Round, E.; Shentu, Y.; Golm, G.T.; O'Neill E, A.; Gantz, I.; Engel, S.S.; Kaufman, K.D.; Goldstein, B.J. A randomized clinical trial evaluating the safety and efficacy of sitagliptin added to the combination of sulfonylurea and metformin in patients with type 2 diabetes

mellitus and inadequate glycemic control. *J Diabetes* **2016**, *8*, 701-711, doi:10.1111/1753-0407.12351.
126. Nauck, M.; Rizzo, M.; Johnson, A.; Bosch-Traberg, H.; Madsen, J.; Cariou, B. Once-Daily Liraglutide Versus Lixisenatide as Add-on to Metformin in Type 2 Diabetes: A 26-Week Randomized Controlled Clinical Trial. *Diabetes Care* **2016**, *39*, 1501-1509, doi:10.2337/dc15-2479.
127. Nauck, M.A.; Stewart, M.W.; Perkins, C.; Jones-Leone, A.; Yang, F.; Perry, C.; Reinhardt, R.R.; Rendell, M. Efficacy and safety of once-weekly GLP-1 receptor agonist albiglutide (HARMONY 2): 52 week primary endpoint results from a randomised, placebo-controlled trial in patients with type 2 diabetes mellitus inadequately controlled with diet and exercise. *Diabetologia* **2016**, *59*, 266-274, doi:10.1007/s00125-015-3795-1.
128. Zang, L.; Liu, Y.; Geng, J.; Luo, Y.; Bian, F.; Lv, X.; Yang, J.; Liu, J.; Peng, Y.; Li, Y.; et al. Efficacy and safety of liraglutide versus sitagliptin, both in combination with metformin, in Chinese patients with type 2 diabetes: a 26-week, open-label, randomized, active comparator clinical trial. *Diabetes Obes Metab* **2016**, *18*, 803-811, doi:10.1111/dom.12674.
129. Blonde, L.; Jendle, J.; Gross, J.; Woo, V.; Jiang, H.; Fahrbach, J.L.; Milicevic, Z. Once-weekly dulaglutide versus bedtime insulin glargine, both in combination with prandial insulin lispro, in patients with type 2 diabetes (AWARD-4): a randomised, open-label, phase 3, non-inferiority study. *Lancet* **2015**, *385*, 2057-2066, doi:10.1016/S0140-6736(15)60936-9.
130. Cefalu, W.T.; Leiter, L.A.; de Bruin, T.W.; Gause-Nilsson, I.; Sugg, J.; Parikh, S.J. Dapagliflozin's Effects on Glycemia and Cardiovascular Risk Factors in High-Risk Patients With Type 2 Diabetes: A 24-Week, Multicenter, Randomized, Double-Blind, Placebo-Controlled Study With a 28-Week Extension. *Diabetes Care* **2015**, *38*, 1218-1227, doi:10.2337/dc14-0315.
131. Davies, M.J.; Bergenstal, R.; Bode, B.; Kushner, R.F.; Lewin, A.; Skjoth, T.V.; Andreasen, A.H.; Jensen, C.B.; DeFronzo, R.A.; Group, N.N.S. Efficacy of Liraglutide for Weight Loss Among Patients With Type 2 Diabetes: The SCALE Diabetes Randomized Clinical Trial. *Jama* **2015**, *314*, 687-699, doi:10.1001/jama.2015.9676.
132. Giorgino, F.; Benroubi, M.; Sun, J.H.; Zimmermann, A.G.; Pechtner, V. Efficacy and Safety of Once-Weekly Dulaglutide Versus Insulin Glargine in Patients With Type 2 Diabetes on Metformin and Glimepiride (AWARD-2). *Diabetes Care* **2015**, *38*, 2241-2249, doi:10.2337/dc14-1625.

133. Hartley, P.; Shentu, Y.; Betz-Schiff, P.; Golm, G.T.; Sisk, C.M.; Engel, S.S.; Shankar, R.R. Efficacy and Tolerability of Sitagliptin Compared with Glimepiride in Elderly Patients with Type 2 Diabetes Mellitus and Inadequate Glycemic Control: A Randomized, Double-Blind, Non-Inferiority Trial. *Drugs Aging* **2015**, *32*, 469-476, doi:10.1007/s40266-015-0271-z.
134. Hirose, T.; Suzuki, M.; Tsumiyama, I. Efficacy and Safety of Vildagliptin as an Add-on to Insulin with or without Metformin in Japanese Patients with Type 2 Diabetes Mellitus: A 12-week, Double-Blind, Randomized Study. *Diabetes Ther* **2015**, *6*, 559-571, doi:10.1007/s13300-015-0147-6.
135. Ji, L.; Zinman, B.; Patel, S.; Ji, J.; Bailes, Z.; Thiemann, S.; Seck, T. Efficacy and safety of linagliptin co-administered with low-dose metformin once daily versus high-dose metformin twice daily in treatment-naive patients with type 2 diabetes: a double-blind randomized trial. *Adv Ther* **2015**, *32*, 201-215, doi:10.1007/s12325-015-0195-3.
136. Kovacs, C.S.; Seshiah, V.; Merker, L.; Christiansen, A.V.; Roux, F.; Salsali, A.; Kim, G.; Stella, P.; Woerle, H.J.; Broedl, U.C.; et al. Empagliflozin as Add-on Therapy to Pioglitazone With or Without Metformin in Patients With Type 2 Diabetes Mellitus. *Clin Ther* **2015**, *37*, 1773-1788 e1771, doi:10.1016/j.clinthera.2015.05.511.
137. Laakso, M.; Rosenstock, J.; Groop, P.H.; Barnett, A.H.; Gallwitz, B.; Hehnke, U.; Tamminen, I.; Patel, S.; von Eynatten, M.; Woerle, H.J. Treatment with the dipeptidyl peptidase-4 inhibitor linagliptin or placebo followed by glimepiride in patients with type 2 diabetes with moderate to severe renal impairment: a 52-week, randomized, double-blind clinical trial. *Diabetes Care* **2015**, *38*, e15-17, doi:10.2337/dc14-1684.
138. Mathieu, C.; Ranetti, A.E.; Li, D.; Ekholm, E.; Cook, W.; Hirshberg, B.; Chen, H.; Hansen, L.; Iqbal, N. Randomized, Double-Blind, Phase 3 Trial of Triple Therapy With Dapagliflozin Add-on to Saxagliptin Plus Metformin in Type 2 Diabetes. *Diabetes Care* **2015**, *38*, 2009-2017, doi:10.2337/dc15-0779.
139. Mathieu, C.; Shankar, R.R.; Lorber, D.; Umpierrez, G.; Wu, F.; Xu, L.; Golm, G.T.; Latham, M.; Kaufman, K.D.; Engel, S.S. A Randomized Clinical Trial to Evaluate the Efficacy and Safety of Co-Administration of Sitagliptin with Intensively Titrated Insulin Glargine. *Diabetes Ther* **2015**, *6*, 127-142, doi:10.1007/s13300-015-0105-3.
140. Matthaei, S.; Catrinoiu, D.; Celinski, A.; Ekholm, E.; Cook, W.; Hirshberg, B.; Chen, H.; Iqbal, N.; Hansen, L. Randomized, Double-Blind

Trial of Triple Therapy With Saxagliptin Add-on to Dapagliflozin Plus Metformin in Patients With Type 2 Diabetes. *Diabetes Care* **2015**, *38*, 2018-2024, doi:10.2337/dc15-0811.

141. Schernthaner, G.; Duran-Garcia, S.; Hanefeld, M.; Langslet, G.; Niskanen, L.; Ostgren, C.J.; Malvolti, E.; Hardy, E. Efficacy and tolerability of saxagliptin compared with glimepiride in elderly patients with type 2 diabetes: a randomized, controlled study (GENERATION). *Diabetes Obes Metab* **2015**, *17*, 630-638, doi:10.1111/dom.12461.
142. Sheu, W.H.; Gantz, I.; Chen, M.; Suryawanshi, S.; Mirza, A.; Goldstein, B.J.; Kaufman, K.D.; Engel, S.S. Safety and Efficacy of Omarigliptin (MK-3102), a Novel Once-Weekly DPP-4 Inhibitor for the Treatment of Patients With Type 2 Diabetes. *Diabetes Care* **2015**, *38*, 2106-2114, doi:10.2337/dc15-0109.
143. Bajaj, M.; Gilman, R.; Patel, S.; Kempthorne-Rawson, J.; Lewis-D'Agostino, D.; Woerle, H.J. Linagliptin improved glycaemic control without weight gain or hypoglycaemia in patients with type 2 diabetes inadequately controlled by a combination of metformin and pioglitazone: a 24-week randomized, double-blind study. *Diabet Med* **2014**, *31*, 1505-1514, doi:10.1111/dme.12495.
144. Barnett, A.H.; Mithal, A.; Manassie, J.; Jones, R.; Rattunde, H.; Woerle, H.J.; Broedl, U.C.; investigators, E.-R.R.t. Efficacy and safety of empagliflozin added to existing antidiabetes treatment in patients with type 2 diabetes and chronic kidney disease: a randomised, double-blind, placebo-controlled trial. *Lancet Diabetes Endocrinol* **2014**, *2*, 369-384, doi:10.1016/S2213-8587(13)70208-0.
145. Dungan, K.M.; Povedano, S.T.; Forst, T.; Gonzalez, J.G.; Atisso, C.; Sealls, W.; Fahrbach, J.L. Once-weekly dulaglutide versus once-daily liraglutide in metformin-treated patients with type 2 diabetes (AWARD-6): a randomised, open-label, phase 3, non-inferiority trial. *Lancet* **2014**, *384*, 1349-1357, doi:10.1016/S0140-6736(14)60976-4.
146. Henry, R.R.; Staels, B.; Fonseca, V.A.; Chou, M.Z.; Teng, R.; Golm, G.T.; Langdon, R.B.; Kaufman, K.D.; Steinberg, H.; Goldstein, B.J. Efficacy and safety of initial combination treatment with sitagliptin and pioglitazone--a factorial study. *Diabetes Obes Metab* **2014**, *16*, 223-230, doi:10.1111/dom.12194.
147. Kadowaki, T.; Kondo, K. Efficacy and safety of teneligliptin added to glimepiride in Japanese patients with type 2 diabetes mellitus: a randomized, double-blind, placebo-controlled study with an open-label, long-term extension. *Diabetes Obes Metab* **2014**, *16*, 418-425, doi:10.1111/dom.12235.

148. McGill, J.B.; Barnett, A.H.; Lewin, A.J.; Patel, S.; Neubacher, D.; von Eynatten, M.; Woerle, H.J. Linagliptin added to sulphonylurea in uncontrolled type 2 diabetes patients with moderate-to-severe renal impairment. *Diab Vasc Dis Res* **2014**, *11*, 34-40, doi:10.1177/1479164113507068.
149. Janssen Research & Development, L. The CANTATA-MSU Trial (CANagliflozin Treatment And Trial Analysis - Metformin and SUlphonylurea). Available online: https://clinicaltrials.gov/study/NCT01106625?cond=NCT01106625&rank=1 (accessed on 2024/12/18).
150. Pratley, R.E.; Fleck, P.; Wilson, C. Efficacy and safety of initial combination therapy with alogliptin plus metformin versus either as monotherapy in drug-naive patients with type 2 diabetes: a randomized, double-blind, 6-month study. *Diabetes Obes Metab* **2014**, *16*, 613-621, doi:10.1111/dom.12258.
151. Ridderstrale, M.; Andersen, K.R.; Zeller, C.; Kim, G.; Woerle, H.J.; Broedl, U.C.; investigators, E.-R.H.H.S.t. Comparison of empagliflozin and glimepiride as add-on to metformin in patients with type 2 diabetes: a 104-week randomised, active-controlled, double-blind, phase 3 trial. *Lancet Diabetes Endocrinol* **2014**, *2*, 691-700, doi:10.1016/S2213-8587(14)70120-2.
152. Rosenstock, J.; Fonseca, V.A.; Gross, J.L.; Ratner, R.E.; Ahren, B.; Chow, F.C.; Yang, F.; Miller, D.; Johnson, S.L.; Stewart, M.W.; et al. Advancing basal insulin replacement in type 2 diabetes inadequately controlled with insulin glargine plus oral agents: a comparison of adding albiglutide, a weekly GLP-1 receptor agonist, versus thrice-daily prandial insulin lispro. *Diabetes Care* **2014**, *37*, 2317-2325, doi:10.2337/dc14-0001.
153. Stenlof, K.; Cefalu, W.T.; Kim, K.A.; Jodar, E.; Alba, M.; Edwards, R.; Tong, C.; Canovatchel, W.; Meininger, G. Long-term efficacy and safety of canagliflozin monotherapy in patients with type 2 diabetes inadequately controlled with diet and exercise: findings from the 52-week CANTATA-M study. *Curr Med Res Opin* **2014**, *30*, 163-175, doi:10.1185/03007995.2013.850066.
154. Umpierrez, G.; Tofe Povedano, S.; Perez Manghi, F.; Shurzinske, L.; Pechtner, V. Efficacy and safety of dulaglutide monotherapy versus metformin in type 2 diabetes in a randomized controlled trial (AWARD-3). *Diabetes Care* **2014**, *37*, 2168-2176, doi:10.2337/dc13-2759.
155. Van Gaal, L.; Souhami, E.; Zhou, T.; Aronson, R. Efficacy and safety of the glucagon-like peptide-1 receptor agonist lixisenatide versus the dipeptidyl peptidase-4 inhibitor sitagliptin in young (<50 years) obese patients with type 2 diabetes mellitus. *J Clin Transl Endocrinol* **2014**, *1*, 31-37, doi:10.1016/j.jcte.2014.03.001.

156. Weissman, P.N.; Carr, M.C.; Ye, J.; Cirkel, D.T.; Stewart, M.; Perry, C.; Pratley, R. HARMONY 4: randomised clinical trial comparing once-weekly albiglutide and insulin glargine in patients with type 2 diabetes inadequately controlled with metformin with or without sulfonylurea. *Diabetologia* **2014**, *57*, 2475-2484, doi:10.1007/s00125-014-3360-3.
157. White, J.L.; Buchanan, P.; Li, J.; Frederich, R. A randomized controlled trial of the efficacy and safety of twice-daily saxagliptin plus metformin combination therapy in patients with type 2 diabetes and inadequate glycemic control on metformin monotherapy. *BMC Endocr Disord* **2014**, *14*, 17, doi:10.1186/1472-6823-14-17.
158. Alba, M.; Ahren, B.; Inzucchi, S.E.; Guan, Y.; Mallick, M.; Xu, L.; O'Neill, E.A.; Williams-Herman, D.E.; Kaufman, K.D.; Goldstein, B.J. Sitagliptin and pioglitazone provide complementary effects on postprandial glucose and pancreatic islet cell function. *Diabetes Obes Metab* **2013**, *15*, 1101-1110, doi:10.1111/dom.12145.
159. Arjona Ferreira, J.C.; Corry, D.; Mogensen, C.E.; Sloan, L.; Xu, L.; Golm, G.T.; Gonzalez, E.J.; Davies, M.J.; Kaufman, K.D.; Goldstein, B.J. Efficacy and safety of sitagliptin in patients with type 2 diabetes and ESRD receiving dialysis: a 54-week randomized trial. *Am J Kidney Dis* **2013**, *61*, 579-587, doi:10.1053/j.ajkd.2012.11.043.
160. Barnett, A.H.; Huisman, H.; Jones, R.; von Eynatten, M.; Patel, S.; Woerle, H.J. Linagliptin for patients aged 70 years or older with type 2 diabetes inadequately controlled with common antidiabetes treatments: a randomised, double-blind, placebo-controlled trial. *Lancet* **2013**, *382*, 1413-1423, doi:10.1016/S0140-6736(13)61500-7.
161. Barnett, A.H.; Charbonnel, B.; Li, J.; Donovan, M.; Fleming, D.; Iqbal, N. Saxagliptin add-on therapy to insulin with or without metformin for type 2 diabetes mellitus: 52-week safety and efficacy. *Clinical drug investigation* **2013**, *33*, 707-717, doi:10.1007/s40261-013-0107-8.
162. Charbonnel, B.; Steinberg, H.; Eymard, E.; Xu, L.; Thakkar, P.; Prabhu, V.; Davies, M.J.; Engel, S.S. Efficacy and safety over 26 weeks of an oral treatment strategy including sitagliptin compared with an injectable treatment strategy with liraglutide in patients with type 2 diabetes mellitus inadequately controlled on metformin: a randomised clinical trial. *Diabetologia* **2013**, *56*, 1503-1511, doi:10.1007/s00125-013-2905-1.
163. Dobs, A.S.; Goldstein, B.J.; Aschner, P.; Horton, E.S.; Umpierrez, G.E.; Duran, L.; Hill, J.S.; Chen, Y.; Golm, G.T.; Langdon, R.B.; et al. Efficacy and safety of sitagliptin added to ongoing metformin and rosiglitazone combination therapy in a randomized placebo-controlled 54-

week trial in patients with type 2 diabetes. *J Diabetes* **2013**, *5*, 68-79, doi:10.1111/j.1753-0407.2012.00223.x.
164. Ferrannini, E.; Berk, A.; Hantel, S.; Pinnetti, S.; Hach, T.; Woerle, H.J.; Broedl, U.C. Long-term safety and efficacy of empagliflozin, sitagliptin, and metformin: an active-controlled, parallel-group, randomized, 78-week open-label extension study in patients with type 2 diabetes. *Diabetes Care* **2013**, *36*, 4015-4021, doi:10.2337/dc13-0663.
165. Goke, B.; Gallwitz, B.; Eriksson, J.G.; Hellqvist, A.; Gause-Nilsson, I. Saxagliptin vs. glipizide as add-on therapy in patients with type 2 diabetes mellitus inadequately controlled on metformin alone: long-term (52-week) extension of a 52-week randomised controlled trial. *International journal of clinical practice* **2013**, *67*, 307-316, doi:10.1111/ijcp.12119.
166. Haak, T.; Meinicke, T.; Jones, R.; Weber, S.; von Eynatten, M.; Woerle, H.J. Initial combination of linagliptin and metformin in patients with type 2 diabetes: efficacy and safety in a randomised, double-blind 1-year extension study. *International journal of clinical practice* **2013**, *67*, 1283-1293, doi:10.1111/ijcp.12308.
167. Herold, K.C.; Gitelman, S.E.; Ehlers, M.R.; Gottlieb, P.A.; Greenbaum, C.J.; Hagopian, W.; Boyle, K.D.; Keyes-Elstein, L.; Aggarwal, S.; Phippard, D.; et al. Teplizumab (anti-CD3 mAb) treatment preserves C-peptide responses in patients with new-onset type 1 diabetes in a randomized controlled trial: metabolic and immunologic features at baseline identify a subgroup of responders. *Diabetes* **2013**, *62*, 3766-3774, doi:10.2337/db13-0345.
168. Herold, K.C.; Gitelman, S.E.; Willi, S.M.; Gottlieb, P.A.; Waldron-Lynch, F.; Devine, L.; Sherr, J.; Rosenthal, S.M.; Adi, S.; Jalaludin, M.Y.; et al. Teplizumab treatment may improve C-peptide responses in participants with type 1 diabetes after the new-onset period: a randomised controlled trial. *Diabetologia* **2013**, *56*, 391-400, doi:10.1007/s00125-012-2753-4.
169. Kadowaki, T.; Kondo, K. Efficacy, safety and dose-response relationship of teneligliptin, a dipeptidyl peptidase-4 inhibitor, in Japanese patients with type 2 diabetes mellitus. *Diabetes Obes Metab* **2013**, *15*, 810-818, doi:10.1111/dom.12092.
170. Philis-Tsimikas, A.; Del Prato, S.; Satman, I.; Bhargava, A.; Dharmalingam, M.; Skjoth, T.V.; Rasmussen, S.; Garber, A.J. Effect of insulin degludec versus sitagliptin in patients with type 2 diabetes uncontrolled on oral antidiabetic agents. *Diabetes Obes Metab* **2013**, *15*, 760-766, doi:10.1111/dom.12115.
171. Roden, M.; Weng, J.; Eilbracht, J.; Delafont, B.; Kim, G.; Woerle, H.J.; Broedl, U.C.; investigators, E.-R.M.t. Empagliflozin monotherapy

with sitagliptin as an active comparator in patients with type 2 diabetes: a randomised, double-blind, placebo-controlled, phase 3 trial. *Lancet Diabetes Endocrinol* **2013**, *1*, 208-219, doi:10.1016/S2213-8587(13)70084-6.

172. Rosenstock, J.; Seman, L.J.; Jelaska, A.; Hantel, S.; Pinnetti, S.; Hach, T.; Woerle, H.J. Efficacy and safety of empagliflozin, a sodium glucose cotransporter 2 (SGLT2) inhibitor, as add-on to metformin in type 2 diabetes with mild hyperglycaemia. *Diabetes Obes Metab* **2013**, *15*, 1154-1160, doi:10.1111/dom.12185.
173. Rosenstock, J.; Gross, J.L.; Aguilar-Salinas, C.; Hissa, M.; Berglind, N.; Ravichandran, S.; Fleming, D. Long-term 4-year safety of saxagliptin in drug-naive and metformin-treated patients with Type 2 diabetes. *Diabet Med* **2013**, *30*, 1472-1476, doi:10.1111/dme.12267.
174. Rosenstock, J.; Wilson, C.; Fleck, P. Alogliptin versus glipizide monotherapy in elderly type 2 diabetes mellitus patients with mild hyperglycaemia: a prospective, double-blind, randomized, 1-year study. *Diabetes Obes Metab* **2013**, *15*, 906-914, doi:10.1111/dom.12102.
175. Schernthaner, G.; Gross, J.L.; Rosenstock, J.; Guarisco, M.; Fu, M.; Yee, J.; Kawaguchi, M.; Canovatchel, W.; Meininger, G. Canagliflozin compared with sitagliptin for patients with type 2 diabetes who do not have adequate glycemic control with metformin plus sulfonylurea: a 52-week randomized trial. *Diabetes Care* **2013**, *36*, 2508-2515, doi:10.2337/dc12-2491.
176. Barnett, A.H.; Patel, S.; Harper, R.; Toorawa, R.; Thiemann, S.; von Eynatten, M.; Woerle, H.J. Linagliptin monotherapy in type 2 diabetes patients for whom metformin is inappropriate: an 18-week randomized, double-blind, placebo-controlled phase III trial with a 34-week active-controlled extension. *Diabetes Obes Metab* **2012**, *14*, 1145-1154, doi:10.1111/dom.12011.
177. DeFronzo, R.A.; Burant, C.F.; Fleck, P.; Wilson, C.; Mekki, Q.; Pratley, R.E. Efficacy and tolerability of the DPP-4 inhibitor alogliptin combined with pioglitazone, in metformin-treated patients with type 2 diabetes. *J Clin Endocrinol Metab* **2012**, *97*, 1615-1622, doi:10.1210/jc.2011-2243.
178. Frederich, R.; McNeill, R.; Berglind, N.; Fleming, D.; Chen, R. The efficacy and safety of the dipeptidyl peptidase-4 inhibitor saxagliptin in treatment-naive patients with type 2 diabetes mellitus: a randomized controlled trial. *Diabetol Metab Syndr* **2012**, *4*, 36, doi:10.1186/1758-5996-4-36.
179. Gallwitz, B.; Guzman, J.; Dotta, F.; Guerci, B.; Simo, R.; Basson, B.R.; Festa, A.; Kiljanski, J.; Sapin, H.; Trautmann, M.; et al. Exenatide

twice daily versus glimepiride for prevention of glycaemic deterioration in patients with type 2 diabetes with metformin failure (EUREXA): an open-label, randomised controlled trial. *Lancet* **2012**, *379*, 2270-2278, doi:10.1016/S0140-6736(12)60479-6.
180. Haak, T.; Meinicke, T.; Jones, R.; Weber, S.; von Eynatten, M.; Woerle, H.J. Initial combination of linagliptin and metformin improves glycaemic control in type 2 diabetes: a randomized, double-blind, placebo-controlled study. *Diabetes Obes Metab* **2012**, *14*, 565-574, doi:10.1111/j.1463-1326.2012.01590.x.
181. Hermans, M.P.; Delibasi, T.; Farmer, I.; Lohm, L.; Maheux, P.; Piatti, P.; Malvolti, E.; Jorgens, S.; Charbonnel, B. Effects of saxagliptin added to sub-maximal doses of metformin compared with uptitration of metformin in type 2 diabetes: the PROMPT study. *Curr Med Res Opin* **2012**, *28*, 1635-1645, doi:10.1185/03007995.2012.735646.
182. Lewin, A.J.; Arvay, L.; Liu, D.; Patel, S.; von Eynatten, M.; Woerle, H.J. Efficacy and tolerability of linagliptin added to a sulfonylurea regimen in patients with inadequately controlled type 2 diabetes mellitus: an 18-week, multicenter, randomized, double-blind, placebo-controlled trial. *Clin Ther* **2012**, *34*, 1909-1919 e1915, doi:10.1016/j.clinthera.2012.07.008.
183. Pan, C.Y.; Yang, W.; Tou, C.; Gause-Nilsson, I.; Zhao, J. Efficacy and safety of saxagliptin in drug-naive Asian patients with type 2 diabetes mellitus: a randomized controlled trial. *Diabetes/metabolism research and reviews* **2012**, *28*, 268-275, doi:10.1002/dmrr.1306.
184. Rosenstock, J.; Aggarwal, N.; Polidori, D.; Zhao, Y.; Arbit, D.; Usiskin, K.; Capuano, G.; Canovatchel, W.; Canagliflozin, D.I.A.S.G. Dose-ranging effects of canagliflozin, a sodium-glucose cotransporter 2 inhibitor, as add-on to metformin in subjects with type 2 diabetes. *Diabetes Care* **2012**, *35*, 1232-1238, doi:10.2337/dc11-1926.
185. Ross, S.A.; Rafeiro, E.; Meinicke, T.; Toorawa, R.; Weber-Born, S.; Woerle, H.J. Efficacy and safety of linagliptin 2.5 mg twice daily versus 5 mg once daily in patients with type 2 diabetes inadequately controlled on metformin: a randomised, double-blind, placebo-controlled trial. *Curr Med Res Opin* **2012**, *28*, 1465-1474, doi:10.1185/03007995.2012.714360.
186. Seino, Y.; Hiroi, S.; Hirayama, M.; Kaku, K. Efficacy and safety of alogliptin added to sulfonylurea in Japanese patients with type 2 diabetes: A randomized, double-blind, placebo-controlled trial with an open-label, long-term extension study. *J Diabetes Investig* **2012**, *3*, 517-525, doi:10.1111/j.2040-1124.2012.00226.x.
187. Seino, Y.; Miyata, Y.; Hiroi, S.; Hirayama, M.; Kaku, K. Efficacy and safety of alogliptin added to metformin in Japanese patients with type

2 diabetes: a randomized, double-blind, placebo-controlled trial with an open-label, long-term extension study. *Diabetes Obes Metab* **2012**, *14*, 927-936, doi:10.1111/j.1463-1326.2012.01620.x.
188. Wilding, J.P.; Woo, V.; Soler, N.G.; Pahor, A.; Sugg, J.; Rohwedder, K.; Parikh, S.; Dapagliflozin 006 Study, G. Long-term efficacy of dapagliflozin in patients with type 2 diabetes mellitus receiving high doses of insulin: a randomized trial. *Ann Intern Med* **2012**, *156*, 405-415, doi:10.7326/0003-4819-156-6-201203200-00003.
189. Yang, W.; Guan, Y.; Shentu, Y.; Li, Z.; Johnson-Levonas, A.O.; Engel, S.S.; Kaufman, K.D.; Goldstein, B.J.; Alba, M. The addition of sitagliptin to ongoing metformin therapy significantly improves glycemic control in Chinese patients with type 2 diabetes. *J Diabetes* **2012**, *4*, 227-237, doi:10.1111/j.1753-0407.2012.00213.x.
190. Barzilai, N.; Guo, H.; Mahoney, E.M.; Caporossi, S.; Golm, G.T.; Langdon, R.B.; Williams-Herman, D.; Kaufman, K.D.; Amatruda, J.M.; Goldstein, B.J.; et al. Efficacy and tolerability of sitagliptin monotherapy in elderly patients with type 2 diabetes: a randomized, double-blind, placebo-controlled trial. *Curr Med Res Opin* **2011**, *27*, 1049-1058, doi:10.1185/03007995.2011.568059.
191. Gallwitz, B.; Bohmer, M.; Segiet, T.; Molle, A.; Milek, K.; Becker, B.; Helsberg, K.; Petto, H.; Peters, N.; Bachmann, O. Exenatide twice daily versus premixed insulin aspart 70/30 in metformin-treated patients with type 2 diabetes: a randomized 26-week study on glycemic control and hypoglycemia. *Diabetes Care* **2011**, *34*, 604-606, doi:10.2337/dc10-1900.
192. Gomis, R.; Espadero, R.M.; Jones, R.; Woerle, H.J.; Dugi, K.A. Efficacy and safety of initial combination therapy with linagliptin and pioglitazone in patients with inadequately controlled type 2 diabetes: a randomized, double-blind, placebo-controlled study. *Diabetes Obes Metab* **2011**, *13*, 653-661, doi:10.1111/j.1463-1326.2011.01391.x.
193. Hollander, P.L.; Li, J.; Frederich, R.; Allen, E.; Chen, R.; Investigators, C.V. Safety and efficacy of saxagliptin added to thiazolidinedione over 76 weeks in patients with type 2 diabetes mellitus. *Diab Vasc Dis Res* **2011**, *8*, 125-135, doi:10.1177/1479164111404575.
194. Nowicki, M.; Rychlik, I.; Haller, H.; Warren, M.L.; Suchower, L.; Gause-Nilsson, I.; Investigators, D.C. Saxagliptin improves glycaemic control and is well tolerated in patients with type 2 diabetes mellitus and renal impairment. *Diabetes Obes Metab* **2011**, *13*, 523-532, doi:10.1111/j.1463-1326.2011.01382.x.
195. Owens, D.R.; Swallow, R.; Dugi, K.A.; Woerle, H.J. Efficacy and safety of linagliptin in persons with type 2 diabetes inadequately

controlled by a combination of metformin and sulphonylurea: a 24-week randomized study. *Diabet Med* **2011**, *28*, 1352-1361, doi:10.1111/j.1464-5491.2011.03387.x.
196. Reasner, C.; Olansky, L.; Seck, T.L.; Williams-Herman, D.E.; Chen, M.; Terranella, L.; Johnson-Levonas, A.O.; Kaufman, K.D.; Goldstein, B.J. The effect of initial therapy with the fixed-dose combination of sitagliptin and metformin compared with metformin monotherapy in patients with type 2 diabetes mellitus. *Diabetes Obes Metab* **2011**, *13*, 644-652, doi:10.1111/j.1463-1326.2011.01390.x.
197. Seino, Y.; Fujita, T.; Hiroi, S.; Hirayama, M.; Kaku, K. Alogliptin plus voglibose in Japanese patients with type 2 diabetes: a randomized, double-blind, placebo-controlled trial with an open-label, long-term extension. *Curr Med Res Opin* **2011**, *27 Suppl 3*, 21-29, doi:10.1185/03007995.2011.614936.
198. Yang, W.; Pan, C.Y.; Tou, C.; Zhao, J.; Gause-Nilsson, I. Efficacy and safety of saxagliptin added to metformin in Asian people with type 2 diabetes mellitus: a randomized controlled trial. *Diabetes Res Clin Pract* **2011**, *94*, 217-224, doi:10.1016/j.diabres.2011.07.035.
199. Bailey, C.J.; Gross, J.L.; Pieters, A.; Bastien, A.; List, J.F. Effect of dapagliflozin in patients with type 2 diabetes who have inadequate glycaemic control with metformin: a randomised, double-blind, placebo-controlled trial. *Lancet* **2010**, *375*, 2223-2233, doi:10.1016/S0140-6736(10)60407-2.
200. Bergenstal, R.M.; Wysham, C.; Macconell, L.; Malloy, J.; Walsh, B.; Yan, P.; Wilhelm, K.; Malone, J.; Porter, L.E.; Group, D.-S. Efficacy and safety of exenatide once weekly versus sitagliptin or pioglitazone as an adjunct to metformin for treatment of type 2 diabetes (DURATION-2): a randomised trial. *Lancet* **2010**, *376*, 431-439, doi:10.1016/S0140-6736(10)60590-9.
201. Trials, G.C. A Study of the Efficacy and Safety of Albiglutide in Subjects With Type 2 Diabetes With Renal Impairment (NCT01098539). Available online: https://clinicaltrials.gov/study/NCT01098539?cond=NCT01098539&rank=1 (accessed on 2025/7/22).
202. Iwamoto, Y.; Taniguchi, T.; Nonaka, K.; Okamoto, T.; Okuyama, K.; Arjona Ferreira, J.C.; Amatruda, J. Dose-ranging efficacy of sitagliptin, a dipeptidyl peptidase-4 inhibitor, in Japanese patients with type 2 diabetes mellitus. *Endocr J* **2010**, *57*, 383-394, doi:10.1507/endocrj.k09e-272.
203. Rosenstock, J.; Inzucchi, S.E.; Seufert, J.; Fleck, P.R.; Wilson, C.A.; Mekki, Q. Initial combination therapy with alogliptin and pioglitazone in drug-naive patients with type 2 diabetes. *Diabetes Care* **2010**, *33*, 2406-2408, doi:10.2337/dc10-0159.

204. Vilsboll, T.; Rosenstock, J.; Yki-Jarvinen, H.; Cefalu, W.T.; Chen, Y.; Luo, E.; Musser, B.; Andryuk, P.J.; Ling, Y.; Kaufman, K.D.; et al. Efficacy and safety of sitagliptin when added to insulin therapy in patients with type 2 diabetes. *Diabetes Obes Metab* **2010**, *12*, 167-177, doi:10.1111/j.1463-1326.2009.01173.x.
205. Buse, J.B.; Rosenstock, J.; Sesti, G.; Schmidt, W.E.; Montanya, E.; Brett, J.H.; Zychma, M.; Blonde, L.; Group, L.-S. Liraglutide once a day versus exenatide twice a day for type 2 diabetes: a 26-week randomised, parallel-group, multinational, open-label trial (LEAD-6). *Lancet* **2009**, *374*, 39-47, doi:10.1016/S0140-6736(09)60659-0.
206. Lilly, E. Protege Encore Study- Clinical Trial of Teplizumab (MGA031) in Children and Adults With Recent-Onset Type 1 Diabetes Mellitus (Protege Encore Study). Available online: https://clinicaltrials.gov/study/NCT00920582?cond=NCT00920582&rank=1 (accessed on 2025/05/15).
207. Garber, A.; Henry, R.; Ratner, R.; Garcia-Hernandez, P.A.; Rodriguez-Pattzi, H.; Olvera-Alvarez, I.; Hale, P.M.; Zdravkovic, M.; Bode, B.; Group, L.-S. Liraglutide versus glimepiride monotherapy for type 2 diabetes (LEAD-3 Mono): a randomised, 52-week, phase III, double-blind, parallel-treatment trial. *Lancet* **2009**, *373*, 473-481, doi:10.1016/S0140-6736(08)61246-5.
208. Jadzinsky, M.; Pfutzner, A.; Paz-Pacheco, E.; Xu, Z.; Allen, E.; Chen, R.; Investigators, C.V. Saxagliptin given in combination with metformin as initial therapy improves glycaemic control in patients with type 2 diabetes compared with either monotherapy: a randomized controlled trial. *Diabetes Obes Metab* **2009**, *11*, 611-622, doi:10.1111/j.1463-1326.2009.01056.x.
209. Nauck, M.; Frid, A.; Hermansen, K.; Shah, N.S.; Tankova, T.; Mitha, I.H.; Zdravkovic, M.; During, M.; Matthews, D.R.; Group, L.-S. Efficacy and safety comparison of liraglutide, glimepiride, and placebo, all in combination with metformin, in type 2 diabetes: the LEAD (liraglutide effect and action in diabetes)-2 study. *Diabetes Care* **2009**, *32*, 84-90, doi:10.2337/dc08-1355.
210. Nauck, M.A.; Ellis, G.C.; Fleck, P.R.; Wilson, C.A.; Mekki, Q.; Alogliptin Study, G. Efficacy and safety of adding the dipeptidyl peptidase-4 inhibitor alogliptin to metformin therapy in patients with type 2 diabetes inadequately controlled with metformin monotherapy: a multicentre, randomised, double-blind, placebo-controlled study. *International journal of clinical practice* **2009**, *63*, 46-55, doi:10.1111/j.1742-1241.2008.01933.x.
211. Pratley, R.E.; Kipnes, M.S.; Fleck, P.R.; Wilson, C.; Mekki, Q.; Alogliptin Study, G. Efficacy and safety of the dipeptidyl peptidase-4

inhibitor alogliptin in patients with type 2 diabetes inadequately controlled by glyburide monotherapy. *Diabetes Obes Metab* **2009**, *11*, 167-176, doi:10.1111/j.1463-1326.2008.01016.x.
212. Pratley, R.E.; Reusch, J.E.; Fleck, P.R.; Wilson, C.A.; Mekki, Q.; Alogliptin Study, G. Efficacy and safety of the dipeptidyl peptidase-4 inhibitor alogliptin added to pioglitazone in patients with type 2 diabetes: a randomized, double-blind, placebo-controlled study. *Curr Med Res Opin* **2009**, *25*, 2361-2371, doi:10.1185/03007990903156111.
213. Rosenstock, J.; Rendell, M.S.; Gross, J.L.; Fleck, P.R.; Wilson, C.A.; Mekki, Q. Alogliptin added to insulin therapy in patients with type 2 diabetes reduces HbA(1C) without causing weight gain or increased hypoglycaemia. *Diabetes Obes Metab* **2009**, *11*, 1145-1152, doi:10.1111/j.1463-1326.2009.01124.x.
214. DeFronzo, R.A.; Fleck, P.R.; Wilson, C.A.; Mekki, Q.; Alogliptin Study, G. Efficacy and safety of the dipeptidyl peptidase-4 inhibitor alogliptin in patients with type 2 diabetes and inadequate glycemic control: a randomized, double-blind, placebo-controlled study. *Diabetes Care* **2008**, *31*, 2315-2317, doi:10.2337/dc08-1035.
215. Raz, I.; Chen, Y.; Wu, M.; Hussain, S.; Kaufman, K.D.; Amatruda, J.M.; Langdon, R.B.; Stein, P.P.; Alba, M. Efficacy and safety of sitagliptin added to ongoing metformin therapy in patients with type 2 diabetes. *Curr Med Res Opin* **2008**, *24*, 537-550, doi:10.1185/030079908x260925.
216. Goldstein, B.J.; Feinglos, M.N.; Lunceford, J.K.; Johnson, J.; Williams-Herman, D.E.; Sitagliptin 036 Study, G. Effect of initial combination therapy with sitagliptin, a dipeptidyl peptidase-4 inhibitor, and metformin on glycemic control in patients with type 2 diabetes. *Diabetes Care* **2007**, *30*, 1979-1987, doi:10.2337/dc07-0627.
217. Charbonnel, B.; Karasik, A.; Liu, J.; Wu, M.; Meininger, G.; Sitagliptin Study, G. Efficacy and safety of the dipeptidyl peptidase-4 inhibitor sitagliptin added to ongoing metformin therapy in patients with type 2 diabetes inadequately controlled with metformin alone. *Diabetes Care* **2006**, *29*, 2638-2643, doi:10.2337/dc06-0706.
218. Rosenstock, J.; Brazg, R.; Andryuk, P.J.; Lu, K.; Stein, P.; Sitagliptin Study, G. Efficacy and safety of the dipeptidyl peptidase-4 inhibitor sitagliptin added to ongoing pioglitazone therapy in patients with type 2 diabetes: a 24-week, multicenter, randomized, double-blind, placebo-controlled, parallel-group study. *Clin Ther* **2006**, *28*, 1556-1568, doi:10.1016/j.clinthera.2006.10.007.

219. Akasaka, H.; Sugimoto, K.; Shintani, A.; Taniuchi, S.; Yamamoto, K.; Iwakura, K.; Okamura, A.; Takiuchi, S.; Fukuda, M.; Kamide, K.; et al. Effects of ipragliflozin on left ventricular diastolic function in patients with type 2 diabetes and heart failure with preserved ejection fraction: The EXCEED randomized controlled multicenter study. *Geriatr Gerontol Int* **2022**, *22*, 298-304, doi:10.1111/ggi.14363.
220. Arturi, F.; Succurro, E.; Miceli, S.; Cloro, C.; Ruffo, M.; Maio, R.; Perticone, M.; Sesti, G.; Perticone, F. Liraglutide improves cardiac function in patients with type 2 diabetes and chronic heart failure. *Endocrine* **2017**, *57*, 464-473, doi:10.1007/s12020-016-1166-4.
221. Bailey, T.S.; Takacs, R.; Tinahones, F.J.; Rao, P.V.; Tsoukas, G.M.; Thomsen, A.B.; Kaltoft, M.S.; Maislos, M. Efficacy and safety of switching from sitagliptin to liraglutide in subjects with type 2 diabetes (LIRA-SWITCH): a randomized, double-blind, double-dummy, active-controlled 26-week trial. *Diabetes Obes Metab* **2016**, *18*, 1191-1198, doi:10.1111/dom.12736.
222. Bergenstal, R.M.; Forti, A.; Chiasson, J.L.; Woloschak, M.; Boldrin, M.; Balena, R. Efficacy and safety of taspoglutide versus sitagliptin for type 2 diabetes mellitus (T-emerge 4 trial). *Diabetes Ther* **2012**, *3*, 13, doi:10.1007/s13300-012-0013-8.
223. Blonde, L.; Dagogo-Jack, S.; Banerji, M.A.; Pratley, R.E.; Marcellari, A.; Braceras, R.; Purkayastha, D.; Baron, M. Comparison of vildagliptin and thiazolidinedione as add-on therapy in patients inadequately controlled with metformin: results of the GALIANT trial--a primary care, type 2 diabetes study. *Diabetes Obes Metab* **2009**, *11*, 978-986, doi:10.1111/j.1463-1326.2009.01080.x.
224. Bosi, E.; Camisasca, R.P.; Collober, C.; Rochotte, E.; Garber, A.J. Effects of vildagliptin on glucose control over 24 weeks in patients with type 2 diabetes inadequately controlled with metformin. *Diabetes Care* **2007**, *30*, 890-895, doi:10.2337/dc06-1732.
225. Bosi, E.; Dotta, F.; Jia, Y.; Goodman, M. Vildagliptin plus metformin combination therapy provides superior glycaemic control to individual monotherapy in treatment-naive patients with type 2 diabetes mellitus. *Diabetes Obes Metab* **2009**, *11*, 506-515, doi:10.1111/j.1463-1326.2009.01040.x.
226. Carbone, S.; Billingsley, H.E.; Canada, J.M.; Bressi, E.; Rotelli, B.; Kadariya, D.; Dixon, D.L.; Markley, R.; Trankle, C.R.; Cooke, R.; et al. The effects of canagliflozin compared to sitagliptin on cardiorespiratory fitness in type 2 diabetes mellitus and heart failure with reduced ejection fraction: The CANA-HF study. *Diabetes/metabolism research and reviews* **2020**, *36*, e3335, doi:10.1002/dmrr.3335.
227. Chan, J.C.; Scott, R.; Arjona Ferreira, J.C.; Sheng, D.; Gonzalez, E.; Davies, M.J.; Stein, P.P.; Kaufman, K.D.; Amatruda, J.M.; Williams-Herman, D. Safety and efficacy of sitagliptin in patients with type 2 diabetes and chronic renal insufficiency. *Diabetes Obes Metab* **2008**,

10, 545-555, doi:10.1111/j.1463-1326.2008.00914.x.

228. Chow, E.; Wang, K.; Lim, C.K.P.; Tsoi, S.T.F.; Fan, B.; Poon, E.; Luk, A.O.Y.; Ma, R.C.W.; Ferrannini, E.; Mari, A.; et al. Dorzagliatin, a Dual-Acting Glucokinase Activator, Increases Insulin Secretion and Glucose Sensitivity in Glucokinase Maturity-Onset Diabetes of the Young and Recent-Onset Type 2 Diabetes. *Diabetes* **2023**, *72*, 299-308, doi:10.2337/db22-0708.

229. Dayem, K.A.; Younis, O.; Zarif, B.; Attia, S.; AbdelSalam, A. Impact of dapagliflozin on cardiac function following anterior myocardial infarction in non-diabetic patients - DACAMI (a randomized controlled clinical trial). *Int J Cardiol* **2023**, *379*, 9-14, doi:10.1016/j.ijcard.2023.03.002.

230. de Boer, S.A.; Heerspink, H.J.L.; Juarez Orozco, L.E.; van Roon, A.M.; Kamphuisen, P.W.; Smit, A.J.; Slart, R.; Lefrandt, J.D.; Mulder, D.J. Effect of linagliptin on pulse wave velocity in early type 2 diabetes: A randomized, double-blind, controlled 26-week trial (RELEASE). *Diabetes Obes Metab* **2017**, *19*, 1147-1154, doi:10.1111/dom.12925.

231. Dei Cas, A.; Micheli, M.M.; Aldigeri, R.; Gardini, S.; Ferrari-Pellegrini, F.; Perini, M.; Messa, G.; Antonini, M.; Spigoni, V.; Cinquegrani, G.; et al. Long-acting exenatide does not prevent cognitive decline in mild cognitive impairment: a proof-of-concept clinical trial. *Journal of endocrinological investigation* **2024**, *47*, 2339-2349, doi:10.1007/s40618-024-02320-7.

232. Dubourg, J.; Perrimond-Dauchy, S.; Felices, M.; Bolze, S.; Voiriot, P.; Fouqueray, P. Absence of QTc prolongation in a thorough QT study with imeglimin, a first in class oral agent for type 2 diabetes mellitus. *Eur J Clin Pharmacol* **2020**, *76*, 1393-1400, doi:10.1007/s00228-020-02929-6.

233. Dubourg, J.; Ueki, K.; Grouin, J.M.; Fouqueray, P. Efficacy and safety of imeglimin in Japanese patients with type 2 diabetes: A 24-week, randomized, double-blind, placebo-controlled, dose-ranging phase 2b trial. *Diabetes Obes Metab* **2021**, *23*, 800-810, doi:10.1111/dom.14285.

234. Dubourg, J.; Fouqueray, P.; Thang, C.; Grouin, J.M.; Ueki, K. Efficacy and Safety of Imeglimin Monotherapy Versus Placebo in Japanese Patients With Type 2 Diabetes (TIMES 1): A Double-Blind, Randomized, Placebo-Controlled, Parallel-Group, Multicenter Phase 3 Trial. *Diabetes Care* **2021**, *44*, 952-959, doi:10.2337/dc20-0763.

235. Ejiri, K.; Miyoshi, T.; Kihara, H.; Hata, Y.; Nagano, T.; Takaishi, A.; Toda, H.; Nanba, S.; Nakamura, Y.; Akagi, S.; et al. Effect of Luseogliflozin

on Heart Failure With Preserved Ejection Fraction in Patients With Diabetes Mellitus. *J Am Heart Assoc* **2020**, *9*, e015103, doi:10.1161/JAHA.119.015103.
236. Fonseca, V.; Schweizer, A.; Albrecht, D.; Baron, M.A.; Chang, I.; Dejager, S. Addition of vildagliptin to insulin improves glycaemic control in type 2 diabetes. *Diabetologia* **2007**, *50*, 1148-1155, doi:10.1007/s00125-007-0633-0.
237. Fouqueray, P.; Pirags, V.; Inzucchi, S.E.; Bailey, C.J.; Schernthaner, G.; Diamant, M.; Lebovitz, H.E. The efficacy and safety of imeglimin as add-on therapy in patients with type 2 diabetes inadequately controlled with metformin monotherapy. *Diabetes Care* **2013**, *36*, 565-568, doi:10.2337/dc12-0453.
238. Fouqueray, P.; Pirags, V.; Diamant, M.; Schernthaner, G.; Lebovitz, H.E.; Inzucchi, S.E.; Bailey, C.J. The efficacy and safety of imeglimin as add-on therapy in patients with type 2 diabetes inadequately controlled with sitagliptin monotherapy. *Diabetes Care* **2014**, *37*, 1924-1930, doi:10.2337/dc13-2349.
239. Garber, A.J.; Schweizer, A.; Baron, M.A.; Rochotte, E.; Dejager, S. Vildagliptin in combination with pioglitazone improves glycaemic control in patients with type 2 diabetes failing thiazolidinedione monotherapy: a randomized, placebo-controlled study. *Diabetes Obes Metab* **2007**, *9*, 166-174, doi:10.1111/j.1463-1326.2006.00684.x.
240. Garber, A.J.; Foley, J.E.; Banerji, M.A.; Ebeling, P.; Gudbjornsdottir, S.; Camisasca, R.P.; Couturier, A.; Baron, M.A. Effects of vildagliptin on glucose control in patients with type 2 diabetes inadequately controlled with a sulphonylurea. *Diabetes Obes Metab* **2008**, *10*, 1047-1056, doi:10.1111/j.1463-1326.2008.00859.x.
241. Gu, T.; Ma, J.; Zhang, Q.; Zhu, L.; Zhang, H.; Xu, L.; Cheng, J.; Shi, B.; Li, D.; Shao, J.; et al. Comparative effect of saxagliptin and glimepiride with a composite endpoint of adequate glycaemic control without hypoglycaemia and without weight gain in patients uncontrolled with metformin therapy: Results from the SPECIFY study, a 48-week, multi-centre, randomized, controlled trial. *Diabetes Obes Metab* **2019**, *21*, 939-948, doi:10.1111/dom.13605.
242. Hagi, K.; Kochi, K.; Watada, H.; Kaku, K.; Ueki, K. Effect of patient characteristics on the efficacy and safety of imeglimin monotherapy in Japanese patients with type 2 diabetes mellitus: A post-hoc analysis of two randomized, placebo-controlled trials. *J Diabetes Investig* **2023**, *14*, 1101-1109, doi:10.1111/jdi.14035.

243. Hagi, K.; Kochi, K.; Watada, H.; Kaku, K.; Ueki, K. Factors contributing to the clinical effectiveness of imeglimin monotherapy in Japanese patients with type 2 diabetes mellitus. *J Diabetes Investig* **2024**, *15*, 1239-1247, doi:10.1111/jdi.14247.
244. Hagi, K.; Kochi, K.; Watada, H.; Kaku, K.; Ueki, K. Differences in imeglimin response in subgroups of patients with type 2 diabetes stratified by data-driven cluster analysis: A post-hoc analysis of imeglimin clinical trial data. *Diabetes Obes Metab* **2024**, *26*, 3732-3742, doi:10.1111/dom.15716.
245. Hanefeld, M.; Herman, G.A.; Wu, M.; Mickel, C.; Sanchez, M.; Stein, P.P.; Sitagliptin Study, I. Once-daily sitagliptin, a dipeptidyl peptidase-4 inhibitor, for the treatment of patients with type 2 diabetes. *Curr Med Res Opin* **2007**, *23*, 1329-1339, doi:10.1185/030079907X188152.
246. Hao, Z.; Zhang, Y. Different Doses of Empagliflozin in Patients with Heart Failure with Reduced Ejection Fraction. *Int Heart J* **2022**, *63*, 852-856, doi:10.1536/ihj.22-151.
247. Henry, R.R.; Smith, S.R.; Schwartz, S.L.; Mudaliar, S.R.; Deacon, C.F.; Holst, J.J.; Duan, R.Y.; Chen, R.S.; List, J.F. Effects of saxagliptin on beta-cell stimulation and insulin secretion in patients with type 2 diabetes. *Diabetes Obes Metab* **2011**, *13*, 850-858, doi:10.1111/j.1463-1326.2011.01417.x.
248. Hermansen, K.; Kipnes, M.; Luo, E.; Fanurik, D.; Khatami, H.; Stein, P.; Sitagliptin Study, G. Efficacy and safety of the dipeptidyl peptidase-4 inhibitor, sitagliptin, in patients with type 2 diabetes mellitus inadequately controlled on glimepiride alone or on glimepiride and metformin. *Diabetes Obes Metab* **2007**, *9*, 733-745, doi:10.1111/j.1463-1326.2007.00744.x.
249. Hollander, P.; Li, J.; Allen, E.; Chen, R.; Investigators, C.V. Saxagliptin added to a thiazolidinedione improves glycemic control in patients with type 2 diabetes and inadequate control on thiazolidinedione alone. *J Clin Endocrinol Metab* **2009**, *94*, 4810-4819, doi:10.1210/jc.2009-0550.
250. Ito, J.; Hagi, K.; Kochi, K.; Ueki, K.; Watada, H.; Kaku, K. Gastrointestinal symptoms in patients receiving imeglimin in combination with metformin: A post-hoc analysis of imeglimin clinical trial data. *J Diabetes Investig* **2025**, *16*, 629-638, doi:10.1111/jdi.14396.
251. Ji, L.; Li, L.; Kuang, J.; Yang, T.; Kim, D.J.; Kadir, A.A.; Huang, C.N.; Lee, D. Efficacy and safety of fixed-dose combination therapy, alogliptin plus metformin, in Asian patients with type 2 diabetes: A phase 3 trial. *Diabetes Obes Metab* **2017**, *19*, 754-758,

doi:10.1111/dom.12875.
252. Kaku, K.; Shimoda, M.; Osonoi, T.; Iwamoto, M.; Kaneto, H. Efficacy and safety of imeglimin add-on to DPP-4 inhibitor therapy in Japanese patients with type 2 diabetes mellitus: An interim analysis of the randomised, double-blind FAMILIAR trial. *Diabetes Obes Metab* **2025**, *27*, 3212-3222, doi:10.1111/dom.16336.
253. Kanazawa, I.; Tanaka, K.I.; Notsu, M.; Tanaka, S.; Kiyohara, N.; Koike, S.; Yamane, Y.; Tada, Y.; Sasaki, M.; Yamauchi, M.; et al. Long-term efficacy and safety of vildagliptin add-on therapy in type 2 diabetes mellitus with insulin treatment. *Diabetes Res Clin Pract* **2017**, *123*, 9-17, doi:10.1016/j.diabres.2016.11.010.
254. Katsuno, T.; Shiraiwa, T.; Iwasaki, S.; Park, H.; Watanabe, N.; Kaneko, S.; Terasaki, J.; Hanafusa, T.; Imagawa, A.; Shimomura, I.; et al. Benefit of Early Add-on of Linagliptin to Insulin in Japanese Patients With Type 2 Diabetes Mellitus: Randomized-Controlled Open-Label Trial (TRUST2). *Adv Ther* **2021**, *38*, 1514-1535, doi:10.1007/s12325-021-01631-y.
255. Kikuchi, M.; Abe, N.; Kato, M.; Terao, S.; Mimori, N.; Tachibana, H. Vildagliptin dose-dependently improves glycemic control in Japanese patients with type 2 diabetes mellitus. *Diabetes Res Clin Pract* **2009**, *83*, 233-240, doi:10.1016/j.diabres.2008.10.006.
256. Kikuchi, M.; Haneda, M.; Koya, D.; Tobe, K.; Onishi, Y.; Couturier, A.; Mimori, N.; Inaba, Y.; Goodman, M. Efficacy and tolerability of vildagliptin as an add-on to glimepiride in Japanese patients with Type 2 diabetes mellitus. *Diabetes Res Clin Pract* **2010**, *89*, 216-223, doi:10.1016/j.diabres.2010.04.017.
257. Kim, M.K.; Rhee, E.J.; Han, K.A.; Woo, A.C.; Lee, M.K.; Ku, B.J.; Chung, C.H.; Kim, K.A.; Lee, H.W.; Park, I.B.; et al. Efficacy and safety of teneligliptin, a dipeptidyl peptidase-4 inhibitor, combined with metformin in Korean patients with type 2 diabetes mellitus: a 16-week, randomized, double-blind, placebo-controlled phase III trial. *Diabetes Obes Metab* **2015**, *17*, 309-312, doi:10.1111/dom.12424.
258. Kothny, W.; Shao, Q.; Groop, P.H.; Lukashevich, V. One-year safety, tolerability and efficacy of vildagliptin in patients with type 2 diabetes and moderate or severe renal impairment. *Diabetes Obes Metab* **2012**, *14*, 1032-1039, doi:10.1111/j.1463-1326.2012.01634.x.
259. Kothny, W.; Foley, J.; Kozlovski, P.; Shao, Q.; Gallwitz, B.; Lukashevich, V. Improved glycaemic control with vildagliptin added to insulin, with or without metformin, in patients with type 2 diabetes mellitus. *Diabetes Obes Metab* **2013**, *15*, 252-257, doi:10.1111/dom.12020.
260. Lee, M.M.Y.; Brooksbank, K.J.M.; Wetherall, K.; Mangion, K.; Roditi, G.; Campbell, R.T.; Berry, C.; Chong, V.; Coyle, L.; Docherty, K.F.; et al.

Effect of Empagliflozin on Left Ventricular Volumes in Patients With Type 2 Diabetes, or Prediabetes, and Heart Failure With Reduced Ejection Fraction (SUGAR-DM-HF). *Circulation* **2021**, *143*, 516-525, doi:10.1161/CIRCULATIONAHA.120.052186.

261. Lukashevich, V.; Del Prato, S.; Araga, M.; Kothny, W. Efficacy and safety of vildagliptin in patients with type 2 diabetes mellitus inadequately controlled with dual combination of metformin and sulphonylurea. *Diabetes Obes Metab* **2014**, *16*, 403-409, doi:10.1111/dom.12229.

262. Macauley, M.; Hollingsworth, K.G.; Smith, F.E.; Thelwall, P.E.; Al-Mrabeh, A.; Schweizer, A.; Foley, J.E.; Taylor, R. Effect of vildagliptin on hepatic steatosis. *J Clin Endocrinol Metab* **2015**, *100*, 1578-1585, doi:10.1210/jc.2014-3794.

263. McGill, J.B.; Sloan, L.; Newman, J.; Patel, S.; Sauce, C.; von Eynatten, M.; Woerle, H.J. Long-term efficacy and safety of linagliptin in patients with type 2 diabetes and severe renal impairment: a 1-year, randomized, double-blind, placebo-controlled study. *Diabetes Care* **2013**, *36*, 237-244, doi:10.2337/dc12-0706.

264. McMurray, J.J.V.; Ponikowski, P.; Bolli, G.B.; Lukashevich, V.; Kozlovski, P.; Kothny, W.; Lewsey, J.D.; Krum, H.; Committees, V.T.; Investigators. Effects of Vildagliptin on Ventricular Function in Patients With Type 2 Diabetes Mellitus and Heart Failure: A Randomized Placebo-Controlled Trial. *JACC Heart Fail* **2018**, *6*, 8-17, doi:10.1016/j.jchf.2017.08.004.

265. Mita, T.; Katakami, N.; Yoshii, H.; Onuma, T.; Kaneto, H.; Osonoi, T.; Shiraiwa, T.; Kosugi, K.; Umayahara, Y.; Yamamoto, T.; et al. Alogliptin, a Dipeptidyl Peptidase 4 Inhibitor, Prevents the Progression of Carotid Atherosclerosis in Patients With Type 2 Diabetes: The Study of Preventive Effects of Alogliptin on Diabetic Atherosclerosis (SPEAD-A). *Diabetes Care* **2016**, *39*, 139-148, doi:10.2337/dc15-0781.

266. Mita, T.; Katakami, N.; Shiraiwa, T.; Yoshii, H.; Onuma, T.; Kuribayashi, N.; Osonoi, T.; Kaneto, H.; Kosugi, K.; Umayahara, Y.; et al. Sitagliptin Attenuates the Progression of Carotid Intima-Media Thickening in Insulin-Treated Patients With Type 2 Diabetes: The Sitagliptin Preventive Study of Intima-Media Thickness Evaluation (SPIKE): A Randomized Controlled Trial. *Diabetes Care* **2016**, *39*, 455-464, doi:10.2337/dc15-2145.

267. Neves, J.S.; Vasques-Novoa, F.; Borges-Canha, M.; Leite, A.R.; Sharma, A.; Carvalho, D.; Packer, M.; Zannad, F.; Leite-Moreira, A.; Ferreira, J.P. Risk of adverse events with liraglutide in heart failure with reduced ejection fraction: A post hoc analysis of the FIGHT trial. *Diabetes Obes Metab* **2023**, *25*, 189-197, doi:10.1111/dom.14862.

268. Nielsen, R.; Jorsal, A.; Tougaard, R.S.; Rasmussen, J.J.; Schou, M.; Videbaek, L.; Gustafsson, I.; Faber, J.; Flyvbjerg, A.; Wiggers, H.; et al. The impact of the glucagon-like peptide-1 receptor agonist liraglutide on natriuretic peptides in heart failure patients with reduced ejection fraction with and without type 2 diabetes. *Diabetes Obes Metab* **2020**, *22*, 2141-2150, doi:10.1111/dom.14135.
269. Ning, G.; Wang, W.; Li, L.; Ma, J.; Lv, X.; Yang, M.; Wang, W.; Woloschak, M.; Lukashevich, V.; Kothny, W. Vildagliptin as add-on therapy to insulin improves glycemic control without increasing risk of hypoglycemia in Asian, predominantly Chinese, patients with type 2 diabetes mellitus. *J Diabetes* **2016**, *8*, 345-353, doi:10.1111/1753-0407.12303.
270. Nomoto, H.; Takahashi, A.; Nakamura, A.; Kurihara, H.; Takeuchi, J.; Nagai, S.; Taneda, S.; Miya, A.; Kameda, H.; Cho, K.Y.; et al. Add-on imeglimin versus metformin dose escalation regarding glycemic control in patients with type 2 diabetes treated with a dipeptidyl peptidase-4 inhibitor plus low-dose metformin: study protocol for a multicenter, prospective, randomized, open-label, parallel-group comparison study (MEGMI study). *BMJ Open Diabetes Res Care* **2022**, *10*, doi:10.1136/bmjdrc-2022-002988.
271. Nordisk, N. A Research Study to Look Into How Well Semaglutide Medicine Works at Different Doses in People With Type 2 Diabetes and Overweight. Available online: https://clinicaltrials.gov/study/NCT05486065?cond=NCT05486065&rank=1 (accessed on 2025/05/20).
272. Odawara, M.; Hamada, I.; Suzuki, M. Efficacy and Safety of Vildagliptin as Add-on to Metformin in Japanese Patients with Type 2 Diabetes Mellitus. *Diabetes Ther* **2014**, *5*, 169-181, doi:10.1007/s13300-014-0059-x.
273. Olansky, L.; Reasner, C.; Seck, T.L.; Williams-Herman, D.E.; Chen, M.; Terranella, L.; Mehta, A.; Kaufman, K.D.; Goldstein, B.J. A treatment strategy implementing combination therapy with sitagliptin and metformin results in superior glycaemic control versus metformin monotherapy due to a low rate of addition of antihyperglycaemic agents. *Diabetes Obes Metab* **2011**, *13*, 841-849, doi:10.1111/j.1463-1326.2011.01416.x.
274. Oyanagi, T.; Kawanabe, S.; Tsukiyama, H.; Nishine, A.; Nakamura, Y.; Nakagawa, T.; Kanou, M.; Kubota, J.; Tsunemi, S.; Yokota, K.; et al. The Effects of Imeglimin on Muscle Strength in Patients with Type 2 Diabetes: A Prospective Cohort Study. *Diabetes Ther* **2024**, *15*, 2323-2336, doi:10.1007/s13300-024-01639-x.
275. Pacini, G.; Mari, A.; Fouqueray, P.; Bolze, S.; Roden, M. Imeglimin increases glucose-dependent insulin secretion and improves beta-cell function in patients with type 2 diabetes. *Diabetes Obes Metab* **2015**, *17*, 541-545, doi:10.1111/dom.12452.

276. Palau, P.; Amiguet, M.; Dominguez, E.; Sastre, C.; Mollar, A.; Seller, J.; Garcia Pinilla, J.M.; Larumbe, A.; Valle, A.; Gomez Doblas, J.J.; et al. Short-term effects of dapagliflozin on maximal functional capacity in heart failure with reduced ejection fraction (DAPA-VO(2)): a randomized clinical trial. *Eur J Heart Fail* **2022**, *24*, 1816-1826, doi:10.1002/ejhf.2560.
277. Peng, X.V.; Marcinak, J.F.; Raanan, M.G.; Cao, C. Combining the G-protein-coupled receptor 40 agonist fasiglifam with sitagliptin improves glycaemic control in patients with type 2 diabetes with or without metformin: A randomized, 12-week trial. *Diabetes Obes Metab* **2017**, *19*, 1127-1134, doi:10.1111/dom.12921.
278. Pirags, V.; Lebovitz, H.; Fouqueray, P. Imeglimin, a novel glimin oral antidiabetic, exhibits a good efficacy and safety profile in type 2 diabetic patients. *Diabetes Obes Metab* **2012**, *14*, 852-858, doi:10.1111/j.1463-1326.2012.01611.x.
279. Raz, I.; Hanefeld, M.; Xu, L.; Caria, C.; Williams-Herman, D.; Khatami, H.; Sitagliptin Study, G. Efficacy and safety of the dipeptidyl peptidase-4 inhibitor sitagliptin as monotherapy in patients with type 2 diabetes mellitus. *Diabetologia* **2006**, *49*, 2564-2571, doi:10.1007/s00125-006-0416-z.
280. Reilhac, C.; Dubourg, J.; Thang, C.; Grouin, J.M.; Fouqueray, P.; Watada, H. Efficacy and safety of imeglimin add-on to insulin monotherapy in Japanese patients with type 2 diabetes (TIMES 3): A randomized, double-blind, placebo-controlled phase 3 trial with a 36-week open-label extension period. *Diabetes Obes Metab* **2022**, *24*, 838-848, doi:10.1111/dom.14642.
281. Rosenstock, J.; Foley, J.E.; Rendell, M.; Landin-Olsson, M.; Holst, J.J.; Deacon, C.F.; Rochotte, E.; Baron, M.A. Effects of the dipeptidyl peptidase-IV inhibitor vildagliptin on incretin hormones, islet function, and postprandial glycemia in subjects with impaired glucose tolerance. *Diabetes Care* **2008**, *31*, 30-35, doi:10.2337/dc07-1616.
282. Rosenstock, J.; Sankoh, S.; List, J.F. Glucose-lowering activity of the dipeptidyl peptidase-4 inhibitor saxagliptin in drug-naive patients with type 2 diabetes. *Diabetes Obes Metab* **2008**, *10*, 376-386, doi:10.1111/j.1463-1326.2008.00876.x.
283. Saito, D.; Kanazawa, A.; Shigihara, N.; Sato, F.; Uchida, T.; Sato, J.; Goto, H.; Miyatsuka, T.; Ikeda, F.; Ogihara, T.; et al. Efficacy and Safety of Vildagliptin as an Add-On Therapy in Inadequately Controlled Type 2 Diabetes Patients Treated With Basal Insulin. *J Clin Med Res* **2017**, *9*, 193-199, doi:10.14740/jocmr2874w.
284. Sanyal, A.J.; Kaplan, L.M.; Frias, J.P.; Brouwers, B.; Wu, Q.; Thomas, M.K.; Harris, C.; Schloot, N.C.; Du, Y.; Mather, K.J.; et al. Triple

hormone receptor agonist retatrutide for metabolic dysfunction-associated steatotic liver disease: a randomized phase 2a trial. *Nat Med* **2024**, *30*, 2037-2048, doi:10.1038/s41591-024-03018-2.
285. Scherbaum, W.A.; Schweizer, A.; Mari, A.; Nilsson, P.M.; Lalanne, G.; Jauffret, S.; Foley, J.E. Efficacy and tolerability of vildagliptin in drug-naive patients with type 2 diabetes and mild hyperglycaemia*. *Diabetes Obes Metab* **2008**, *10*, 675-682, doi:10.1111/j.1463-1326.2008.00850.x.
286. Strain, W.D.; Lukashevich, V.; Kothny, W.; Hoellinger, M.J.; Paldanius, P.M. Individualised treatment targets for elderly patients with type 2 diabetes using vildagliptin add-on or lone therapy (INTERVAL): a 24 week, randomised, double-blind, placebo-controlled study. *Lancet* **2013**, *382*, 409-416, doi:10.1016/S0140-6736(13)60995-2.
287. Takahashi, A.; Nomoto, H.; Onishi, K.; Manda, S.; Miya, A.; Kameda, H.; Nakamura, A.; Atsumi, T. A comparative study of the effects of imeglimin add-on or metformin dose escalation on glycaemic variability in subjects with type 2 diabetes treated with low-dose metformin (MEGMI-CGM study). *Diabetes Obes Metab* **2024**, *26*, 3471-3474, doi:10.1111/dom.15639.
288. Takahashi, A.; Nomoto, H.; Yokoyama, H.; Yokozeki, K.; Furusawa, S.; Oe, Y.; Kameda, R.; Kawata, S.; Miyoshi, A.; Nagai, S.; et al. Efficacy of imeglimin treatment versus metformin dose escalation on glycemic control in subjects with type 2 diabetes treated with a dipeptidyl peptidase-4 inhibitor plus low-dose metformin: A multicenter, prospective, randomized, open-label, parallel-group comparison study (MEGMI study). *Diabetes Obes Metab* **2025**, *27*, 1466-1476, doi:10.1111/dom.16150.
289. Theurey, P.; Thang, C.; Pirags, V.; Mari, A.; Pacini, G.; Bolze, S.; Hallakou-Bozec, S.; Fouqueray, P. Phase 2 trial with imeglimin in patients with Type 2 diabetes indicates effects on insulin secretion and sensitivity. *Endocrinol Diabetes Metab* **2022**, *5*, e371, doi:10.1002/edm2.371.
290. Usui, R.; Hamamoto, Y.; Imura, M.; Omori, Y.; Yamazaki, Y.; Kuwata, H.; Tatsuoka, H.; Shimomura, K.; Murotani, K.; Yamada, Y.; et al. Differential effects of imeglimin and metformin on insulin and incretin secretion-An exploratory randomized controlled trial. *Diabetes Obes Metab* **2025**, *27*, 856-865, doi:10.1111/dom.16086.
291. Yang, W.; Xing, X.; Lv, X.; Li, Y.; Ma, J.; Yuan, G.; Sun, F.; Wang, W.; Woloschak, M.; Lukashevich, V.; et al. Vildagliptin added to sulfonylurea improves glycemic control without hypoglycemia and weight gain in Chinese patients with type 2 diabetes mellitus. *J*

Diabetes **2015**, *7*, 174-181, doi:10.1111/1753-0407.12169.
292. Yang, W.; Zhu, D.; Gan, S.; Dong, X.; Su, J.; Li, W.; Jiang, H.; Zhao, W.; Yao, M.; Song, W.; et al. Dorzagliatin add-on therapy to metformin in patients with type 2 diabetes: a randomized, double-blind, placebo-controlled phase 3 trial. *Nat Med* **2022**, *28*, 974-981, doi:10.1038/s41591-022-01803-5.
293. Younis, A.; Eskenazi, D.; Goldkorn, R.; Leor, J.; Naftali-Shani, N.; Fisman, E.Z.; Tenenbaum, A.; Goldenberg, I.; Klempfner, R. The addition of vildagliptin to metformin prevents the elevation of interleukin 1ss in patients with type 2 diabetes and coronary artery disease: a prospective, randomized, open-label study. *Cardiovasc Diabetol* **2017**, *16*, 69, doi:10.1186/s12933-017-0551-5.
294. Zhu, D.; Li, X.; Ma, J.; Zeng, J.; Gan, S.; Dong, X.; Yang, J.; Lin, X.; Cai, H.; Song, W.; et al. Dorzagliatin in drug-naive patients with type 2 diabetes: a randomized, double-blind, placebo-controlled phase 3 trial. *Nat Med* **2022**, *28*, 965-973, doi:10.1038/s41591-022-01802-6.
295. Zhu, X.X.; Zhu, D.L.; Li, X.Y.; Li, Y.L.; Jin, X.W.; Hu, T.X.; Zhao, Y.; Li, Y.G.; Zhao, G.Y.; Ren, S.; et al. Dorzagliatin (HMS5552), a novel dual-acting glucokinase activator, improves glycaemic control and pancreatic beta-cell function in patients with type 2 diabetes: A 28-day treatment study using biomarker-guided patient selection. *Diabetes Obes Metab* **2018**, *20*, 2113-2120, doi:10.1111/dom.13338.
296. McGuire, D.K.; Marx, N.; Mulvagh, S.L.; Deanfield, J.E.; Inzucchi, S.E.; Pop-Busui, R.; Mann, J.F.E.; Emerson, S.S.; Poulter, N.R.; Engelmann, M.D.M.; et al. Oral Semaglutide and Cardiovascular Outcomes in High-Risk Type 2 Diabetes. *N Engl J Med* **2025**, *392*, 2001-2012, doi:10.1056/NEJMoa2501006.
297. Perkovic, V.; Tuttle, K.R.; Rossing, P.; Mahaffey, K.W.; Mann, J.F.E.; Bakris, G.; Baeres, F.M.M.; Idorn, T.; Bosch-Traberg, H.; Lausvig, N.L.; et al. Effects of Semaglutide on Chronic Kidney Disease in Patients with Type 2 Diabetes. *N Engl J Med* **2024**, *391*, 109-121, doi:10.1056/NEJMoa2403347.
298. Lincoff, A.M.; Brown-Frandsen, K.; Colhoun, H.M.; Deanfield, J.; Emerson, S.S.; Esbjerg, S.; Hardt-Lindberg, S.; Hovingh, G.K.; Kahn, S.E.; Kushner, R.F.; et al. Semaglutide and Cardiovascular Outcomes in Obesity without Diabetes. *N Engl J Med* **2023**, *389*, 2221-2232, doi:10.1056/NEJMoa2307563.
299. Kellerer, M.; Kaltoft, M.S.; Lawson, J.; Nielsen, L.L.; Strojek, K.; Tabak, O.; Jacob, S. Effect of once-weekly semaglutide versus thrice-daily insulin aspart, both as add-on to metformin and optimized insulin glargine treatment in participants with type 2 diabetes (SUSTAIN 11):

A randomized, open-label, multinational, phase 3b trial. *Diabetes Obes Metab* **2022**, *24*, 1788-1799, doi:10.1111/dom.14765.

300. Bhatt, D.L.; Szarek, M.; Steg, P.G.; Cannon, C.P.; Leiter, L.A.; McGuire, D.K.; Lewis, J.B.; Riddle, M.C.; Voors, A.A.; Metra, M.; et al. Sotagliflozin in Patients with Diabetes and Recent Worsening Heart Failure. *N Engl J Med* **2021**, *384*, 117-128, doi:10.1056/NEJMoa2030183.
301. Wilding, J.P.H.; Batterham, R.L.; Calanna, S.; Davies, M.; Van Gaal, L.F.; Lingvay, I.; McGowan, B.M.; Rosenstock, J.; Tran, M.T.D.; Wadden, T.A.; et al. Once-Weekly Semaglutide in Adults with Overweight or Obesity. *N Engl J Med* **2021**, *384*, 989-1002, doi:10.1056/NEJMoa2032183.
302. Gerstein, H.C.; Colhoun, H.M.; Dagenais, G.R.; Diaz, R.; Lakshmanan, M.; Pais, P.; Probstfield, J.; Riesmeyer, J.S.; Riddle, M.C.; Ryden, L.; et al. Dulaglutide and cardiovascular outcomes in type 2 diabetes (REWIND): a double-blind, randomised placebo-controlled trial. *Lancet* **2019**, *394*, 121-130, doi:10.1016/S0140-6736(19)31149-3.
303. Pieber, T.R.; Bode, B.; Mertens, A.; Cho, Y.M.; Christiansen, E.; Hertz, C.L.; Wallenstein, S.O.R.; Buse, J.B.; investigators, P. Efficacy and safety of oral semaglutide with flexible dose adjustment versus sitagliptin in type 2 diabetes (PIONEER 7): a multicentre, open-label, randomised, phase 3a trial. *Lancet Diabetes Endocrinol* **2019**, *7*, 528-539, doi:10.1016/S2213-8587(19)30194-9.
304. Rosenstock, J.; Perkovic, V.; Johansen, O.E.; Cooper, M.E.; Kahn, S.E.; Marx, N.; Alexander, J.H.; Pencina, M.; Toto, R.D.; Wanner, C.; et al. Effect of Linagliptin vs Placebo on Major Cardiovascular Events in Adults With Type 2 Diabetes and High Cardiovascular and Renal Risk: The CARMELINA Randomized Clinical Trial. *Jama* **2019**, *321*, 69-79, doi:10.1001/jama.2018.18269.
305. Zinman, B.; Aroda, V.R.; Buse, J.B.; Cariou, B.; Harris, S.B.; Hoff, S.T.; Pedersen, K.B.; Tarp-Johansen, M.J.; Araki, E.; Investigators, P. Efficacy, Safety, and Tolerability of Oral Semaglutide Versus Placebo Added to Insulin With or Without Metformin in Patients With Type 2 Diabetes: The PIONEER 8 Trial. *Diabetes Care* **2019**, *42*, 2262-2271, doi:10.2337/dc19-0898.
306. Hernandez, A.F.; Green, J.B.; Janmohamed, S.; D'Agostino, R.B., Sr.; Granger, C.B.; Jones, N.P.; Leiter, L.A.; Rosenberg, A.E.; Sigmon, K.N.; Somerville, M.C.; et al. Albiglutide and cardiovascular outcomes in patients with type 2 diabetes and cardiovascular disease (Harmony Outcomes): a double-blind, randomised placebo-controlled trial. *Lancet* **2018**, *392*, 1519-1529, doi:10.1016/S0140-6736(18)32261-X.
307. Davies, M.; Pieber, T.R.; Hartoft-Nielsen, M.L.; Hansen, O.K.H.; Jabbour, S.; Rosenstock, J. Effect of Oral Semaglutide Compared With

Placebo and Subcutaneous Semaglutide on Glycemic Control in Patients With Type 2 Diabetes: A Randomized Clinical Trial. *Jama* **2017**, *318*, 1460-1470, doi:10.1001/jama.2017.14752.

308. Gantz, I.; Chen, M.; Suryawanshi, S.; Ntabadde, C.; Shah, S.; O'Neill, E.A.; Engel, S.S.; Kaufman, K.D.; Lai, E. A randomized, placebo-controlled study of the cardiovascular safety of the once-weekly DPP-4 inhibitor omarigliptin in patients with type 2 diabetes mellitus. *Cardiovasc Diabetol* **2017**, *16*, 112, doi:10.1186/s12933-017-0593-8.

309. Marso, S.P.; Bain, S.C.; Consoli, A.; Eliaschewitz, F.G.; Jodar, E.; Leiter, L.A.; Lingvay, I.; Rosenstock, J.; Seufert, J.; Warren, M.L.; et al. Semaglutide and Cardiovascular Outcomes in Patients with Type 2 Diabetes. *N Engl J Med* **2016**, *375*, 1834-1844, doi:10.1056/NEJMoa1607141.

310. Pfeffer, M.A.; Claggett, B.; Diaz, R.; Dickstein, K.; Gerstein, H.C.; Kober, L.V.; Lawson, F.C.; Ping, L.; Wei, X.; Lewis, E.F.; et al. Lixisenatide in Patients with Type 2 Diabetes and Acute Coronary Syndrome. *N Engl J Med* **2015**, *373*, 2247-2257, doi:10.1056/NEJMoa1509225.

311. Pi-Sunyer, X.; Astrup, A.; Fujioka, K.; Greenway, F.; Halpern, A.; Krempf, M.; Lau, D.C.; le Roux, C.W.; Violante Ortiz, R.; Jensen, C.B.; et al. A Randomized, Controlled Trial of 3.0 mg of Liraglutide in Weight Management. *N Engl J Med* **2015**, *373*, 11-22, doi:10.1056/NEJMoa1411892.

312. Weinstock, R.S.; Guerci, B.; Umpierrez, G.; Nauck, M.A.; Skrivanek, Z.; Milicevic, Z. Safety and efficacy of once-weekly dulaglutide versus sitagliptin after 2 years in metformin-treated patients with type 2 diabetes (AWARD-5): a randomized, phase III study. *Diabetes Obes Metab* **2015**, *17*, 849-858, doi:10.1111/dom.12479.

313. Pratley, R.E.; Nauck, M.A.; Barnett, A.H.; Feinglos, M.N.; Ovalle, F.; Harman-Boehm, I.; Ye, J.; Scott, R.; Johnson, S.; Stewart, M.; et al. Once-weekly albiglutide versus once-daily liraglutide in patients with type 2 diabetes inadequately controlled on oral drugs (HARMONY 7): a randomised, open-label, multicentre, non-inferiority phase 3 study. *Lancet Diabetes Endocrinol* **2014**, *2*, 289-297, doi:10.1016/S2213-8587(13)70214-6.

314. Arjona Ferreira, J.C.; Marre, M.; Barzilai, N.; Guo, H.; Golm, G.T.; Sisk, C.M.; Kaufman, K.D.; Goldstein, B.J. Efficacy and safety of sitagliptin versus glipizide in patients with type 2 diabetes and moderate-to-severe chronic renal insufficiency. *Diabetes Care* **2013**, *36*, 1067-1073, doi:10.2337/dc12-1365.

315. Kadowaki, T.; Kondo, K. Efficacy and safety of teneligliptin in combination with pioglitazone in Japanese patients with type 2 diabetes mellitus. *J Diabetes Investig* **2013**, *4*, 576-584, doi:10.1111/jdi.12092.
316. Scirica, B.M.; Bhatt, D.L.; Braunwald, E.; Steg, P.G.; Davidson, J.; Hirshberg, B.; Ohman, P.; Frederich, R.; Wiviott, S.D.; Hoffman, E.B.; et al. Saxagliptin and cardiovascular outcomes in patients with type 2 diabetes mellitus. *N Engl J Med* **2013**, *369*, 1317-1326, doi:10.1056/NEJMoa1307684.
317. Gallwitz, B.; Rosenstock, J.; Rauch, T.; Bhattacharya, S.; Patel, S.; von Eynatten, M.; Dugi, K.A.; Woerle, H.J. 2-year efficacy and safety of linagliptin compared with glimepiride in patients with type 2 diabetes inadequately controlled on metformin: a randomised, double-blind, non-inferiority trial. *Lancet* **2012**, *380*, 475-483, doi:10.1016/S0140-6736(12)60691-6.
318. Pratley, R.E.; Nauck, M.; Bailey, T.; Montanya, E.; Cuddihy, R.; Filetti, S.; Thomsen, A.B.; Sondergaard, R.E.; Davies, M.; Group, L.-D.-S. Liraglutide versus sitagliptin for patients with type 2 diabetes who did not have adequate glycaemic control with metformin: a 26-week, randomised, parallel-group, open-label trial. *Lancet* **2010**, *375*, 1447-1456, doi:10.1016/S0140-6736(10)60307-8.
319. Natale, P.; Tunnicliffe, D.J.; Toyama, T.; Palmer, S.C.; Saglimbene, V.M.; Ruospo, M.; Gargano, L.; Stallone, G.; Gesualdo, L.; Strippoli, G.F. Sodium-glucose co-transporter protein 2 (SGLT2) inhibitors for people with chronic kidney disease and diabetes. *The Cochrane database of systematic reviews* **2024**, *5*, CD015588, doi:10.1002/14651858.CD015588.pub2.
320. SURMOUNT-J. A Study of Tirzepatide (LY3298176) in Participants With Obesity Disease (SURMOUNT-J). Available online: https://clinicaltrials.gov/study/NCT04844918?cond=NCT04844918&rank=1 (accessed on 2024/10/28).
321. Buse, J.B.; Nordahl Christensen, H.; Harty, B.J.; Mitchell, J.; Soule, B.P.; Zacherle, E.; Cziraky, M.; Willey, V.J. Study design and baseline profile for adults with type 2 diabetes in the once-weekly subcutaneous SEmaglutide randomized PRAgmatic (SEPRA) trial. *BMJ Open Diabetes Res Care* **2023**, *11*, doi:10.1136/bmjdrc-2022-003206.
322. Garvey, W.T.; Frias, J.P.; Jastreboff, A.M.; le Roux, C.W.; Sattar, N.; Aizenberg, D.; Mao, H.; Zhang, S.; Ahmad, N.N.; Bunck, M.C.; et al. Tirzepatide once weekly for the treatment of obesity in people with type 2 diabetes (SURMOUNT-2): a double-blind, randomised, multicentre, placebo-controlled, phase 3 trial. *Lancet* **2023**, *402*, 613-626, doi:10.1016/S0140-6736(23)01200-X.
323. The, E.-K.C.G.; Herrington, W.G.; Staplin, N.; Wanner, C.; Green, J.B.; Hauske, S.J.; Emberson, J.R.; Preiss, D.; Judge, P.; Mayne, K.J.; et al.

Empagliflozin in Patients with Chronic Kidney Disease. *N Engl J Med* **2023**, *388*, 117-127, doi:10.1056/NEJMoa2204233.
324. Solomon, S.D.; McMurray, J.J.V.; Claggett, B.; de Boer, R.A.; DeMets, D.; Hernandez, A.F.; Inzucchi, S.E.; Kosiborod, M.N.; Lam, C.S.P.; Martinez, F.; et al. Dapagliflozin in Heart Failure with Mildly Reduced or Preserved Ejection Fraction. *N Engl J Med* **2022**, *387*, 1089-1098, doi:10.1056/NEJMoa2206286.
325. Tuttle, K.R.; Levin, A.; Nangaku, M.; Kadowaki, T.; Agarwal, R.; Hauske, S.J.; Elsasser, A.; Ritter, I.; Steubl, D.; Wanner, C.; et al. Safety of Empagliflozin in Patients With Type 2 Diabetes and Chronic Kidney Disease: Pooled Analysis of Placebo-Controlled Clinical Trials. *Diabetes Care* **2022**, *45*, 1445-1452, doi:10.2337/dc21-2034.
326. Wada, T.; Mori-Anai, K.; Takahashi, A.; Matsui, T.; Inagaki, M.; Iida, M.; Maruyama, K.; Tsuda, H. Effect of canagliflozin on the decline of estimated glomerular filtration rate in chronic kidney disease patients with type 2 diabetes mellitus: A multicenter, randomized, double-blind, placebo-controlled, parallel-group, phase III study in Japan. *J Diabetes Investig* **2022**, *13*, 1981-1989, doi:10.1111/jdi.13888.
327. Anker, S.D.; Butler, J.; Filippatos, G.; Ferreira, J.P.; Bocchi, E.; Bohm, M.; Brunner-La Rocca, H.P.; Choi, D.J.; Chopra, V.; Chuquiure-Valenzuela, E.; et al. Empagliflozin in Heart Failure with a Preserved Ejection Fraction. *N Engl J Med* **2021**, *385*, 1451-1461, doi:10.1056/NEJMoa2107038.
328. Bhatt, D.L.; Szarek, M.; Pitt, B.; Cannon, C.P.; Leiter, L.A.; McGuire, D.K.; Lewis, J.B.; Riddle, M.C.; Inzucchi, S.E.; Kosiborod, M.N.; et al. Sotagliflozin in Patients with Diabetes and Chronic Kidney Disease. *N Engl J Med* **2021**, *384*, 129-139, doi:10.1056/NEJMoa2030186.
329. Davies, M.; Faerch, L.; Jeppesen, O.K.; Pakseresht, A.; Pedersen, S.D.; Perreault, L.; Rosenstock, J.; Shimomura, I.; Viljoen, A.; Wadden, T.A.; et al. Semaglutide 2.4 mg once a week in adults with overweight or obesity, and type 2 diabetes (STEP 2): a randomised, double-blind, double-dummy, placebo-controlled, phase 3 trial. *Lancet* **2021**, *397*, 971-984, doi:10.1016/S0140-6736(21)00213-0.
330. Lock, J.P. Bexagliflozin Efficacy and Safety Trial (BEST). Available online: https://clinicaltrials.gov/study/NCT02558296?cond=NCT02558296&rank=1 (accessed on 2024/10/28).
331. Ludvik, B.; Giorgino, F.; Jodar, E.; Frias, J.P.; Fernandez Lando, L.; Brown, K.; Bray, R.; Rodriguez, A. Once-weekly tirzepatide versus once-daily insulin degludec as add-on to metformin with or without SGLT2 inhibitors in patients with type 2 diabetes (SURPASS-3): a randomised, open-label, parallel-group, phase 3 trial. *Lancet* **2021**, *398*, 583-598, doi:10.1016/S0140-6736(21)01443-4.

332. Cannon, C.P.; Pratley, R.; Dagogo-Jack, S.; Mancuso, J.; Huyck, S.; Masiukiewicz, U.; Charbonnel, B.; Frederich, R.; Gallo, S.; Cosentino, F.; et al. Cardiovascular Outcomes with Ertugliflozin in Type 2 Diabetes. *N Engl J Med* **2020**, *383*, 1425-1435, doi:10.1056/NEJMoa2004967.
333. Heerspink, H.J.L.; Stefansson, B.V.; Correa-Rotter, R.; Chertow, G.M.; Greene, T.; Hou, F.F.; Mann, J.F.E.; McMurray, J.J.V.; Lindberg, M.; Rossing, P.; et al. Dapagliflozin in Patients with Chronic Kidney Disease. *N Engl J Med* **2020**, *383*, 1436-1446, doi:10.1056/NEJMoa2024816.
334. Packer, M.; Anker, S.D.; Butler, J.; Filippatos, G.; Pocock, S.J.; Carson, P.; Januzzi, J.; Verma, S.; Tsutsui, H.; Brueckmann, M.; et al. Cardiovascular and Renal Outcomes with Empagliflozin in Heart Failure. *N Engl J Med* **2020**, *383*, 1413-1424, doi:10.1056/NEJMoa2022190.
335. Husain, M.; Birkenfeld, A.L.; Donsmark, M.; Dungan, K.; Eliaschewitz, F.G.; Franco, D.R.; Jeppesen, O.K.; Lingvay, I.; Mosenzon, O.; Pedersen, S.D.; et al. Oral Semaglutide and Cardiovascular Outcomes in Patients with Type 2 Diabetes. *N Engl J Med* **2019**, *381*, 841-851, doi:10.1056/NEJMoa1901118.
336. Ledesma, G.; Umpierrez, G.E.; Morley, J.E.; Lewis-D'Agostino, D.; Keller, A.; Meinicke, T.; van der Walt, S.; von Eynatten, M. Efficacy and safety of linagliptin to improve glucose control in older people with type 2 diabetes on stable insulin therapy: A randomized trial. *Diabetes Obes Metab* **2019**, *21*, 2465-2473, doi:10.1111/dom.13829.
337. Matthews, D.R.; Paldanius, P.M.; Proot, P.; Chiang, Y.; Stumvoll, M.; Del Prato, S.; group, V.s. Glycaemic durability of an early combination therapy with vildagliptin and metformin versus sequential metformin monotherapy in newly diagnosed type 2 diabetes (VERIFY): a 5-year, multicentre, randomised, double-blind trial. *Lancet* **2019**, *394*, 1519-1529, doi:10.1016/S0140-6736(19)32131-2.
338. McMurray, J.J.V.; Solomon, S.D.; Inzucchi, S.E.; Kober, L.; Kosiborod, M.N.; Martinez, F.A.; Ponikowski, P.; Sabatine, M.S.; Anand, I.S.; Belohlavek, J.; et al. Dapagliflozin in Patients with Heart Failure and Reduced Ejection Fraction. *N Engl J Med* **2019**, *381*, 1995-2008, doi:10.1056/NEJMoa1911303.
339. Perkovic, V.; Jardine, M.J.; Neal, B.; Bompoint, S.; Heerspink, H.J.L.; Charytan, D.M.; Edwards, R.; Agarwal, R.; Bakris, G.; Bull, S.; et al. Canagliflozin and Renal Outcomes in Type 2 Diabetes and Nephropathy. *N Engl J Med* **2019**, *380*, 2295-2306, doi:10.1056/NEJMoa1811744.

340. Rosenstock, J.; Kahn, S.E.; Johansen, O.E.; Zinman, B.; Espeland, M.A.; Woerle, H.J.; Pfarr, E.; Keller, A.; Mattheus, M.; Baanstra, D.; et al. Effect of Linagliptin vs Glimepiride on Major Adverse Cardiovascular Outcomes in Patients With Type 2 Diabetes: The CAROLINA Randomized Clinical Trial. *Jama* **2019**, *322*, 1155-1166, doi:10.1001/jama.2019.13772.
341. Rosenstock, J.; Allison, D.; Birkenfeld, A.L.; Blicher, T.M.; Deenadayalan, S.; Jacobsen, J.B.; Serusclat, P.; Violante, R.; Watada, H.; Davies, M.; et al. Effect of Additional Oral Semaglutide vs Sitagliptin on Glycated Hemoglobin in Adults With Type 2 Diabetes Uncontrolled With Metformin Alone or With Sulfonylurea: The PIONEER 3 Randomized Clinical Trial. *Jama* **2019**, *321*, 1466-1480, doi:10.1001/jama.2019.2942.
342. Wiviott, S.D.; Raz, I.; Bonaca, M.P.; Mosenzon, O.; Kato, E.T.; Cahn, A.; Silverman, M.G.; Zelniker, T.A.; Kuder, J.F.; Murphy, S.A.; et al. Dapagliflozin and Cardiovascular Outcomes in Type 2 Diabetes. *N Engl J Med* **2019**, *380*, 347-357, doi:10.1056/NEJMoa1812389.
343. Chen, Y.; Liu, X.; Li, Q.; Ma, J.; Lv, X.; Guo, L.; Wang, C.; Shi, Y.; Li, Y.; Johnsson, E.; et al. Saxagliptin add-on therapy in Chinese patients with type 2 diabetes inadequately controlled by insulin with or without metformin: Results from the SUPER study, a randomized, double-blind, placebo-controlled trial. *Diabetes Obes Metab* **2018**, *20*, 1044-1049, doi:10.1111/dom.13161.
344. Kaku, K.; Yamada, Y.; Watada, H.; Abiko, A.; Nishida, T.; Zacho, J.; Kiyosue, A. Safety and efficacy of once-weekly semaglutide vs additional oral antidiabetic drugs in Japanese people with inadequately controlled type 2 diabetes: A randomized trial. *Diabetes Obes Metab* **2018**, *20*, 1202-1212, doi:10.1111/dom.13218.
345. Tuttle, K.R.; Lakshmanan, M.C.; Rayner, B.; Busch, R.S.; Zimmermann, A.G.; Woodward, D.B.; Botros, F.T. Dulaglutide versus insulin glargine in patients with type 2 diabetes and moderate-to-severe chronic kidney disease (AWARD-7): a multicentre, open-label, randomised trial. *Lancet Diabetes Endocrinol* **2018**, *6*, 605-617, doi:10.1016/S2213-8587(18)30104-9.
346. Groop, P.H.; Cooper, M.E.; Perkovic, V.; Hocher, B.; Kanasaki, K.; Haneda, M.; Schernthaner, G.; Sharma, K.; Stanton, R.C.; Toto, R.; et al. Linagliptin and its effects on hyperglycaemia and albuminuria in patients with type 2 diabetes and renal dysfunction: the randomized MARLINA-T2D trial. *Diabetes Obes Metab* **2017**, *19*, 1610-1619, doi:10.1111/dom.13041.
347. Holman, R.R.; Bethel, M.A.; Mentz, R.J.; Thompson, V.P.; Lokhnygina, Y.; Buse, J.B.; Chan, J.C.; Choi, J.; Gustavson, S.M.; Iqbal, N.; et al. Effects of Once-Weekly Exenatide on Cardiovascular Outcomes in Type 2 Diabetes. *N Engl J Med* **2017**, *377*, 1228-1239,

doi:10.1056/NEJMoa1612917.
348. Janssen Research & Development, L. CANVAS - CANagliflozin cardioVascular Assessment Study (CANVAS). Available online: https://clinicaltrials.gov/study/NCT01032629?cond=NCT01032629&rank=1 (accessed on 2024/12/18).
349. Janssen Research & Development, L. A Study of the Effects of Canagliflozin (JNJ-28431754) on Renal Endpoints in Adult Participants With Type 2 Diabetes Mellitus (CANVAS-R). Available online: https://clinicaltrials.gov/study/NCT01989754?cond=NCT01989754&rank=1 (accessed on 2024/12/18).
350. Del Prato, S.; Fleck, P.; Wilson, C.; Chaudhari, P. Comparison of alogliptin and glipizide for composite endpoint of glycated haemoglobin reduction, no hypoglycaemia and no weight gain in type 2 diabetes mellitus. *Diabetes Obes Metab* **2016**, *18*, 623-627, doi:10.1111/dom.12643.
351. Marso, S.P.; Daniels, G.H.; Brown-Frandsen, K.; Kristensen, P.; Mann, J.F.; Nauck, M.A.; Nissen, S.E.; Pocock, S.; Poulter, N.R.; Ravn, L.S.; et al. Liraglutide and Cardiovascular Outcomes in Type 2 Diabetes. *N Engl J Med* **2016**, *375*, 311-322, doi:10.1056/NEJMoa1603827.
352. Wang, W.; Yang, J.; Yang, G.; Gong, Y.; Patel, S.; Zhang, C.; Izumoto, T.; Ning, G. Efficacy and safety of linagliptin in Asian patients with type 2 diabetes mellitus inadequately controlled by metformin: A multinational 24-week, randomized clinical trial. *J Diabetes* **2016**, *8*, 229-237, doi:10.1111/1753-0407.12284.
353. DeFronzo, R.A.; Lewin, A.; Patel, S.; Liu, D.; Kaste, R.; Woerle, H.J.; Broedl, U.C. Combination of empagliflozin and linagliptin as second-line therapy in subjects with type 2 diabetes inadequately controlled on metformin. *Diabetes Care* **2015**, *38*, 384-393, doi:10.2337/dc14-2364.
354. Green, J.B.; Bethel, M.A.; Armstrong, P.W.; Buse, J.B.; Engel, S.S.; Garg, J.; Josse, R.; Kaufman, K.D.; Koglin, J.; Korn, S.; et al. Effect of Sitagliptin on Cardiovascular Outcomes in Type 2 Diabetes. *N Engl J Med* **2015**, *373*, 232-242, doi:10.1056/NEJMoa1501352.
355. Roden, M.; Merker, L.; Christiansen, A.V.; Roux, F.; Salsali, A.; Kim, G.; Stella, P.; Woerle, H.J.; Broedl, U.C.; investigators, E.-R.E.M. Safety, tolerability and effects on cardiometabolic risk factors of empagliflozin monotherapy in drug-naive patients with type 2 diabetes: a double-blind extension of a Phase III randomized controlled trial. *Cardiovasc Diabetol* **2015**, *14*, 154, doi:10.1186/s12933-015-0314-0.
356. Rosenstock, J.; Hansen, L.; Zee, P.; Li, Y.; Cook, W.; Hirshberg, B.; Iqbal, N. Dual add-on therapy in type 2 diabetes poorly controlled with

metformin monotherapy: a randomized double-blind trial of saxagliptin plus dapagliflozin addition versus single addition of saxagliptin or dapagliflozin to metformin. *Diabetes Care* **2015**, *38*, 376-383, doi:10.2337/dc14-1142.
357. Zinman, B.; Wanner, C.; Lachin, J.M.; Fitchett, D.; Bluhmki, E.; Hantel, S.; Mattheus, M.; Devins, T.; Johansen, O.E.; Woerle, H.J.; et al. Empagliflozin, Cardiovascular Outcomes, and Mortality in Type 2 Diabetes. *N Engl J Med* **2015**, *373*, 2117-2128, doi:10.1056/NEJMoa1504720.
358. Wysham, C.; Blevins, T.; Arakaki, R.; Colon, G.; Garcia, P.; Atisso, C.; Kuhstoss, D.; Lakshmanan, M. Efficacy and safety of dulaglutide added onto pioglitazone and metformin versus exenatide in type 2 diabetes in a randomized controlled trial (AWARD-1). *Diabetes Care* **2014**, *37*, 2159-2167, doi:10.2337/dc13-2760.
359. Lavalle-Gonzalez, F.J.; Januszewicz, A.; Davidson, J.; Tong, C.; Qiu, R.; Canovatchel, W.; Meininger, G. Efficacy and safety of canagliflozin compared with placebo and sitagliptin in patients with type 2 diabetes on background metformin monotherapy: a randomised trial. *Diabetologia* **2013**, *56*, 2582-2592, doi:10.1007/s00125-013-3039-1.
360. Rosenstock, J.; Raccah, D.; Koranyi, L.; Maffei, L.; Boka, G.; Miossec, P.; Gerich, J.E. Efficacy and safety of lixisenatide once daily versus exenatide twice daily in type 2 diabetes inadequately controlled on metformin: a 24-week, randomized, open-label, active-controlled study (GetGoal-X). *Diabetes Care* **2013**, *36*, 2945-2951, doi:10.2337/dc12-2709.
361. White, W.B.; Cannon, C.P.; Heller, S.R.; Nissen, S.E.; Bergenstal, R.M.; Bakris, G.L.; Perez, A.T.; Fleck, P.R.; Mehta, C.R.; Kupfer, S.; et al. Alogliptin after acute coronary syndrome in patients with type 2 diabetes. *N Engl J Med* **2013**, *369*, 1327-1335, doi:10.1056/NEJMoa1305889.
362. Yki-Jarvinen, H.; Rosenstock, J.; Duran-Garcia, S.; Pinnetti, S.; Bhattacharya, S.; Thiemann, S.; Patel, S.; Woerle, H.J. Effects of adding linagliptin to basal insulin regimen for inadequately controlled type 2 diabetes: a >/=52-week randomized, double-blind study. *Diabetes Care* **2013**, *36*, 3875-3881, doi:10.2337/dc12-2718.
363. Kawamori, R.; Inagaki, N.; Araki, E.; Watada, H.; Hayashi, N.; Horie, Y.; Sarashina, A.; Gong, Y.; von Eynatten, M.; Woerle, H.J.; et al. Linagliptin monotherapy provides superior glycaemic control versus placebo or voglibose with comparable safety in Japanese patients with type 2 diabetes: a randomized, placebo and active comparator-controlled, double-blind study. *Diabetes Obes Metab* **2012**, *14*, 348-

357, doi:10.1111/j.1463-1326.2011.01545.x.
364. Russell-Jones, D.; Cuddihy, R.M.; Hanefeld, M.; Kumar, A.; Gonzalez, J.G.; Chan, M.; Wolka, A.M.; Boardman, M.K.; Group, D.-S. Efficacy and safety of exenatide once weekly versus metformin, pioglitazone, and sitagliptin used as monotherapy in drug-naive patients with type 2 diabetes (DURATION-4): a 26-week double-blind study. *Diabetes Care* **2012**, *35*, 252-258, doi:10.2337/dc11-1107.
365. Chacra, A.R.; Tan, G.H.; Apanovitch, A.; Ravichandran, S.; List, J.; Chen, R.; Investigators, C.V. Saxagliptin added to a submaximal dose of sulphonylurea improves glycaemic control compared with uptitration of sulphonylurea in patients with type 2 diabetes: a randomised controlled trial. *International journal of clinical practice* **2009**, *63*, 1395-1406, doi:10.1111/j.1742-1241.2009.02143.x.
366. DeFronzo, R.A.; Hissa, M.N.; Garber, A.J.; Luiz Gross, J.; Yuyan Duan, R.; Ravichandran, S.; Chen, R.S.; Saxagliptin 014 Study, G. The efficacy and safety of saxagliptin when added to metformin therapy in patients with inadequately controlled type 2 diabetes with metformin alone. *Diabetes Care* **2009**, *32*, 1649-1655, doi:10.2337/dc08-1984.
367. Mohan, V.; Yang, W.; Son, H.Y.; Xu, L.; Noble, L.; Langdon, R.B.; Amatruda, J.M.; Stein, P.P.; Kaufman, K.D. Efficacy and safety of sitagliptin in the treatment of patients with type 2 diabetes in China, India, and Korea. *Diabetes Res Clin Pract* **2009**, *83*, 106-116, doi:10.1016/j.diabres.2008.10.009.
368. Nauck, M.A.; Meininger, G.; Sheng, D.; Terranella, L.; Stein, P.P.; Sitagliptin Study, G. Efficacy and safety of the dipeptidyl peptidase-4 inhibitor, sitagliptin, compared with the sulfonylurea, glipizide, in patients with type 2 diabetes inadequately controlled on metformin alone: a randomized, double-blind, non-inferiority trial. *Diabetes Obes Metab* **2007**, *9*, 194-205, doi:10.1111/j.1463-1326.2006.00704.x.
